# Self-domestication in *Homo sapiens*: Insights from comparative genomics

**DOI:** 10.1371/journal.pone.0185306

**Published:** 2017-10-18

**Authors:** Constantina Theofanopoulou, Simone Gastaldon, Thomas O’Rourke, Bridget D. Samuels, Angela Messner, Pedro Tiago Martins, Francesco Delogu, Saleh Alamri, Cedric Boeckx

**Affiliations:** 1 Section of General Linguistics, Universitat de Barcelona, Barcelona, Spain; 2 Universitat de Barcelona Institute for Complex Systems, Barcelona, Spain; 3 School of Psychology, University of Padova, Padova, Italy; 4 Center for Craniofacial Molecular Biology, University of Southern California, Los Angeles, CA, United States of America; 5 Department of Chemistry, Biotechnology and Food Science, Norwegian University of Life Sciences (NMBU), Ås, Norway; 6 ICREA, Barcelona, Spain; University of Colorado Boulder, UNITED STATES

## Abstract

This study identifies and analyzes statistically significant overlaps between selective sweep screens in anatomically modern humans and several domesticated species. The results obtained suggest that (paleo-)genomic data can be exploited to complement the fossil record and support the idea of self-domestication in *Homo sapiens*, a process that likely intensified as our species populated its niche. Our analysis lends support to attempts to capture the “domestication syndrome” in terms of alterations to certain signaling pathways and cell lineages, such as the neural crest.

## Introduction

Recent advances in genomics, coupled with an ever-richer body of palaeoarchaeological, anatomical, and animal behavior literature, offer new opportunities to test long-standing hypotheses about human evolution. In the domain of human cognition, the retrieval of ancient DNA can, with the help of well-articulated linking hypotheses connecting genes, brain, and cognition, shed light on the emergence of ‘cognitive modernity’. It is to this end that we present data from (paleo-)genomics in support of an old hypothesis about the evolution of our species: that of self-domestication. As has been well documented elsewhere [1, 2], the idea that anatomically modern humans (AMH) are a domesticated species has long been entertained by preeminent scholars in biological and human sciences (in passing by Charles Darwin [3] and more seriously by Franz Boas [4]). We argue that such characterizations are accurate, not merely as analogies, but in identifying shared evolutionary trajectories, with accompanying convergent signatures of selection, in AMH and domesticated species.

In order to explore whether our species is self-domesticated, we must first address what it means to be domesticated and whether AMH meet these criteria. We take the view, defended in more detail elsewhere [1, 5–7], that domesticated species are best categorized in terms of the phenotypic traits that they broadly share, rather than in terms of human mastery, design, or orchestration. There are inherent weaknesses in the human-mastery or conditions-based views of domestication that an account based on phenotypic traits does not face. The commonly shared traits of domesticates provide the strongest and most objective means by which these animals can be considered a single category. Furthermore, there is now evidence that many of the phenotypic traits of domesticates emerge independently of any human predispositions, intentional or otherwise [7, 8]. A broad consensus is now emerging that “commensal” and “mutualistic” processes can lead to domestication [6, 9–11], whereby both the domesticator and domesticated species seek out and benefit from cohabitation; thus, AMH were not the sole agents in all domestication events. Many of the species that have ultimately come to inhabit domestic niches are widely considered to have done so largely autonomously; in other words, to have self-domesticated. Changes in their social ecology (i.e., both their feeding niche and social organization), along with other parameters, have been recently suggested to confirm this hypothesis [12]. It has been proposed that dogs, cats, foxes [5, 7, 11, 13, 14], and even livestock species such as pigs, sheep, and cattle [6, 11, 15], may have undergone such processes.

Domesticated species display a range of anatomical and behavioral phenotypes that set them apart from their wild counterparts: depigmentation; floppy, reduced ears; shorter muzzles; curly tails; smaller teeth; smaller cranial capacities (and concomitant brain size reduction); paedomorphosis; neotenous (juvenile) behavior; reduction of sexual dimorphism (feminization); docility; and more frequent estrous cycles. Of course, not all of these characteristics are found in all domesticates, but many of them are indeed present to some extent in each [16]. This constellation of features has been referred to as the “domestication syndrome” and has been hypothesized to arise from a mild deficit of neural crest cells [17]. A critical question for the present study is whether our species displays some or all of the phenotypes associated with the domestication syndrome, thus warranting comparison to determine signatures of selection shared with domesticates. Such signatures of domestication can be detected through comparisons of a domesticated species with “either their direct wild-living ancestor or close relatives if the ancestor is no longer extant” [2]. In the case of AMH, since there is no wild extant counterpart available, the obvious comparanda include our closest living relatives (i.e., the great apes) and extinct species of the genus *Homo*, to the extent that relevant data can be extracted from the fossil record.

**Fig 1:**
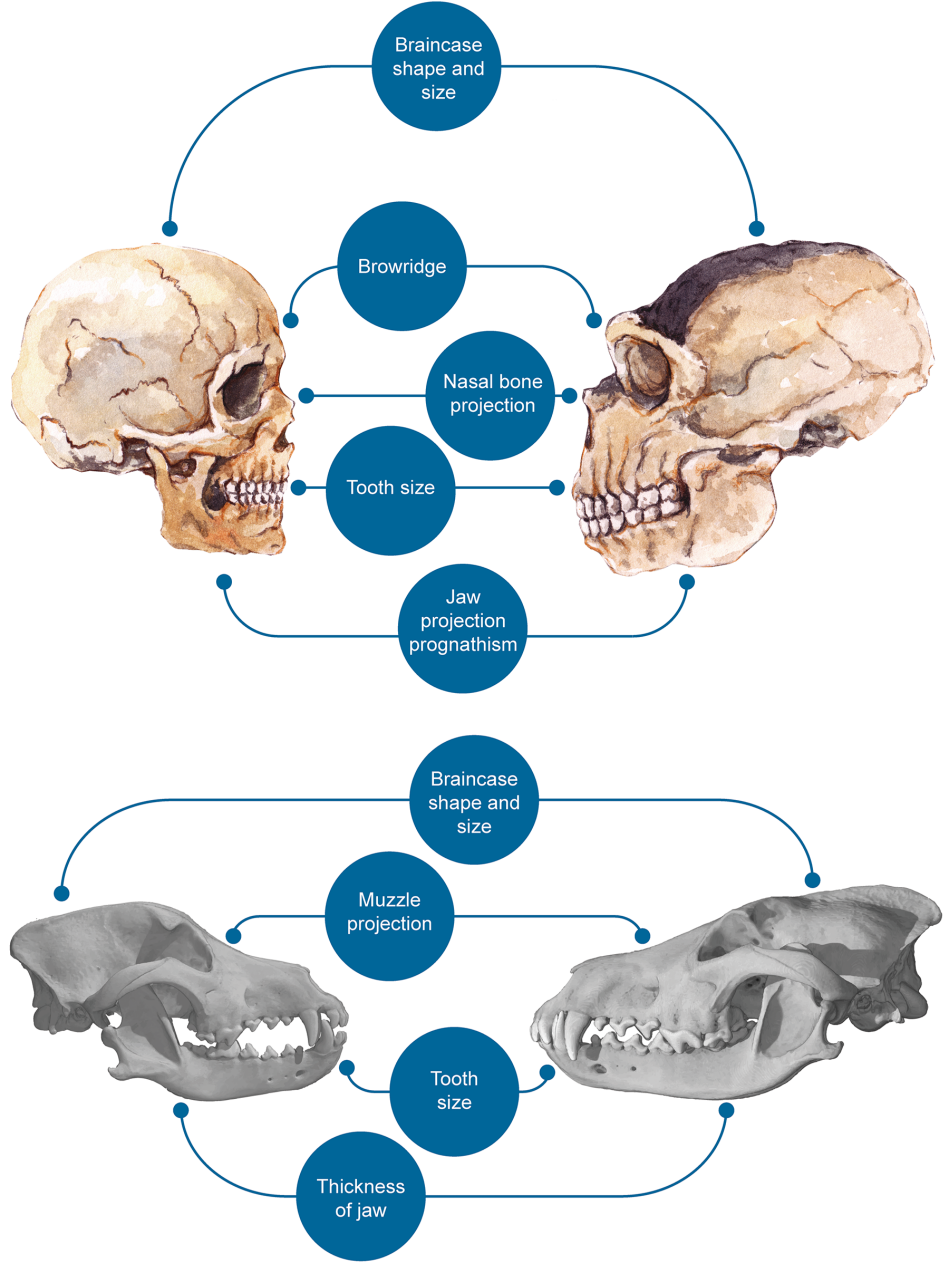
Salient craniofacial differences between AMH and Neanderthals (top) and between dogs and wolves (bottom).

Many of the anatomical changes associated with domestication describe some of the well-known anatomical differences between AMH and Neanderthals (see Fig 1). The two species display different ontogenetic trajectories [18, 19] resulting in craniofacial differences that invariably lead to a more ‘gracile’, ‘juvenile’ profile in AMH relative to Neanderthals. It is well-established that prognathism is significantly reduced in our species [19, 20]. Brow ridges and nasal projections are smaller in AMH than in our most closely related (extinct) relatives [21], as are our teeth [22, 23] and our cranial capacity [24]. This profile is sometimes called ‘feminized’ [21], and is associated with an overall reduction of sexual dimorphism, which is also associated with domestication [25]. The process of ‘feminization’ (reduction of androgen levels and rise in estrogen levels [21]) is often associated with reduced reactivity of the hypothalamus-pituitary-adrenal axis [26], a physiological trait thought to be critical for domestication [17, 27]. Evidence from digit ratio comparisons—a measure of prenatal androgen exposure [28]—further suggests that Neanderthals had higher prenatal androgen exposure than AMH [29]. Additional differences in other traits associated with domestication may exist, but there are either obvious confounding factors involved (e.g., geography for pigmentation), or the data are more controversial (as in the case of reproductive cycle changes [30]).

In light of these differences, we contend (*contra* [21]) that self-domestication coincided with the emergence of AMH (*sensu* [31]: specimens sharing a significant number of derived features in the skeleton with extant members of our species), since the critical phenotypic changes are already present in the first specimens, although this self-domestication process may have intensified as our species expanded geographically and demographically.

Having laid out the case that AMH exhibit characteristics typical of the domestication syndrome, it remains to be clarified how our self-domestication event may have occurred. An obvious difference between AMH and other (self-)domesticated species is that the selective pressures leading to our domestication must have been intraspecific, although it has been suggested that the bonobo (*Pan paniscus*), a species that displays some of the traits of the domestication syndrome, has undergone a similar self-domestication process [25]. But even *inter*specific domestication events suggest that the selective pressures for our self-domestication need not have been qualitatively different from those experienced by other species. The recent domestication of the silver fox (*Vulpes vulpes*) demonstrates this: In the experimental breeding program started by Dmitry Belyaev [7, 8, 26, 32], foxes were intensively selected and bred over more than half a century based on only one criterion, tameness towards humans. Within twenty years of selection for this trait, a range of traits typical of the domestication syndrome had emerged [8]. Crucially, this suggests that selection for tameness is enough to bring about a constellation of domestic traits (see [33]), many of which humans share. The domesticated traits exhibited by AMH plausibly emerged following similar intraspecific selective pressures for prosocial behaviors: in other words, tameness towards fellow humans. Similarly, it has been claimed that reduced emotional reactivity and increased prosociality among humans were keys to our self-domestication [34]. So, what, if anything, differentiates prosociality from self-domestication? Certainly, reduced reactivity or increased prosocial behaviors seem to be necessary precursors of self-domestication, but these are not sufficient to describe the full-blown suite of traits associated with the domestication syndrome. Only consistent selection for such behaviors has been shown experimentally to bring about the far more extensive phenotype of domestication (i.e., in the silver fox experiment), although selection for tameness exclusively does not seem to be the only pressure at work in some cases of domestication (cf. the ‘socioecological’ factor that may have shaped dog domestication [12]).

Intriguingly, there is evidence that domestication can enable the development of complex behaviors beyond those discussed so far for the domestication syndrome. For example, both dogs and domesticated foxes outperform all non-human primates in tests of cooperative communication [34]. The Bengalese finch, domesticated from its wild ancestor, the white-rumped munia [35, 36], has developed a complex song that is preferred by both female finches and munias over the stereotyped song of the male munia [37]. There are tempting parallels to be drawn here regarding the potential effects of self-domestication on the emergence of human language, relating to the emergence of a fully modern ‘language-ready’ brain [38–40], or the triggering of our capacity for complex iterative learning, necessary for the cultural transmission of language [2, 41].

The self-domestication hypothesis is, then, a strong contender to account for key aspects of modern human cognition. The central claim of the present paper is that (paleo-)genomic data can provide evidence to complement the anatomical and behavioral data outlined above, which suggest that AMH underwent a process of self-domestication. Crucially, we now have high-quality genomes for our closest extinct relatives, the Neanderthals and Denisovans, allowing for genomic comparison with AMH [42], as well as genomes of several domesticated species, which can be compared with their wild counterparts [43]. This information offers the opportunity to test for the existence of significant overlapping regions showing signatures of positive selection and putatively associated with (self-)domestication.

## Results

**Fig 2:**
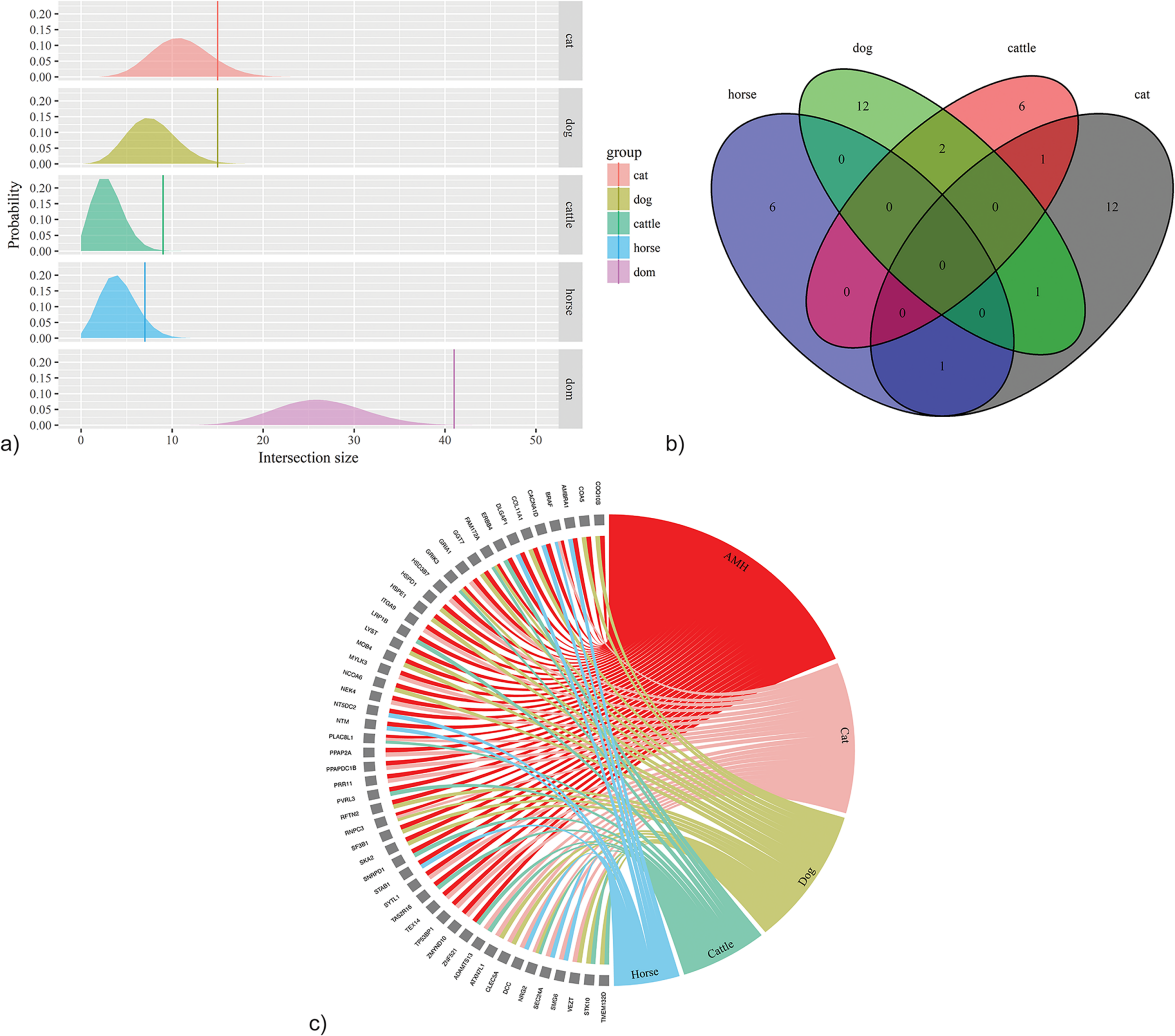
Graphical representations of overlapping genes showing signatures of positive selection in AMH and domesticated species. (a) Hypergeometric distributions for each group (individual domesticated species and the domesticate pool) with the probability of the intersection size found with AMH (*v*). cat: *v* = 15, *p* = 0.1454; dog: *v* = 15, *p* = 0.0293; cattle: *v* = 9, *p* = 0.0028; horse: *v* = 7, *p* = 0.122; dom: *v* = 41, *p* = 0.0034 (see S4 Table for details). (b) Venn diagram with the number of genes with signatures of positive selection overlapping between AMH and domesticated species. The number in each (sub)set is the number of genes showing signatures of positive selection shared by AMH and the respective species (see Table 1 and S2 Table for details). (c) Graph displaying the overlapping genes showing evidence of positive selection in AMH and one or more domesticated species (*n* = 41), and genes with evidence of positive selection in two or more domesticates (but not AMH) (*n* = 9) (see S1–S3 Tables for details).

We examined the overlap of gene sets independently claimed to be under positive selection in AMH (when compared with Neanderthal/Denisovan) and several domesticates for which detailed genetic information is available: dog (*Canis familiaris*), cat (*Felis catus*), horse (*Equus caballus*) and taurine cattle (*Bos taurus*). The pool of domesticates chosen yielded a total of 691 genes, and the total AMH pool, 742 genes. The intersection of these lists was found to be the 41 genes shown in Table 1, which represent all of the genes associated with loci under positive selection both in AMH and in one or more domesticates. A hypergeometric intersection test revealed that the intersection size of 41 was statistically significant (*p* < 0.01). The results are represented graphically in Fig 2; for further details, see S1 and S2 Tables.

**Table 1:** List of 41 overlapping genes with evidence of positive selection in AMH and domesticated species (for more details, see S2 Table).

Gene name	Overlapping species	Sources of AMH data	Sources of domesticate data
AMBRA1	horse	[44]	[45]
BRAF	cat, horse	[46]	[45, 47]
CACNA1D	horse	[48]	[45]
COA5	dog	[48]	[49]
COL11A1	dog	[46]	[50]
COQ10B	dog	[44]	[50]
DLGAP1	horse	[46]	[45]
ERBB4	cattle	[46]	[51]
FAM172A	cattle, dog	[48]	[50, 51]
GGT7	dog	[46]	[49]
GRIA1	cat	[46]	[47]
GRIK3	dog, cattle	[46]	[50]
HSD3B7	cat	[46]	[47]
HSPD1	dog	[44]	[50]
HSPE1	dog	[44]	[50]
ITGA9	cat	[48]	[47]
LRP1B	cattle	[46]	[51]
LYST	dog	[46]	[49]
MOB4	dog	[44]	[50]
MYLK3	cat	[46]	[47]
NCOA6	dog	[46]	[49]
NEK4	cat	[48]	[47]
NT5DC2	horse	[48]	[45]
NTM	horse	[46]	[45]
PLAC8L1	cat, cattle	[46]	[47, 51]
PPAP2A	cat	[48]	[47]
PPAPDC1B	cat	[44]	[47]
PRR11	cat	[48]	[47]
PVRL3	cattle	[48]	[51]
RFTN2	dog	[44]	[50]
RNPC3	cat, dog	[46]	[47, 50, 52]
SF3B1	dog	[44]	[50]
SKA2	dog	[48]	[49]
SNRPD1	cattle	[44, 46, 48]	[51]
STAB1	horse	[48]	[45]
SYTL1	cat	[48]	[47]
TAS2R16	cattle	[46]	[51]
TEX14	cat	[48]	[47]
TP53BP1	cat	[48]	[47]
ZMYND10	cat	[48]	[47]
ZNF521	cattle	[46]	[51]

We confirmed the significance of this result with a Monte Carlo simulation of 1,000,000 trials, in which samples of 691 and 742 genes were randomly selected with no replacement from a pool of 19,500 (the approximate average number of genes in the genomes of these species). The simulation confirmed that an intersection size greater than or equal to 41 is highly significant (*p* = 0.0033).

**Fig 3:**
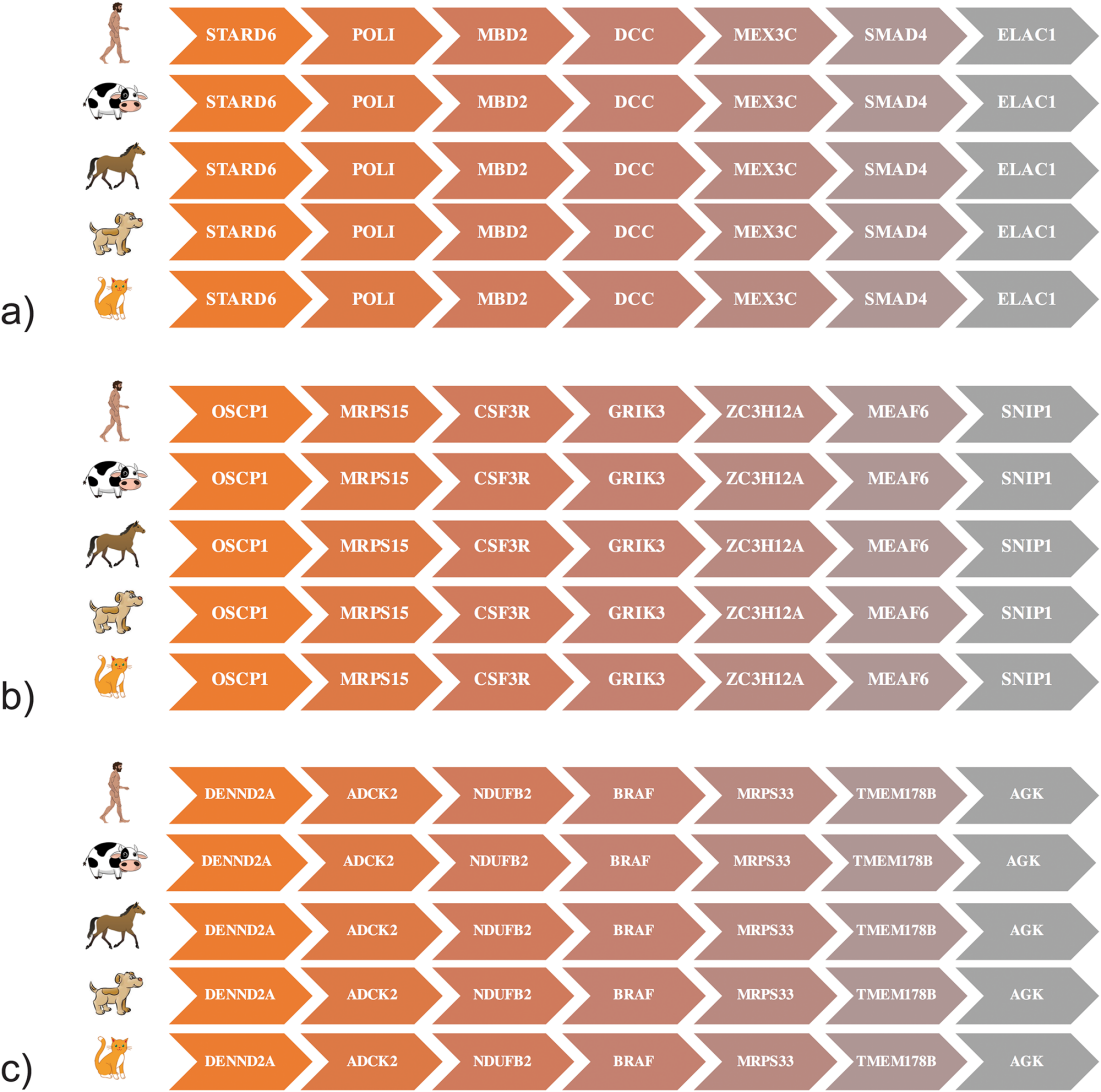
Examples of synteny analysis for 3 genes showing signatures of positive selection in AMH and domesticated species. Genes of interest (*DCC, GRIK3* and *BRAF*) and their 3 flanking protein-coding genes are shown in AMH, cattle, horse, dog and cat, illustrating their conserved syntenies. For other genes, see S6 Table.

To validate that the genes with evidence for positive selection in multiple species are orthologous (rather than simply paralogous) across the species studied, we performed synteny analysis for all 41 genes with evidence of selective sweeps in both AMH and at least one domesticate (Fig 3). We found that all 41 genes are located in syntenic blocks across the species studied (see S6 Table), meaning that the intersection size identified does not include any false positives (i.e., paralogous genes that have been given the same name due to high sequence identity). The same holds for genes associated with loci under selection in multiple domesticated species but not AMH; see again S6 Table.

This situation contrasted with the modest (statistically insignificant) overlaps between the domesticates and several Great Apes for which selective sweep screens were available: chimpanzee (*Pan t. troglodytes*), orangutan (*Pongo abelii*), and gorilla (*G. g. gorilla*) (see S4 Table).

Intersections between domesticates (15 genes in total, see S1 Table) were tested, with a hypergeometric intersection test showing a significant overlap between genes under selection in the dog and in cattle (*p* < 0.01). Furthermore, tests between AMH and each domesticate showed significant overlaps with the dog (*v* = 15, *p* < 0.05) and with cattle (*v* = 9, *p* < 0.01). In order to investigate whether these significant overlaps provide evidence for a convergent effect of domestication, we compared the pool of genes putatively under selection in AMH with genes reported to be under selection in the Eurasian wolf (*Canis lupus lupus*) and wisent (or European bison, *Bison bonasus*). These are the closest related non-domesticated species to the dog and cattle for which there are published studies of genes under selection in modern populations [53–61]. Neither wisent nor wolf populations showed any significant convergence of genes under selection with AMH (see S5 Table). This control comparison suggests that the significant overlap between AMH and both dog and cattle may be an effect of convergent domestication processes in these species.

Since we pooled data concerning positive selection and selective sweeps in AMH from different sources, intersection tests were carried out between the domestication pool and the pool of each AMH dataset used in this study. A significant intersection was found with the data from Prüfer et al. [44] (*p* < 0.05) and with the combined data from Prüfer et al. [44] and Racimo [48] (*p* < 0.05).

Though no gene was found to be shared across all domesticated species studied here as well as AMH, this is not necessarily expected. As discussed in the Introduction, domestication is known to proceed through various routes, and is thus not a uniform affair. However, common pathways can be identified, as can genes that may have contributed to domestication events that we think deserve special attention. Five genes were found to be associated with signals of positive selection in AMH and multiple domesticated species (see S3 Table): *RNPC3, FAM172A, PLAC8L1, GRIK3* and *BRAF*.

*RNPC3* shows evidence of positive selection in the dog, cat, and AMH. *RNPC3* is one of only two genes with more than one putatively causal variant fixed between dogs and wolves (the other is a gene of unknown function) [52]. Mutations in *RNPC3* cause growth hormone deficiencies in humans resulting from pituitary hypoplasia [62, 63]. In a similar vein, a gene showing an AMH-specific amino acid change and associated with a strong positive selection signal in AMH and in dogs, *NCOA6*, is a nuclear receptor coactivator that directly binds nuclear receptors and stimulates the transcriptional activities in a hormone-dependent fashion.

*FAM172A*, selected for in dogs, cattle, and AMH, may perhaps be worthy of note given its position on chromosome 5 neighboring *NR2F1*, which plays a role in regulating neural crest specifier genes and has undergone selection in AMH [64, 65]; The functionally related nuclear receptor *NR2F2* is involved in regulating embryonic stem cell differentiation [66] and implicated in neural crest development, and has been under selection in the domesticated fox [67].

*PLAC8L1* is associated with positive selection signals in cats and cattle as well as in AMH, but there is only sparse evidence concerning its function. An autistic patient has been noted as having a microdeletion at chromosome 5q32, a location which includes *PLAC8L1* [68].

The two remaining genes in S3 Table, *BRAF* and *GRIK3*, deserve special attention. They are addressed in turn below.

### ERK pathway

*BRAF*, under selection in the cat, horse, and AMH, is an important member of the ERK/MAPK signaling pathway, which has been shown to play a key role in synaptic plasticity, memory, and learning [69], and which, when disrupted, can lead to a broad range of syndromes comprising craniofacial defects and cognitive deficits [70]. *BRAF* is upstream of *ERK2*, which plays a critical role in neural crest development [71] and regulates neuronal gene expression in both the neocortex and hippocampus [69]. Both *BRAF* and *ERK2* inactivation can bring about syndromic symptoms by disrupting neural crest development [71]. *BRAF* is implicated in Noonan, Leopard, and Cardiofaciocutaneous syndromes, typical symptoms of which include prominent forehead, bitemporal narrowing, hypertelorism, and short stature, among other skeletal, cardiac, and craniofacial anomalies, frequently accompanied by moderate to severe mental retardation [72, 73]. *BRAF* interacts with other domestication-related genes, including *YWHAH* (under selection in the dog), *PPP2CA* (a neural crest-related gene, under selection in the horse), and *HER4/ERBB4*, another neural crest-related gene associated with a positive selection signal in cattle and AMH. Upstream of *BRAF*, *SOS1*, under selection in domesticated foxes, affects MAPK signaling, bringing about Noonan phenotypes [74]. Noonan syndrome-like phenotypes are associated with several genes that appear to have undergone selective sweeps in AMH. For instance, *CBL* is located in a region showing signals of a strong selective sweep in AMH compared to Altai Neanderthals [44], and, when mutated, has been shown to give rise to a Noonan syndrome-like disorder [75].

As mentioned above, the neuregulin (NRG) receptor *ERBB4*, which shows evidence of selection in humans, is part of the ERK/MAPK pathway and negatively regulates ERK via upstream phosphorylation of Raf-1 [76]. Loss of *Erbb4* function in mice has been shown to cause defects in hindbrain cranial neural crest cell pathfinding, including a caudal elongation of the trigeminal and geniculate ganglia [77]. This suggests a plausible role for *ERBB4* in preventing caudal extension in the derived AMH skull. *ERBB4* is one of many neural crest-related genes associated with selective signals in AMH (e.g., *SNAI2* [44], *CITED2* [44], *PRDM10* [46, 78], and others [38]), some of which show fixed or nearly fixed amino acid changes compared to Neanderthals. In addition, *NRG2* was the only gene that was found to be under selection in three of the four domesticated species in our study: cat, cattle, and dog. *NRG4* shows evidence of selection in cattle, and *NRG3*, in AMH. Incidentally, *NRG3* copy number and single nucleotide variants have been associated with Hirschsprung disease [79, 80]. This disease is very relevant in the context of domestication, as it affects the neural crest, associated with domestication syndrome [17, 38]. Quite a few genes associated with selective sweeps in AMH examined here (among them, *RET, ZEB2*, and *SLIT2*) have been linked to the disease [81, 82].

Enhanced ERBB4 signaling has been implicated in Angelman syndrome, an autism spectrum disorder marked by behavioral traits such as increased desire for social interaction, developmental delay, severe speech impairment, and a happy demeanour, although aggressive behavior has sometimes been reported [83–85]. Angelman-syndrome-like phenotypes are frequently associated with genes investigated here. One such observation concerns “the most intriguing variant fixed between dogs and wolves” [52], which is found in the 3’-UTR of *SLC9A6*. This gene encodes sodium/hydrogen exchanger protein 6, which is part of a network related to the plasticity of glutaminergic neurons [86]. Cagan and Blass [52] note that loss-of-function mutations in this gene in humans can lead to Christianson syndrome, also known as “Angelman-like syndrome”. Phenotypes typical of these patients include cognitive developmental delays, absence of speech, stereotyped repetitive hand movements, and postnatal microcephaly with a narrow face. Christianson syndrome is frequently characterized by a happy disposition with easily provoked laughter and smiling, an open mouth with excessive drooling and frequent visual fixation on hands. Several of these phenotypes resemble those that distinguish dogs from wolves.

We used Ingenuity Pathway Analysis software (QIAGEN, Redwood City, CA) to perform pathway analyses on the lists of genes in S1 and S2 Tables, as well as on the list of genes with amino acid replacement substitutions fixed in AMH and absent in archaic humans [87]. These analyses involved mining a database of literature on known interactions between the genes in each of these sets, and revealed that ERKs are among the most significant downstream targets of the interacting selected genes in each (see S1 Fig). Incidentally, an analysis of a domesticated pig (*Sus scrofa domesticus*) genome [88] suggests that the involvement of ERK pathway in domestication extends beyond the species we focused on in this study.

As a final note on the ERK pathway, we would like to highlight the presence of *CACNA1D* in S2 Table *CACNA1D* is a neural cell adhesion molecule that contributes to cell migration via activation of MAPK/ERK signaling [89]. This gene is one of several axon-guidance molecules we identified in our study. It is highly expressed in the adrenal glands [90], and, when mutated, gives rise to cerebral palsy/motor disorders [91]. It has been linked to auditory processing [92], and said to be among the positively selected genes in some vocal learners [93].

### Glutamate receptors

The glutamate receptor *GRIK3* has been associated with positive selection signals in AMH, dog, and cattle, and interacts with other glutamate receptors associated with positive selection signals in the horse (*GRID1*) and cat (*GRIA1/2*). Polymorphisms in *GRIK3* and *GRID1* have been implicated in schizophrenia [94, 95], and *GRID1* neighbors *NRG3* (discussed above) at the schizophrenia susceptibility loci 10q22-q23 [96]. Developmental delays and craniofacial anomalies associated with a loss of genetic material at the *NRG3* locus, accompanied by a gain of material at the *DLGAP1* site, have also been reported [97]. *DLGAP1*, a scaffold-protein-coding gene at the postsynaptic density, is under selection in AMH and in the horse. This gene has been implicated in obsessive-compulsive disorders and interacts significantly with Shank proteins, mutations in which have been linked to autism spectrum disorders with impaired social interaction and communication [98–100]. DLGAP1 interacts with the glutamate receptor GRIK2, also implicated in obsessive-compulsive disorders [101].

Previous work by Li et al. [102] already pointed out that genes involved in glutamate metabolism show the greatest population differentiation by whole-genome comparison of dogs and wolves. Although such changes may be implicated in fear response differences between the dog and the wolf populations, Li et al. argue for a role in increasing excitatory synaptic plasticity in dogs rather than reducing fear response. As they point out, changes related to synaptic plasticity may have a significant impact on learning and memory. This is certainly true for cognitive specializations in humans, like language, since glutamate receptors have been shown to be differentially regulated in brain regions associated with vocal learning [103].

### Genes under selection in multiple domesticates but not AMH

It is worth considering those genes under selection across domesticates, independently of their selection in AMH, for different reasons. First, our aim here is to explore the extent to which self-domesticating processes in humans may have contributed to our species’ anatomical, cognitive, and behavioral make-up. Uncovering genes of interest for (albeit often different) domesticating processes in other well-studied domesticated species is a promising way to pursue this goal. The most interesting genes should be those that are associated with positive selection signals across different species. Those genes under selection only in certain domesticates, but which strongly interact with genes under selection in other domesticated species, may prove central to a relevant domesticating process, given that these interactions may shed special light on relevant phenotypic traits. Similarly, certain genes associated with positive selection across different domesticates may have strong interactions with other genes that are under selection in AMH. We wish to highlight some of these here (for a full list, see S1 Table).

*DCC* (*DCC Netrin 1 receptor*), an axon-guidance mediator and neural crest-related gene, which shows signatures of positive selection in both the horse and the cat, interacts strongly with *DSCAM* (*Down Syndrome cell adhesion molecule*), another axon-guidance and neural crest-related gene, selected for in cattle. Of key significance is the interaction of *DCC* with the Slit/Robo pathway, especially given the proposed involvement of this pathway in vocal learning [104, 105] and the selection of both *ROBO2* and *SLIT2* in AMH [46]. The related gene *ROBO1* also shows evidence of selection in cattle. ROBO silences the attractive effect that Netrin 1 has on DCC, allowing SLIT2 to bind to this ligand and enabling axon pathfinding in the developing brain [106, 107]. *DCC* is involved in the organization of dopaminergic circuits within the cortex [108], and several association studies have identified *DCC* as a promising candidate for schizophrenia [109]. Importantly, an AMH-specific hCONDEL exists in a region upstream of *DCC*, although it is shared with Neanderthals [110]. However, a detailed examination of this gene on both the modern and archaic lines, reveals an accummulation of changes on this gene in AMH. In addition, several genes showing AMH-specific amino-acid substitutions, such as *NOVA1* and *RASA1*, both involved in neuronal development, are known to interact with *DCC* [111, 112], and could regulate it in a species-specific fashion. *RASA1* is associated with a strong selective signal in AMH, and has been shown to mediate Netrin 1-induced cortical axon outgrowth and guidance [112]. Together with the glutamate receptor changes discussed above, such modifications may have played an important role in generating aspects of the cognitive profile associated with modern humans, including a full-fledged language-ready brain.

We found several collagen-type genes with signatures of selection across domesticates. *COL22A1*, a gene under selection in the horse, significantly interacts with various similar genes associated with positive selection signals in other domesticates, particularly in the cat, including *COL11A1*, under selection in the dog and AMH. *COL22A1* and *COL11A1* exhibit increased expression in the bone tissue and hippocampus of mice with some of the symptoms of Kleefstra Syndrome (developmental delay, hypotonia, and craniofacial abnormalities), which is often accompanied by autistic symptoms and intellectual disability in humans [113, 114].

### Archaic-derived alleles

To the best of our knowledge, no comprehensive selective sweep analysis exists for Neanderthals. We examined the genes associated with archaic-derived alleles [115] and found that no genes in S1 Table display reported archaic-derived alleles. While this could be due to the modest number of archaic-specific SNCs known at the time of writing, we find this to be an important contrast with the situation that obtains with AMH, in light of the self-domestication hypothesis. It is striking that Castellano et al. [115] highlight genes involved in skeletal development and associated with aggressive phenotypes in their comparison of archaic *Homo* and AMH.

We also examined data concerning nearly fixed ancestral or derived SNPs in archaic lineages that crop up as variants in modern-day populations. Despite the many confounding factors as to how the relevant mutated genes might interact in different genetic contexts, one might still expect certain archaic-selected SNPs to exhibit somewhat ‘underdomesticated’ phenotypes when occurring as AMH variants. In this sense, mutations imitating ancestral SNPs found in archaic lineages may be able to tell us a great deal about the evolution of our lineage, by allowing us to glimpse some aspects of the ancestral genotype. Among those mutations we found through an exhaustive literature review, there is an ancestral S330A mutation of *SLITRK1* that may be involved in obsessive-compulsive disorders like Tourette’s Syndrome [116, 117]. Different amino acid changes around the site of an AMH-specific derived protein, ADSL (A429V), can bring about adenylosuccinate lyase deficiency (R426H; D430N [118]), the symptoms of which include developmental delay, autistic-like traits, aggressiveness, and microcephaly [119].

## Discussion

As already mentioned in the Introduction, several scholars have pointed out that there are several routes to domestication. We should therefore expect genes targeted by domestication processes to differ considerably across species. Nevertheless, reviewing the molecular events associated with domestication reveals common themes, with significant numbers of genes related to brain function and behavior, anatomy, and diet, across domesticates. This is consistent with the view that domestication may be best represented as a spectrum or continuum [120], with a polygenic basis and non-uniform symptomatology. This state of affairs is reflected in significant brain gene expression differences across domesticates, with the majority of these changes being species-specific [121].

Because of these findings, we find the overlaps listed in S1 and S2 Tables and the associated functions and pathways discussed in the Results section all the more relevant, especially because they converge to a large extent with what is to be expected from the neural crest-based hypothesis [17] put forth to capture the common mechanistic basis of domestication events. A disruption in neural crest developmental programs might be the source of changes spanning multiple organ systems and morphological structures [17], and the genes examined here seem to broadly support this view. It is quite possible that a neural crest-based explanation won’t apply to all domesticates [16], but it is interesting that this hypothesis finds its strongest support in species like dogs (see also [122]), which have been argued to be self-domesticated [34]. Recall that the goal of the present study was not to provide molecular evidence for a general theory of domestication, but rather to identify domestication-related pathways that could be suggestive of a self-domestication process in AMH. The fact that we find neural crest-related changes in AMH compared to Neanderthals/Denisovans, and that such changes are also found in another species hypothesized to have undergone a self-domestication process, reinforces our hypothesis that self-domestication took place in our species.

Apart from neural crest-related genes and pathways, we identified common themes pertaining to neuronal development, synaptic plasticity, and enhanced learning. These categories are often mentioned in studies on selective sweeps in AMH (e.g., [46]). These results are in line with claims in other studies on domestication [49, 123–125], where categories like ‘neurological process’ frequently stand out strongly in gene ontology category enrichment analyses. This potentially lends credence to claims pairing domestication and a certain type of intelligence [126]. It is also not unreasonable to suspect that byproducts of the domestication process, such as enhanced sensory-motor perceptual and learning pathways, may provide a foundation for more complex communicative abilities, including vocal learning abilities [39, 127].

In a similar vein, among the genes under selection in both AMH and one or more domesticates, as well as in those under selection in multiple domesticates though not AMH, one finds multiple strong candidates for neurodevelopmental diseases and syndromes (see also [128]). This could be seen as an additional piece of evidence suggestive of a self-domestication process in AMH. A build-up of deleterious alleles is documented across domesticated species when compared to their wild counterparts. For instance, there is a higher frequency of non-synonymous substitutions in the nuclear DNA of domesticated dogs relative to gray wolves [129], and the same is true of their mitochondrial DNA [130]. A higher frequency of non-synonymous substitutions in domesticated yaks compared to the wild yaks has also been reported [131]. This build-up of deleterious alleles has been described as the ‘cost of domestication’ [132], which, if true, could be a byproduct of self-domestication in AMH, too.

A study like the present one suffers from several limitations. While we have tried to make our comparisons as fair as possible, we have relied on genomic data that necessarily reflect the current state of the art for the various species we examined. The lists of genes associated with signals of positive selection are derived from the literature, and were generated using different analytical tools. While we have done our best to minimize the number of simplifying assumptions (see Methods), we must point out that even within a single species (e.g., AMH), no two studies completely agree on a definitive list. Indeed, in some cases, they produce lists of very different sizes. In addition, we may have missed important genes of interest due to the lack of information on them in the various databases we consulted. While it is to be hoped that some of these limitations will be overcome in the future, we think that the overlaps discussed in this study should encourage further detailed examination of these genes and the processes in which they take part. Last, but not least, it remains to be determined experimentally that the overlaps discovered here are indeed associated with mutations that led to similar functional effects across species.

We could have been more strict about our notion of convergence, and restrict our attention to genes where the exact same difference (e.g., the same amino acid substitution) could be detected across species (for an early attempt along these lines, see [133]). But given that convergent evolution is often hypothesized to occur in the absence of this very strict notion of convergence—for instance, convergent evolution in the domain of vocal learning is related to non-identical changes in *FOXP2* across vocal learners [134]—we feel justified in our approach.

## Methods

### Data

To identify signatures of a self-domestication process in AMH, we first constructed a list of genes associated with signs of positive selection in AMH compared to Neanderthals and Denisovans, which yielded a total of 742 genes. We then compared this list to the genes independently argued to be associated with positive selection in domesticated species versus their wild counterparts, which numbered 691 in total, and examined the overlap between these two gene lists.

For AMH-Neanderthal/Denisovan comparisons, we made use of findings based on high-quality genome reconstructions, specifically: the list of genes in regions of putative selective sweeps, together with pathway and disease annotation, of Prüfer et al. [44]; the list of genes from the top 20 candidate regions for the modern human ancestral branch in the work of Racimo [48]; and the extended list of genomic regions predicted to underlie positively selected human specific traits by Peyrégne et al. [46].

We included in our study a range of domesticated species for which detailed genetic information is available. These species offer representative examples of the various routes to domestication [11], as well as different temporal windows for domestication. The species include: dog (*Canis familiaris*) [49, 50, 52], cat (*Felis catus*) [47], horse (*Equus caballus*) [45], and taurine cattle (*Bos taurus*) [51]. We homogenized the nomenclature across gene sets as best we could.

We also examined other species, including the rabbit (*Oryctolagus cuniculus*) [125], and bonobo (*Pan paniscus*) [135]. In the end, the lists of genes under selection for these species (compared to their wild counterparts) were too small to draw any firm conclusions.

To help us understand domestication-related changes better, we made use of the comparison of two lines of rats (*Rattus norvegicus*) selected for tame and aggressive behaviour to identify genetic loci that differ between the lines [136], the comparison of gene expression levels in the brains of domesticated and wild animals [121], genomic signatures of domestication in neurogenetic genes in *Drosophila melanogaster* (in which neurogenetic genes have been claimed to be associated with signs of positive selection [123]), and the genetic divergence between foxes (*Vulpes vulpes*) that were selected for tame and aggressive behavior [67].

For the Great Ape comparison—chimpanzee (*Pan t. troglodytes*), orangutan (*Pongo abelii*), and gorilla (*G. g. gorilla*)—we made use of positive and balancing selection and selective sweep data from Cagan et al. [137] (Tables S6, S18(68), S19(69), S20(70), S24(74), and S97).

For AMH comparisons with the Eurasian wolf (*Canis lupus lupus*) we used data from Stronen et al. [54] (Tables 2, S3, and S5: genes under selection associated with environmental and geographic variables or with no obvious spatial patterns) and Pilot et al. [53] (Table S4: genes adjacent to loci putatively under selection in European wolves). For the wisent (*Bison bonasus*) we used data from Gautier et al. [55] (Table S3: genes under positive selection between the wisent and bovine lineages) and Wang et al. [56] (Table S14: genes under positive selection in the wisent).

### Methods

In order to test the significance of the overlap between domestication-related genes and genes showing signals of positive selection and selective sweep in AMH, a hypergeometric intersection test was performed using the *R* software [138] and the *R* package hint [139]. A hypergeometric intersection distribution can be employed to compute the probability of picking an intersection of size *v* when drawing independently and without replacement from two sets *A* and *B* composed of objects of *n* categories, with *a* and *b* number of draws, respectively (where *a* ≠ *b*) [139].

As a model of our data we chose as a simplifying assumption *n* = 19,500 as the average number of protein-coding genes for all the species taken into consideration. From the original lists, we removed antisense RNA genes (non coding), miRNAs, and other non-coding transcripts/products listed in the original tables.

From this modeled genome, a total of *a* = 691 genes were drawn from the domesticate pool (comprising cat, dog, cattle, and horse), while *b* = 742 genes were drawn from the total AMH pool. The resulting intersection size (i.e., the number of genes associated with positive selection signals both in AMH and in one or more domesticate) was *v* = 41. The hint.test function was then employed to test the significance of this intersection, obtaining *p* < 0.01.

A Monte Carlo simulation was performed using Matlab (MathWorks, Natick, MA) to confirm these results. Two random samples, of lengths 691 and 742 (with no replacement), were drawn from a pool representing 19,500 genes using Matlab’s random number generation function. These simulated draws were performed 1,000,000 times and the percentage of trials in which the intersection was ≥41 was calculated. The results revealed that 0.33% of trials had intersections of this size.

Since we pooled data for positive selection and selective sweep in AMH from different sources, hypergeometric intersection tests were carried out between the domestication pool and the pool of each AMH dataset used in this study. A significant intersection was found with the data in [44] (*a* = 691, *b* = 108, *v* = 9; *p* < 0.05) and with the combined data from [44] and [48] (*a* = 691, *b* = 419, *v* = 24, *p* < 0.05).

Overlaps with domesticates were tested for Great Apes, using data from Cagan et al. [137]. For chimpanzee (*Pan t. troglodytes*), *b* = 415 with *v* = 16; for orangutan (*Pongo abelii*), *b* = 500 with *v* = 20; for gorilla (*G. g. gorilla*), *b* = 426 with *v* = 12. The hypergeometric intersection tests yielded non-significant results for all these intersections. Monte Carlo simulations, performed as described above, *mutatis mutandis*, showed that intersections of these sizes occurred in a large fraction of trials (40.11% of trials for chimpanzee; 32% for orangutan; 82.89% for gorilla). As in the case of AMH, overlaps with individual domesticates were tested, with no significant results.

We tested overlaps with the Eurasian wolf (*Canis lupus lupus*) using data from Stronen et al. [54] (Table 2: *b* = 32 with *v* = 3, S3: *b* = 70 with *v* = 0, S5: *b* = 33 with *v* = 1) and [140] (*b* = 32 with *v* = 1). For the wisent (*Bison bonasus*) we tested overlaps using data from Gautier et al. [55] (*b* = 425 with *v* = 11) and Wang et al. [56] (*b* = 72 with *v* = 3). None of the overlaps between these non-domesticated species and AMH were significant.

For synteny analysis, we used the genomic data available for each species in the NCBI (https://www.ncbi.nlm.nih.gov/) and Ensemble (http://www.ensembl.org/index.html) databases. In S1 Table, for each of the 41 overlapping genes we included the 4 protein-coding genes flanking the region of interest. We added more flanking protein-coding genes only in the instances where some event (e.g., gene insertion or local duplication) rendered the synteny less clear. We also used NCBI Gene Search and BLAST (https://blast.ncbi.nlm.nih.gov/Blast.cgi) to confirm that some of the genes surrounding the genes of interest were the same genes across taxa, with different names in some species’ assemblies.

We then examined the functions of the genes in S1, S2 and S3 Tables, paying close attention to the pathways in which they are involved, and to their interactions with other genes already highlighted in the domestication literature. In addition to performing an exhaustive PubMed (http://www.ncbi.nlm.nih.gov/pubmed) search on each of the genes, we drew upon the information available in Genecards (http://genecards.org), Uniprot (http://www.uniprot.org/), String 10.0 (http://string-db.org), and Biogrid 3.4 (http://thebiogrid.org) to identify potential protein-protein interactions and Gene Ontology category enrichment signals. Additionally, we fed the gene lists in S1 and S2 Tables into Ingenuity Pathway Analysis software (QIAGEN, Redwood City, CA) and used the Core Analysis tools to study the associated gene networks and functions. The two major networks generated by these analyses, in which the centrality of the ERK pathway is visible, are provided S1 Fig.

Furthermore, we gathered information about the expression patterns of these genes, concentrating on those genes with relatively high expression in tissues such as brain, bone, and adrenal glands. For this, we relied on the following resources: Brainspan (http://www.brainspan.org), Human Brain Transcriptome (http://hbatlas.org), Bgee (http://bgee.org), Proteomics DB (https://proteomicsdb.org), Human Protein Atlas (http://www.proteinatlas.org), Gene Enrichment Profiler (http://xavierlab2.mgh.harvard.edu/EnrichmentProfiler/index.html), and GTex (http://www.gtexportal.org). For the information presented in the Supplementary Material, we consulted the following databases: KEGG Pathways and Disease (http://www.kegg.jp/kegg/), PANTHER (http://www.pantherdb.org), Reactome Pathway Database (http://www.reactome.org), OMIM (http://omim.org), and MalaCards (http://www.malacards.org/).

## Supporting information

S1 Fig Networks of overlapping genes (a) between domesticated species and (b) between AMH and domesticated species, generated using QIAGEN Ingenuity Pathway Analysis software. (PDF)

S1 Table Genes overlapping between at least two domesticated species. (PDF)

S2 Table Genes overlapping between AMH and domesticated species. (PDF)

S3 Table Genes overlapping between AMH and two or more domesticated species. (PDF)

S4 Table Gene lists from the sources for AMH, domesticated species, and great apes. Gene lists and statistical analysis of data from domesticates, between AMH and each domesticate, and between great apes and domesticates.
(PDF)

S5 Table Genes overlapping between AMH, non-domesticated Canis (grey wolf), and non-domesticated bovine (wisent, European bison). Gene lists and statistical analysis of data from AMH and non-domesticated species.
(PDF)

S6 Table Synteny analysis of the genes overlapping between AMH and domesticated species, and those under selection in multiple domesticated species. (PDF)
