# Correction: Self-domestication in *Homo sapiens*: Insights from comparative genomics

**DOI:** 10.1371/journal.pone.0196700

**Published:** 2018-05-11

**Authors:** Constantina Theofanopoulou, Simone Gastaldon, Thomas O’Rourke, Bridget D. Samuels, Pedro Tiago Martins, Francesco Delogu, Saleh Alamri, Cedric Boeckx

Angela Messner should not be listed as an author on this article. The correct author byline is: Constantina Theofanopoulou, Simone Gastaldon, Thomas O’Rourke, Bridget D. Samuels, Pedro Tiago Martins, Francesco Delogu, Saleh Alamri, Cedric Boeckx. The correct citation is: Theofanopoulou C, Gastaldon S, O’Rourke T, Samuels BD, Martins PT, Delogu F, et al. (2017) Self-domestication in *Homo sapiens*: Insights from comparative genomics. PLoS ONE 12(10): e0185306. https://doi.org/10.1371/journal.pone.0185306
