# Supplementary material for: Self-domestication in Homo sapiens: Insights from comparative genomics
Source: PLoS One. 2017 Oct 18;12(10):e0185306. doi: 10.1371/journal.pone.0185306 (PMC5646786; doi:10.1371/journal.pone.0185306)

S1 Fig. Networks of overlapping genes (a) between domesticated species and (b) between AMH and domesticated species, generated using QIAGEN Ingenuity Pathway Analysis software.

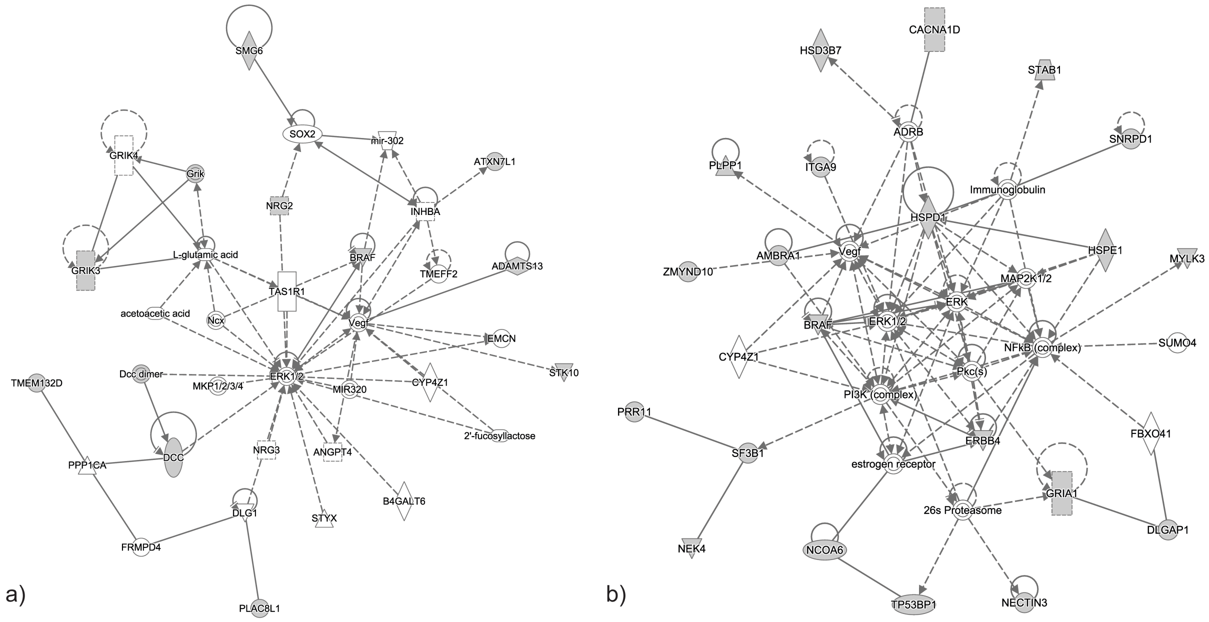

Supplement: S1 Fig — (PDF) [file pone.0185306.s001.pdf]
