# Supplementary material for: Self-domestication in Homo sapiens: Insights from comparative genomics
Source: PLoS One. 2017 Oct 18;12(10):e0185306. doi: 10.1371/journal.pone.0185306 (PMC5646786; doi:10.1371/journal.pone.0185306)
Supplement: S1 Table — (PDF) [file pone.0185306.s002.pdf]

S1 Table. Genes overlapping between at least two domesticated species

GENE NAME	*OVERLAPPING SPECIES*	*ENSEMBL ID*	*GENE FUNCTION (UniProt)*	*PATHWAY ANNOTATION*	*RELATED DISORDERS*
ADAMTS13	cattle, cat	ENSG00000160323	Cleaves the vWF multimers in plasma into smaller forms thereby controlling vWF-mediated platelet thrombus formation.	Metabolism of proteins (Reactome)	Thrombotic thrombocytopenic purpura, familial (OMIM)
ATXN7L1	cat, dog	ENSG00000146776	N/A	N/A	N/A
BRAF	cat, horse	ENSG00000157764	Protein kinase involved in the transduction of mitogenic signals from the cell membrane to the nucleus. May play a role in the postsynaptic responses of hippocampal neuron. Phosphorylates MAP2K1, and thereby contributes to the MAP kinase signal transduction pathway.	EGFR tyrosine kinase inhibitor resistance, Endocrine resistance, MAPK signaling pathway, ErbB signaling pathway, Rap1 signaling pathway, cAMP signaling pathway, Chemokine signaling pathway, FoxO signaling pathway, mTOR signaling pathway, Vascular smooth muscle contraction, Dorso-ventral axis formation, Focal adhesion, Natural killer cell mediated cytotoxicity, Long-term potentiation, Neurotrophin signaling pathway, Serotonergic synapse, Long-term depression, Regulation of actin cytoskeleton, Insulin signaling pathway, Progesterone-mediated oocyte maturation, Alcoholism, Hepatitis C, Pathways in cancer, Proteoglycans in cancer, Colorectal cancer, Renal cell carcinoma, Pancreatic cancer, Endometrial cancer, Glioma, Prostate cancer, Thyroid cancer, Melanoma, Bladder cancer, Chronic myeloid leukemia, Acute myeloid leukemia, Non-small cell lung cancer, Breast cancer (KEGG); VEGF signaling pathway, T cell activation, Interleukin signaling pathway, Inflammation mediated by chemokine and cytokine signaling pathway, Integrin signalling pathway, EGF receptor signaling pathway, Ras Pathway, Angiogenesis, CCKR signaling map, PDGF signaling pathway, B cell activation (PANTHER).	Thyroid cancer, Malignant melanoma, Noonan syndrome and related disorders, Langerhans cell histiocytosis (KEGG); Adenocarcinoma of lung, somatic; Cardiofaciocutaneous syndrome; Colorectal cancer, somatic; LEOPARD syndrome; Melanoma, malignant, somatic; Nonsmall cell lung cancer, somatic; Noonan syndrome (OMIM)
CLEC5A	dog, cat	ENSG00000258227	Functions as a positive regulator of osteoclastogenesis. Cell surface receptor that signals via TYROBP. Regulates inflammatory responses. Acts as a key regulator of synovial injury and bone erosion during autoimmune joint inflammation (By similarity). Critical macrophage receptor for dengue virus serotypes 1-4.	Immune System (Reactome)	N/A
DCC	horse, cat	ENSG00000187323	Receptor for netrin required for axon guidance. Mediates axon attraction of neuronal growth cones in the developing nervous system upon ligand binding. Its association with UNC5 proteins may trigger signaling for axon repulsion. It also acts as a dependence receptor required for apoptosis induction when not associated with netrin ligand. Implicated as a tumor suppressor gene.	Axon guidance mediated by netrin, Axon guidance mediated by Slit/Robo (PANTHER)	Colorectal cancer, somatic; Esophageal carcinoma, somatic; Mirror movements (OMIM)
FAM172A	dog, cattle	ENSG00000113391	N/A	N/A	N/A
GRIK3	dog, cattle	ENSG00000163873	Receptor for glutamate that functions as ligand-gated ion channel in the central nervous system and plays an important role in excitatory synaptic transmission. L-glutamate acts as an excitatory neurotransmitter at many synapses in the central nervous system. The postsynaptic actions of Glu are mediated by a variety of receptors that are named according to their selective agonists.	Neuroactive ligand-receptor interaction; Glutamatergic synapse (KEGG); Huntington disease, Ionotropic glutamate receptor pathway, Metabotropic glutamate receptor group III pathway (PANTHER)	schizophrenia, obsessive-compulsive disorder, alcohol dependence (MalaCards)
NRG2	dog, cat, cattle	ENSG00000158458	Direct ligand for ERBB3 and ERBB4 tyrosine kinase receptors. Concomitantly recruits ERBB1 and ERBB2 coreceptors, resulting in ligand-stimulated tyrosine phosphorylation and activation of the ERBB receptors. May also promote the heterodimerization with the EGF receptor.	EGF receptor signaling pathway (PANTHER), EGFR tyrosine kinase inhibitor resistance, ErbB signaling pathway (KEGG),	Hereditary motor and sensory neuropathy (Charcot-Marie-Tooth disease) (MalaCards)
PLAC8L1	cattle, cat	ENSG00000173261	N/A	N/A	N/A
RNPC3	dog, cat	ENSG00000185946	Participates in pre-mRNA U12-dependent splicing, performed by the minor spliceosome which removes U12-type introns. U12-type introns comprises less than 1% of all non-coding sequences. Binds to the 3'-stem-loop of m7G-capped U12 snRNA.	Gene Expression (Reactome)	growth hormone deficiency, parainfluenza virus type 3, idiopathic inflammatory myopathy, rheumatic disease (MalaCards)
SEC24A	cat, horse	ENSG00000113615	Component of the COPII coat, that covers ER-derived vesicles involved in transport from the endoplasmic reticulum to the Golgi apparatus. COPII acts in the cytoplasm to promote the transport of secretory, plasma membrane, and vacuolar proteins from the endoplasmic reticulum to the Golgi complex.	Protein processing in endoplasmic reticulum (KEGG)	N/A
SMG6	cat, horse	ENSG00000070366	Component of the telomerase ribonucleoprotein (RNP) complex that is essential for the replication of chromosome termini. May have a general role in telomere regulation. Promotes in vitro the ability of TERT to elongate telomeres. Overexpression induces telomere uncapping, chromosomal end-to-end fusions (telomeric DNA persists at the fusion points) and did not perturb TRF2 telomeric localization. Binds to the single-stranded 5'-(GTGTGG)4GTGT-3' telomeric DNA, but not to a telomerase RNA template component (TER). Plays a role in nonsense-mediated mRNA decay. Is thought to provide a link to the mRNA degradation machinery as it has endonuclease activity required to initiate NMD, and to serve as an adapter for UPF1 to protein phosphatase 2A (PP2A), thereby triggering UPF1 dephosphorylation. Degrades single-stranded RNA (ssRNA), but not ssDNA or dsRNA.	mRNA surveillance pathway (KEGG)	tricuspid valve insufficiency (MalaCards)
STK10	dog, cattle	ENSG00000072786	Serine/threonine-protein kinase involved in regulation of lymphocyte migration. Phosphorylates MSN, and possibly PLK1. Involved in regulation of lymphocyte migration by mediating phosphorylation of ERM proteins such as MSN. Acts as a negative regulator of MAP3K1/MEKK1. May also act as a cell cycle regulator by acting as a polo kinase kinase: mediates phosphorylation of PLK1 in vitro; however such data require additional evidences in vivo. (UniProt)	N/A	Testicular germ cell tumor, seminal vescicle tumor, cecal disease, anus disease, biliary dyskinesia (MalaCards)
TMEM132D	dog, cattle	ENSG00000151952	May serve as a cell-surface marker for oligodendrocyte differentiation.	N/A	pthirus pubis infestation, lice infestation, parasitic ectoparasitic infectious disease (MalaCards)
VEZT	dog, cat	ENSG00000028203	Plays a pivotal role in the establishment of adherens junctions and their maintenance in adult life. In case of Listeria infection, promotes bacterial internalization by participating in myosin VIIa recruitment to the entry site.	N/A	N/A
