# Supplementary material for: Self-domestication in Homo sapiens: Insights from comparative genomics
Source: PLoS One. 2017 Oct 18;12(10):e0185306. doi: 10.1371/journal.pone.0185306 (PMC5646786; doi:10.1371/journal.pone.0185306)
Supplement: S2 Table — (PDF) [file pone.0185306.s003.pdf]

S2 Table. Genes overlapping between AMH and domesticated species

GENE NAME	*OVERLAPPING SPECIES*	*ENSEMBL ID*	*GENE FUNCTION (UniProt)*	*PATHWAY ANNOTATION*
AMBRA1	horse	ENSG00000110497	Regulates autophagy and development of the nervous system. Involved in autophagy in controlling protein turnover during neuronal development, and in regulating normal cell survival and proliferation (By similarity).	Cellular responses to stress (Reactome)
BRAF	cat, horse	ENSG00000157764	Protein kinase involved in the transduction of mitogenic signals from the cell membrane to the nucleus. May play a role in the postsynaptic responses of hippocampal neuron. Phosphorylates MAP2K1, and thereby contributes to the MAP kinase signal transduction pathway.	EGFR tyrosine kinase inhibitor resistance, Endocrine resistance, MAPK signaling pathway, ErbB signaling pathway, Rap1 signaling pathway, cAMP signaling pathway, Chemokine signaling pathway, FoxO signaling pathway, mTOR signaling pathway, Vascular smooth muscle contraction, Dorso-ventral axis formation, Focal adhesion, Natural killer cell mediated cytotoxicity, Long-term potentiation, Neurotrophin signaling pathway, Serotonergic synapse, Long-term depression, Regulation of actin cytoskeleton, Insulin signaling pathway, Progesterone-mediated oocyte maturation, Alcoholism, Hepatitis C, Pathways in cancer, Proteoglycans in cancer, Colorectal cancer, Renal cell carcinoma, Pancreatic cancer, Endometrial cancer, Glioma, Prostate cancer, Thyroid cancer, Melanoma, Bladder cancer, Chronic myeloid leukemia, Acute myeloid leukemia, Non-small cell lung cancer, Breast cancer (KEGG); VEGF signaling pathway, T cell activation, Interleukin signaling pathway, Inflammation mediated by chemokine and cytokine signaling pathway, Integrin signalling pathway, EGF receptor signaling pathway, Ras Pathway, Angiogenesis, CCKR signaling map, PDGF signaling pathway, B cell activation (PANTHER)
CACNA1D	horse	ENSG00000157388	Voltage-sensitive calcium channels (VSCC) mediate the entry of calcium ions into excitable cells and are also involved in a variety of calcium-dependent processes, including muscle contraction, hormone or neurotransmitter release, gene expression, cell motility, cell division and cell death. The isoform alpha-1D gives rise to L-type calcium currents. Long-lasting (L-type) calcium channels belong to the 'high-voltage activated' (HVA) group. They are blocked by dihydropyridines (DHP), phenylalkylamines, benzothiazepines, and by omega-agatoxin-IIIA (omega-Aga-IIIA). They are however insensitive to omega-conotoxin-GVIA (omega-CTx-GVIA) and omega-agatoxin-IVA (omega-Aga-IVA).	Amphetamine addiction, Vascular smooth muscle contraction, MAPK signaling pathway, Calcium signaling pathway, cGMP-PKG signaling pathway, cAMP signaling pathway, Cardiac muscle contraction, Adrenergic signaling in cardiomyocytes, Tight junction, Circadian entrainment, Retrograde endocannabinoid signaling, Glutamatergic synapse, Cholinergic synapse, Serotonergic synapse, GABAergic synapse, Dopaminergic synapse, Insulin secretion, GnRH signaling pathway, Oxytocin signaling pathway, Renin secretion, Aldosterone synthesis and secretion, Type II diabetes mellitus, Carbohydrate digestion and absorption, Alzheimer's disease, Hypertrophic cardiomyopathy, Arrhythmogenic right ventricular cardiomyopathy, Dilated cardiomyopathy (KEGG); Oxytocin receptor mediated signaling pathway, Nicotinic acetylcholine receptor signaling pathway, Beta2 adrenergic receptor signaling pathway, 5HT2 type receptor mediated signaling pathway, Gonadotropin-releasing hormone receptor pathway, Alzheimer disease-amyloid secretase pathway, Beta1 adrenergic receptor signaling pathway (PANTHER)
COA5	dog	ENSG00000183513	Involved in an early step of the mitochondrial complex IV assembly process.	N/A
COL11A1	dog	ENSG00000060718	May play an important role in fibrillogenesis by controlling lateral growth of collagen II fibrils.	Integrin signalling pathway (PANTHER); Extracellular matrix organization (Reactome)
COQ10B	dog	ENSG00000115520	Required for the function of coenzyme Q in the respiratory chain. May serve as a chaperone or may be involved in the transport of Q6 from its site of synthesis to the catalytic sites of the respiratory complexes (By similarity).	Metabolism (Reactome)
DLGAP1	horse	ENSG00000170579	Part of the postsynaptic scaffold in neuronal cells.	Glutamatergic synapse (KEGG)
ERBB4	cattle	ENSG00000178568	Tyrosine-protein kinase that plays an essential role as cell surface receptor for neuregulins and EGF family members and regulates development of the heart, the central nervous system and the mammary gland, gene transcription, cell proliferation, differentiation, migration and apoptosis. Required for normal cardiac muscle differentiation during embryonic development, and for postnatal cardiomyocyte proliferation. Required for normal development of the embryonic central nervous system, especially for normal neural crest cell migration and normal axon guidance. Required for mammary gland differentiation, induction of milk proteins and lactation. Acts as cell-surface receptor for the neuregulins NRG1, NRG2, NRG3 and NRG4 and the EGF family members BTC, EREG and HBEGF. Ligand binding triggers receptor dimerization and autophosphorylation at specific tyrosine residues that then serve as binding sites for scaffold proteins and effectors. Ligand specificity and signaling is modulated by alternative splicing, proteolytic processing, and by the formation of heterodimers with other ERBB family members, thereby creating multiple combinations of intracellular phosphotyrosines that trigger ligand- and context-specific cellular responses. Mediates phosphorylation of SHC1 and activation of the MAP kinases MAPK1/ERK2 and MAPK3/ERK1. Isoform JM-A CYT-1 and isoform JM-B CYT-1 phosphorylate PIK3R1, leading to the activation of phosphatidylinositol 3-kinase and AKT1 and protect cells against apoptosis. Isoform JM-A CYT-1 and isoform JM-B CYT-1 mediate reorganization of the actin cytoskeleton and promote cell migration in response to NRG1. Isoform JM-A CYT-2 and isoform JM-B CYT-2 lack the phosphotyrosine that mediates interaction with PIK3R1, and hence do not phosphorylate PIK3R1, do not protect cells against apoptosis, and do not promote reorganization of the actin cytoskeleton and cell migration. Proteolytic processing of isoform JM-A CYT-1 and isoform JM-A CYT-2 gives rise to the corresponding soluble intracellular domains (4ICD) that translocate to the nucleus, promote nuclear import of STAT5A, activation of STAT5A, mammary epithelium differentiation, cell proliferation and activation of gene expression. The ERBB4 soluble intracellular domains (4ICD) colocalize with STAT5A at the CSN2 promoter to regulate transcription of milk proteins during lactation. The ERBB4 soluble intracellular domains can also translocate to mitochondria and promote apoptosis.	ErbB signaling pathway, Calcium signaling pathway, Endocytosis, Proteoglycans in cancer (KEGG); Alzheimer disease-presenilin pathway, EGF receptor signaling pathway, Cadherin signaling pathway (PANTHER)
FAM172A	cattle, dog	ENSG00000113391	N/A	N/A
GGT7	dog	ENSG00000131067	Cleaves glutathione conjugates.	N/A
GRIA1	cat	ENSG00000155511	Ionotropic glutamate receptor. L-glutamate acts as an excitatory neurotransmitter at many synapses in the central nervous system. Binding of the excitatory neurotransmitter L-glutamate induces a conformation change, leading to the opening of the cation channel, and thereby converts the chemical signal to an electrical impulse. The receptor then desensitizes rapidly and enters a transient inactive state, characterized by the presence of bound agonist. In the presence of CACNG4 or CACNG7 or CACNG8, shows resensitization which is characterized by a delayed accumulation of current flux upon continued application of glutamate.	Amphetamine addiction, Circadian entrainment, Long-term depression, Nicotine addiction, cAMP signaling pathway, Neuroactive ligand-receptor interaction, Long-term potentiation, Retrograde endocannabinoid signaling, Glutamatergic synapse, Dopaminergic synapse, Amyotrophic lateral sclerosis (ALS) (KEGG)
GRIK3	dog, cattle	ENSG00000163873	Receptor for glutamate that functions as ligand-gated ion channel in the central nervous system and plays an important role in excitatory synaptic transmission. L-glutamate acts as an excitatory neurotransmitter at many synapses in the central nervous system. The postsynaptic actions of Glu are mediated by a variety of receptors that are named according to their selective agonists. This receptor binds domoate > kainate >> L-glutamate = quisqualate >> AMPA = NMDA.	Glutamatergic synapse, Neuroactive ligand-receptor interaction (KEGG); Huntington disease (PANTHER)
HSD3B7	cat	ENSG00000099377	The 3-beta-HSD enzymatic system plays a crucial role in the biosynthesis of all classes of hormonal steroids. HSD VII is active against four 7-alpha-hydroxylated sterols. Does not metabolize several different C(19/21) steroids as substrates. Involved in bile acid synthesis (PubMed:11067870). Plays a key role in cell positioning and movement in lymphoid tissues by mediating degradation of 7-alpha,25-dihydroxycholesterol (7-alpha,25-OHC): 7-alpha,25-OHC acts as a ligand for the G protein-coupled receptor GPR183/EBI2, a chemotactic receptor for a number of lymphoid cells.	Primary bile acid biosynthesis, Metabolic pathways (KEGG); Andorgen/estrogene/progesterone biosynthesis (PANTHER)
HSPD1	dog	ENSG00000144381	Implicated in mitochondrial protein import and macromolecular assembly. May facilitate the correct folding of imported proteins. May also prevent misfolding and promote the refolding and proper assembly of unfolded polypeptides generated under stress conditions in the mitochondrial matrix.	Gene Expression, Metabolism of proteins (Reactome)
HSPE1	dog	ENSG00000115541	Eukaryotic CPN10 homolog which is essential for mitochondrial protein biogenesis, together with CPN60. Binds to CPN60 in the presence of Mg-ATP and suppresses the ATPase activity of the latter.	N/A
ITGA9	cat	ENSG00000144668	Integrin alpha-9/beta-1 (ITGA9:ITGB1) is a receptor for VCAM1, cytotactin and osteopontin. It recognizes the sequence A-E-I-D-G-I-E-L in cytotactin.	Cell adhesion molecules, PI3K-Akt signaling, Focal adhesion, ECM-receptor interaction, Regulation of actin cytoskeleton, Hypertrophic cardiomyopathy, Arrhythmogenic right ventricular cardiomyopathy, Dilated cardiomyopathy (KEGG); Integrin signalling pathway, Inflammation mediated by chemokine and cytokine signaling pathway (PANTHER)
LRP1B	cattle	ENSG00000168702	otential cell surface proteins that bind and internalize ligands in the process of receptor-mediated endocytosis.	Alzheimer disease-presenilin pathway (PANTHER)
LYST	dog	ENSG00000143669	May be required for sorting endosomal resident proteins into late multivesicular endosomes by a mechanism involving microtubules.	Tropane, piperidine and pyridine alkaloid biosynthesis (KEGG)
MOB4	dog	ENSG00000115540	May play a role in membrane trafficking, specifically in membrane budding reactions.	N/A
MYLK3	cat	ENSG00000140795	Kinase that phosphorylates MYL2 in vitro. Promotes sarcomere formation in cardiomyocytes and increases cardiomyocyte contractility (By similarity).	Inflammation mediated by chemokine and cytokine signaling pathway, Cytoskeletal regulation by Rho GTPase (PANTHER)
NCOA6	dog	ENSG00000198646	Nuclear receptor coactivator that directly binds nuclear receptors and stimulates the transcriptional activities in a hormone-dependent fashion. Coactivates expression in an agonist- and AF2-dependent manner. Involved in the coactivation of different nuclear receptors, such as for steroids (GR and ERs), retinoids (RARs and RXRs), thyroid hormone (TRs), vitamin D3 (VDR) and prostanoids (PPARs). Probably functions as a general coactivator, rather than just a nuclear receptor coactivator. May also be involved in the coactivation of the NF-kappa-B pathway. May coactivate expression via a remodeling of chromatin and its interaction with histone acetyltransferase proteins.	Circadian Clock, Developmental Biology, Gene Expression, Metabolism, Organelle biogenesis and maintenance (Reactome)
NEK4	cat	ENSG00000114904	Protein kinase that seems to act exclusively upon threonine residues (By similarity). Required for normal entry into proliferative arrest after a limited number of cell divisions, also called replicative senescence. Required for normal cell cycle arrest in response to double-stranded DNA damage.	N/A
NT5DC2	horse	ENSG00000168268	N/A	N/A
NTM	horse	ENSG00000182667	Neural cell adhesion molecule.	cellular process, developmental process (PANTHER)
PLAC8L1	cat, cattle	ENSG00000173261	N/A	N/A
PPAP2A	cat	ENSG00000067113	Broad-specificity phosphohydrolase that dephosphorylates exogenous bioactive glycerolipids and sphingolipids. Catalyzes the conversion of phosphatidic acid (PA) to diacylglycerol (DG). Pivotal regulator of lysophosphatidic acid (LPA) signaling in the cardiovascular system. Major enzyme responsible of dephosphorylating LPA in platelets, which terminates signaling actions of LPA. May control circulating, and possibly also regulate localized, LPA levels resulting from platelet activation. It has little activity towards ceramide-1-phosphate (C-1-P) and sphingosine-1-phosphate (S-1-P). The relative catalytic efficiency is LPA > PA > S-1-P > C-1-P. It's down-regulation may contribute to the development of colon adenocarcinoma.	Metabolism (Reactome)
PPAPDC1B	cat	ENSG00000147535	Displays magnesium-independent phosphatidate phosphatase activity in vitro. Catalyzes the conversion of phosphatidic acid to diacylglycerol. May be a metastatic suppressor for hepatocellular carcinoma.	Immune System (Reactome)
PRR11	cat	ENSG00000068489	Plays a critical role in cell cycle progression.	N/A
PVRL3	cattle	ENSG00000177707	Plays a role in cell-cell adhesion through heterophilic trans-interactions with nectin-like proteins or nectins, such as trans-interaction with NECTIN2 at Sertoli-spermatid junctions. Trans-interaction with PVR induces activation of CDC42 and RAC small G proteins through common signaling molecules such as SRC and RAP1. Also involved in the formation of cell-cell junctions, including adherens junctions and synapses. Induces endocytosis-mediated down-regulation of PVR from the cell surface, resulting in reduction of cell movement and proliferation. Plays a role in the morphology of the ciliary body.	Cell adhesion molecules, Adherens junction (KEGG)
RFTN2	dog	ENSG00000162944	N/A	N/A

RNPC3	cat, dog	ENSG00000185946	Participates in pre-mRNA U12-dependent splicing, performed by the minor spliceosome which removes U12-type introns. U12-type introns comprises less than 1% of all non-coding sequences. Binds to the 3'-stem-loop of m7G-capped U12 snRNA.	Gene Expression (Reactome)
SF3B1	dog	ENSG00000115524	Subunit of the splicing factor SF3B required for 'A' complex assembly formed by the stable binding of U2 snRNP to the branchpoint sequence (BPS) in pre-mRNA. Sequence independent binding of SF3A/SF3B complex upstream of the branch site is essential, it may anchor U2 snRNP to the pre-mRNA. May also be involved in the assembly of the 'E' complex. Belongs also to the minor U12-dependent spliceosome, which is involved in the splicing of rare class of nuclear pre-mRNA intron.	Spliceosome (KEGG)
SKA2	dog	ENSG00000182628	Component of the SKA1 complex, a microtubule-binding subcomplex of the outer kinetochore that is essential for proper chromosome segregation. Required for timely anaphase onset during mitosis, when chromosomes undergo bipolar attachment on spindle microtubules leading to silencing of the spindle checkpoint. The SKA1 complex is a direct component of the kinetochore-microtubule interface and directly associates with microtubules as oligomeric assemblies. The complex facilitates the processive movement of microspheres along a microtubule in a depolymerization-coupled manner. In the complex, it is required for SKA1 localization. Affinity for microtubules is synergistically enhanced in the presence of the ndc-80 complex and may allow the ndc-80 complex to track depolymerizing microtubules.	Cell Cycle, Signal Transduction (Reactome)
SNRPD1	cattle	ENSG00000167088	Core component of the spliceosomal U1, U2, U4 and U5 small nuclear ribonucleoproteins (snRNPs), the building blocks of the spliceosome. Thereby, plays an important role in the splicing of cellular pre-mRNAs. Most spliceosomal snRNPs contain a common set of Sm proteins SNRPB, SNRPD1, SNRPD2, SNRPD3, SNRPE, SNRPF and SNRPG that assemble in a heptameric protein ring on the Sm site of the small nuclear RNA to form the core snRNP. May act as a charged protein scaffold to promote snRNP assembly or strengthen snRNP-snRNP interactions through nonspecific electrostatic contacts with RNA.	Spliceosome, Systemic lupus erythematosus (KEGG)
STAB1	horse	ENSG00000010327	Acts as a scavenger receptor for acetylated low density lipoprotein. Binds to both Gram-positive and Gram-negative bacteria and may play a role in defense against bacterial infection. When inhibited in endothelial tube formation assays, there is a marked decrease in cell-cell interactions, suggesting a role in angiogenesis. Involved in the delivery of newly synthesized CHID1/SI-CLP from the biosynthetic compartment to the endosomal/lysosomal system.	Vesicle-mediated transport (Reactome)
SYTL1	cat	ENSG00000142765	May play a role in vesicle trafficking (By similarity). Binds phosphatidylinositol 3,4,5-trisphosphate. Acts as a RAB27A effector protein and may play a role in cytotoxic granule exocytosis in lymphocytes (By similarity).	Vesicle-mediated transport (Reactome)
TAS2R16	cattle	ENSG00000128519	Gustducin-coupled receptor implicated in the perception of bitter compounds in the oral cavity and the gastrointestinal tract. Signals through PLCB2 and the calcium-regulated cation channel TRPM5.	Taste transduction (KEGG)
TEX14	cat	ENSG00000121101	Required both for the formation of intercellular bridges during meiosis and for kinetochore-microtubule attachment during mitosis. Intercellular bridges are evolutionarily conserved structures that connect differentiating germ cells and are required for spermatogenesis and male fertility. Acts by promoting the conversion of midbodies into intercellular bridges via its interaction with CEP55: interaction with CEP55 inhibits the interaction between CEP55 and PDCD6IP/ALIX and TSG101, blocking cell abscission and leading to transform midbodies into intercellular bridges. Also plays a role during mitosis: recruited to kinetochores by PLK1 during early mitosis and regulates the maturation of the outer kinetochores and microtubule attachment. Has no protein kinase activity in vitro (By similarity).	N/A
TP53BP1	cat	ENSG00000067369	Plays a key role in the response to DNA damage. May have a role in checkpoint signaling during mitosis. Enhances TP53-mediated transcriptional activation.	NOD-like receptor signaling pathway (KEGG)
ZMYND10	cat	ENSG00000004838	Required for motile ciliary function. Probably involved in axonemal assembly of inner and outer dynein arms (IDA and ODA, respectively) for proper axoneme building for cilia motility. May act by indirectly regulating transcription of dynein proteins.	N/A
ZNF521	cattle	ENSG00000198795	Transcription factor that can both act as an activator or a repressor depending on the context. Involved in BMP signaling and in the regulation of the immature compartment of the hematopoietic system. Associates with SMADs in response to BMP2 leading to activate transcription of BMP target genes. Acts as a transcriptional repressor via its interaction with EBF1, a transcription factor involved specification of B-cell lineage; this interaction preventing EBF1 to bind DNA and activate target genes.	N/A
