# Supplementary material for: Self-domestication in Homo sapiens: Insights from comparative genomics
Source: PLoS One. 2017 Oct 18;12(10):e0185306. doi: 10.1371/journal.pone.0185306 (PMC5646786; doi:10.1371/journal.pone.0185306)
Supplement: S3 Table — (PDF) [file pone.0185306.s004.pdf]

S3 Table. Genes overlapping between AMH and two or more domesticated species

GENE NAME	*SPECIES OVERLAP*	*ENSEMBL ID*	*GENE FUNCTION (UniProt)*	*PATHWAY ANNOTATION*	*RELATED DISORDERS*
BRAF	cat, horse, human	ENSG00000157764	Protein kinase involved in the transduction of mitogenic signals from the cell membrane to the nucleus. May play a role in the postsynaptic responses of hippocampal neuron. Phosphorylates MAP2K1, and thereby contributes to the MAP kinase signal transduction pathway.	EGFR tyrosine kinase inhibitor resistance, Endocrine resistance, MAPK signaling pathway, ErbB signaling pathway, Rap1 signaling pathway, cAMP signaling pathway, Chemokine signaling pathway, FoxO signaling pathway, mTOR signaling pathway, Vascular smooth muscle contraction, Dorso-ventral axis formation, Focal adhesion, Natural killer cell mediated cytotoxicity, Long-term potentiation, Neurotrophin signaling pathway, Serotonergic synapse, Long-term depression, Regulation of actin cytoskeleton, Insulin signaling pathway, Progesterone-mediated oocyte maturation, Alcoholism, Hepatitis C, Pathways in cancer, Proteoglycans in cancer, Colorectal cancer, Renal cell carcinoma, Pancreatic cancer, Endometrial cancer, Glioma, Prostate cancer, Thyroid cancer, Melanoma, Bladder cancer, Chronic myeloid leukemia, Acute myeloid leukemia, Non-small cell lung cancer, Breast cancer (KEGG); VEGF signaling pathway, T cell activation, Interleukin signaling pathway, Inflammation mediated by chemokine and cytokine signaling pathway, Integrin signalling pathway, EGF receptor signaling pathway, Ras Pathway, Angiogenesis, CCKR signaling map, PDGF signaling pathway, B cell activation (PANTHER)	Thyroid cancer; Malignant melanoma; Noonan syndrome and related disorders; Langerhans cell histiocytosis (KEGG); Adenocarcinoma of lung, somatic; Cardiofaciocutaneous syndrome; Colorectal cancer, somatic; LEOPARD syndrome; Melanoma, malignant, somatic; Nonsmall cell lung cancer, somatic; Noonan syndrome (OMIM)
FAM172A	cattle, dog, human	ENSG00000113391	N/A	N/A	N/A
GRIK3	dog, cattle, human	ENSG00000163873	Receptor for glutamate that functions as ligand-gated ion channel in the central nervous system and plays an important role in excitatory synaptic transmission. L-glutamate acts as an excitatory neurotransmitter at many synapses in the central nervous system. The postsynaptic actions of Glu are mediated by a variety of receptors that are named according to their selective agonists. This receptor binds domoate > kainate >> L-glutamate = quisqualate >> AMPA = NMDA.	Glutamatergic synapse, Neuroactive ligand-receptor interaction (KEGG); Huntington disease (PANTHER)	schizophrenia; alcohol dependence (MalaCards)
PLAC8L1	cat, cattle, human	ENSG00000173261	N/A	N/A	N/A
RNPC3	cat, dog, human	ENSG00000185946	Participates in pre-mRNA U12-dependent splicing, performed by the minor spliceosome which removes U12-type introns. U12-type introns comprises less than 1% of all non-coding sequences. Binds to the 3'-stem-loop of m7G-capped U12 snRNA.	Gene Expression (Reactome)	growth hormone deficiency, parainfluenza virus type 3, idiopathic inflammatory myopathy, rheumatic disease (MalaCards)
