# Supplementary material for: Self-domestication in Homo sapiens: Insights from comparative genomics
Source: PLoS One. 2017 Oct 18;12(10):e0185306. doi: 10.1371/journal.pone.0185306 (PMC5646786; doi:10.1371/journal.pone.0185306)
Supplement: S4 Table — Gene lists and statistical analysis of data from domesticates, between AMH and each domesticate, and between great apes and domesticates. (PDF) [file pone.0185306.s005.pdf]

S4 Table. (A) Gene lists from the sources for AMH, domesticates, and great apes

AMH POOL				*DOMESTICATES POOL*						*GREAT APE POOL (Cagan et al. 2016)*		
Peyrégne et al. 2016 (SF1, SF2, Table 2 and Table S7) [375]	*Racimo 2016 (Table 3) [326]*	*Prüfer et al. 2014 (Table S19b.1) [108]*	*TOTAL AMH (no duplicates) [742]*	*DOG [229]*	*CAT [291]*	*CATTLE [78]*	*HORSE [109]*	*TOTAL DOMESTICATES (no duplicates) [691]*		*Chimp (P.t. troglodytes) [415]*	*Orango (P. abelii) [500]*	*(Gorilla) G.g. gorilla [426]*
ABCE1	ABCE1	ABCE1	ABCE1	ABAT	ABHD1	ABCC4-like	ABCA10	ABAT	dog	ABLIM2	ABCC6	AADACL3
ABHD3	ABHD14A	ABHD3	ABHD14A	ACA64	ACOT11	ADAM2	ABCA5	ABCA10	horse	ACOT2	ACOX3	ABCA13
ACTG1P4	ABHD14B	AIG1	ABHD14B	ACMSD	ACOT13	ADAMTS13	ABCB10	ABCA5	horse	ACSL6	ACTBP13	ACAN
ADRA2A	ABHD3	AMBRA1	ABHD3	ACSM5	ACOT8	ANK1	ACAD8	ABCB10	horse	ADAM18	ADAM12	ACTBP13
ADSL	ACE	ANAPC10	ACE	ACSS2	ACOX2	ASTN2	ACSF3	ABCC4-like	cattle	ADAM20P3	ADAMDEC1	ACTN3
AGO1	ACTG2	ARHGAP1	ACTG1P4	ADRB2	ACOX3	ATL1	ACTA1	ABHD1	cat	ADAMTSL4	ADAMTS18	ADAM20P3
AGO3	ACY1	ATG13	ACTG2	AHCY	ADAMDEC1	CACNA1C	AKAP1	ACA64	dog	ADGB	ADAMTS8	ADAM24P
AIDA	ADAL	BAG4	ACY1	AHCYL2	ADAMTS13	CAV1	ALDH1L2	ACAD8	horse	ADK	ADAMTSL3	ADAMTSL1
AKAP8	ADRA2B	BCAP29	ADAL	ALDH18A1	ADAMTSL3	CAV2	ALK	ACMSD	dog	ADORA2B	AGL	ADCY8
AKAP8L	ADSL	BEAN1	ADRA2A	ANKS4B	AK1	CDH1	AMBRA1	ACOT11	cat	AGBL4	AGMO	ADRBK1
AKR7A2P1	AHDC1	BIRC2	ADRA2B	APOPT1	ALDH16A1	CLCA3	ANKDD1A	ACOT13	cat	AGGF1P4	AGO4	AF241725.1
AL122050	AHSA2	C11orf80	ADSL	ARID1B	ALS2CR12	COBL	ARL6IP1	ACOT8	cat	AIDA	AK7	AF241725.6
ALG9	ALAS1	C1orf190	AGO1	ARL9	AMACR	CUL1	ASAP1	ACOX2	cat	AKR1C6P	AKAP6	AFM
AMPH	ALMS1	CBL	AGO3	ART3	ANKRD2	DBI	ASTN1	ACOX3	cat	AKR1E2	AKNAD1	AGMO
AMY1A	ANAPC10	CCDC153	AHDC1	ASIP	ANKRD49	DCST1	B3GALTL	ACSF3	horse	ALK	ALDH4A1	ANKRD44
AMY1B	ANKRD30A	CEBPD	AHSA2	ATXN7L1	ANKRD50	DSCAM	BRAF	ACSM5	dog	AMBRA1	ALK	ANO4
AMY1C	ANKRD30B	CHRM4	AIDA	BAG5	APEH	EMC2	C15orf60	ACSS2	dog	AMT	ANKRD17	APP
AMY2A	ANKRD32	CKLF	AIG1	BTAF1	APOBEC4	ERBB4	C17orf67	ACTA1	horse	ANKRD26P3	ANO10	AQP2
AMY2B	ANKRD55	CLASP2	AKAP8	C7orf72	ARHGAP26	EYA1	C4orf33	ADAM2	cattle	APOOP2	ANO5	AQP5
ANAPC10	ARHGAP15	CMTM1	AKAP8L	CADM2	ARID3B	FAM172A	C5orf15	ADAMDEC1	cat	AQP10	ANPEP	ARHGAP27
ANK2	ARID1A	COG5	AKR7A2P1	CALCB	ASB11	FCRL4	C9orf89	ADAMTS13	cat, cattle	ARNT2	AP3S1	ARHGAP39
ANO10	ASTL	COQ10B	AL122050	CBD118	ATXN7L1	GRIK3	CACNA1D	ADAMTSL3	cat	ARPC3	ARHGAP24	ARL4A
ANO3	B2M	CTPS	ALAS1	CBD121	BARD1	HMGA2	CDK5RAP1	ADRB2	dog	ASH1L	ARL2BPP10	ARMC4
ANXA2	BAP1	DDHD2	ALG9	CBD122	BCAP31	HS3ST4	CDKL3	AHCY	dog	ATG13	ARSJ	ARNT
ARSJ	BCAP29	DGKZ	ALMS1	CCDC67	BPI	KDR	CNTN6	AHCYL2	dog	ATG4C	AS1	ATP5J2
ASAP2	BMS1	DNAJA2	AMBRA1	CCDC82	BRAF	KIF6-like	COIL	AK1	cat	ATP6V0E2	ATP2A2	ATRNL1
ASIC2	BZRAP1	DPYSL5	AMPH	CCNJ	BRCA1	KIT	COL22A1	AKAP1	horse	ATP8B2	ATP8A2P3	AUTS2
ATG10	C17orf47	DUS4L	AMY1A	CCNT2	C11orf54	LAMC3	COMMD1	ALDH16A1	cat	ATRNL1	ATRNL1	BAD
ATP1A3	C19orf44	EHBP1	AMY1B	CDRT1	C11orf63	LILRA6	CRTC3	ALDH18A1	dog	AUTS2	ATXN2	BAI3
ATXN10	C1orf172	ESCO1	AMY1C	CDRT4	C16orf71	LIN28B	C-SKI	ALDH1L2	horse	BAI3	AUTS2	BANK1
BAI3	C2orf47	FAM150A	AMY2A	CETN3	C1orf109	LRP1B	DCC	ALK	horse	BCAS2	BARD1	BBS1
BAP1	C2orf69	FLJ35017	AMY2B	CKB	C22orf31	LTF	DLGAP1	ALS2CR12	cat	BCL9	BCAT1	BCAR4
BBIP1	C2orf78	FLJ39294	ANAPC10	CLEC5A	C2orf40	MAFK	DNAH9	AMACR	cat	BSN	BCR	BICC1
BCAP29	C3orf18	GPR22	ANK2	COA5	C2orf62	MAGEA13P-like	EEA1	AMBRA1	horse	BTNL2	BCYRN1	BTBD9
BCAR3	C3orf35	GPT2	ANKRD30A	COG6	C3orf62	MC1R	FAF1	ANK1	cattle	C10orf137	BIVM	C10orf90
BCL2	CACNA1D	GREB1L	ANKRD30B	COL11A1	C8B	MGC12345	FANCA	ANKDD1A	horse	C10orf53	BMP6P1	C11orf68
BIRC2	CACNA2D2	GRID2	ANKRD32	COQ10B	C9orf96	MITF	FBXO31	ANKRD2	cat	C16orf87	BNIP3P1	C11orf80
BRAF	CALR3	HARBI1	ANKRD55	CPEB3	CAGE1	NCAPG	FCHSD2	ANKRD49	cat	C1orf123	BNIPL	C11orf86
BRD4	CAPN3	HBP1	ANO10	CRYM	CASP7	NDUFB1	FOXJ3	ANKRD50	cat	C1orf63	BSN	C12orf35
BROX	CASC4	HIVEP2	ANO3	CUX2	CBX2	NPAS3	GAK	ANKS4B	dog	C21orf59	BTNL2	C16orf52
C11orf1	CATSPER2	HSDL2	ANXA2	CXCL10	CCDC38	NPTX1	GNPTAB	APEH	cat	C2orf78	C10orf115	C16orf58
C16orf87	CBLL1	HSPD1	ARHGAP1	CYFIP1	CCDC64B	NR2F2	GRID1	APOBEC4	cat	C3orf52	C10orf68	C16orf59
C18orf42	CCDC53	HSPE1	ARHGAP15	CYP26A1	CCDC70	NRG2	IGSF3	APOPT1	dog	C3orf62	C12orf60	C1orf63
C1orf112	CCNDBP1	IL7	ARID1A	CYP26C1	CD27	NRG4	IGSF9B	ARHGAP26	cat	C3orf84	C14orf23	C1orf68
C1QTNF5	CCNH	INA	ARSJ	DDC	CD48	NRXN1	JAM3	ARID1B	dog	C7orf61	C16orf95	C2orf78
CACNA2D1	CCNO	INPP5F	ASAP2	DEFB119	CD93	OLIG1	JPH3	ARID3B	cat	C9orf135	C18orf34	C3orf15
CADPS	CCT7	ITFG1	ASIC2	DEFB122	CDH6	OR13C8	KCNK10	ARL6IP1	horse	CACNA2D3	C1DP2	C4BPAP1
CADPS2	CD164L2	LRFN4	ASTL	DNAH3	CELA1	OR51A7	KIAA0556	ARL9	dog	CASP14	C1DP4	C5orf60
CALN1	CDAN1	LRRC41	ATG10	DNAJA1	CENPE	ORF olfactory receptor family cluster (Chromosome 7, BP 15246693)	KIAA1549	ART3	dog	CASQ2	C1orf129	C7orf50
CAMK1G	CDC20B	LSM1	ATG13	DOCK2	CENPM	ORF olfactory receptor family cluster (Chromosome 7, BP 15331252)	KLHDC4	ASAP1	horse	CCDC102B	C1orf132	C8orf82
CAPN5	CDC42EP3	LSR7	ATP1A3	DTD1	CEP68	ORF olfactory receptor family cluster (Chromosome 7, BP 43710839)	LCLAT1	ASB11	cat	CCDC34	C1orf170	CABS1
CAPS	CHERP	MAP7	ATXN10	EIF2S2	CEP97	ORF olfactory receptor family cluster (Chromosome 7, BP 43810382)	LEPREL1	ASIP	dog	CCDC36	C3orf20	CACNA1E
CASP16P	CISH	MCAM	B2M	ELF2	CHMP4B	PCDH18	LMF1	ASTN1	horse	CCDC58P3	C4orf22	CACNA2D2
CBLN4	CKMT1A	MCM4	BAG4	ENSCAFG00000018988	CIB4	PEX7	MAP3K4	ASTN2	cattle	CCDC69	C6orf10	CAMKV
CCDC188	CKMT1B	MCMBP	BAI3	ENSCAFG00000023289	CLDN17	PLAC8L1	MAPK10	ATL1	cattle	CCDC71	C7orf57	CBFB
CCDC192	CLDN10	MDK	BAP1	ENTPD1	CLEC5A	PLAG1	MARCH10	ATXN7L1	dog, cat	CCDC91	C8orf56	CCDC129
CCNJL	CNGA3	MEGF10	BBIP1	EPS15	CLK3	Pol	MATN2	B3GALTL	horse	CDH12	C9orf11	CCDC146
CDC27	COA5	MIB1	BCAP29	ETNPPL	CNGA2	PVRL3	MSI2	BAG5	dog	CDHR4	C9orf153	CCDC85B
CDH10	COG5	MKLN1	BCAR3	F9	COL6A3	RNF144B	MYBPC1	BARD1	cat	CDK8	C9orf3	CCDC87
CEBPD	CSGALNACT2	MOB4	BCL2	FABP5	COL9A3	ROBO1	NCAPD3	BCAP31	cat	CDKAL1	CAND2	CCNF
CELF4	CTDSPL2	NCOA5	BEAN1	FAM107B	CROCC	SEMA6A	NID2	BPI	cat	CENPQ	CAPRIN2	CCS
CEP41	CYB561	NETO2	BIRC2	FAM172A	CSPP1	SETMAR	NINJ1	BRAF	cat, horse	CGNL1	CAPZB	CD1B
CEP57L1	CYB561D2	NRG3	BMS1	FAM40B	CTTN	SLC35D1	NIPBL	BRCA1	cat	CHAT	CARD16	CD2BP2
CHODL	DDX4	NLK	BRAF	FBXO10	CYB5R1	SLC41A2	NR3C2	BTAF1	dog	CHRM4	CATSPERG	CD300LB
CLSTN1	DGUOK	NLRX1	BRD4	FBXW10	CYP1A1	SLC6A1	NT5DC2	C11orf54	cat	CLC	CCDC15	CD320

AMH POOL				DOMESTICATES POOL						GREAT APE POOL (Cagan et al. 2016)		
Peyrégne et al. 2016 (SF1, SF2, Table 2 and Table S7) [375]	***Racimo 2016 (Table 3) [326]***	***Prüfer et al. 2014 (Table S19b.1) [108]***	***TOTAL AMH (no duplicates) [742]***	***DOG [229]***	***CAT [291]***	***CATTLE [78]***	***HORSE [109]***	***TOTAL DOMESTICATES (no duplicates) [691]***		**Chimp (*P.t. troglodytes*) [415]**	**Orango (*P. abelii*) [500]**	**(Gorilla) *G.g. gorilla* [426]**
CNTNAP4	DHDDS	NUFIP1	BROX	FBXW11	CYP1A2	SNRPD1	NTM	C11orf63	cat	CLN8	CCDC174	CDH13
COG5	DHX29	OTUD4	BZRAP1	FGD6	CYP27B1	SPTAN1	NUMB	C15orf60	horse	CNTNAP4	CCT7P2	CDH9
COL11A1	DNAH1	OTX1	C11orf1	FGF13	DACT1	STK10	NUP133	C16orf71	cat	COL11A2P1	CD163	CDK19
CORO2B	DNAJC3	PC	C11orf80	FGF18	DAPK1	TAOK1	OPCML	C17orf67	horse	CPNE4	CD163L1	CDK20
CR2	DOCK3	PCDH17	C16orf87	FGFBP3	DCC	TAS2R16	PCSK5	C1orf109	cat	CPSF3L	CD1A	CENPQ
CSMD2	DRAM1	PCGF6	C17orf47	FHL1	DNAJB9	TFCP2L1	PDE4DIP	C22orf31	cat	CPT2	CD46	CENPT
CSPG5	DUS4L	PDZD3	C18orf42	FOXI1	DNTTIP2	TMEM132D	PDE5A	C2orf40	cat	CTCF	CD5L	CERS5
CTNNBL1	DUSP11	PHKB	C19orf44	FRMD6	DPEP3	TNFRSF9	PDRG1	C2orf62	cat	CTRL	CDC27	CHIAP1
CTNND2	DUSP7	PODXL	C1orf112	FRMPD1	DUSP19	ULBP3	PHF2	C3orf62	cat	CTTNBP2NL	CDC42BPA	CHIAP2
CTXN3	DZIP1	POMGNT1	C1orf172	GALR1	ECHDC1	UNC93A	PHF20	C4orf33	horse	CXCL13	CDH12	CHTF18
CXCL13	EGR4	PPAPDC1B	C1orf190	GAPDHS	EDC3	VWDE	PIK3C3	C5orf15	horse	CXXC11	CDH17	CICP17
CXCL3	EHBP1	PRDM2	C1QTNF5	GGT7	EHBP1L1	XPO6	PLXNA4	C7orf72	dog	CYP21A2	CDH19	CLEC18A
DAPP1	EIF3J	PRKDC	C2orf47	GLRA1	ENKUR	ZNF521	POP1	C8B	cat	DAG1	CDK8	CLSTN3
DBIL5P2	ELL3	QSER1	C2orf69	GNG10	ENTPD7		PPM1D	C9orf89	horse	DAP3P1	CECR7	CNIH2
DGCR8	EPB42	RAD54L	C2orf78	GNG4	EPHB4		PPP2CA	C9orf96	cat	DCP2	CETN1	CNTN4
DHRS12	EPM2AIP1	RB1CC1	C3orf18	GP2	ETV4		PRKCZ	CACNA1C	cattle	DENND2C	CHCHD6	CNTNAP5
DLGAP1	EPS15L1	RBM14	C3orf35	GPR139	FAIM3		PRMT3	CACNA1D	horse	DGKZ	CHRNA6	COL14A1
DNAH1	ESCO1	RBM4	CACNA1D	GPRC5B	FAM114A2		PSMB7	CADM2	dog	DHFRP2	CHST11	COL19A1
DNAJB4	ESM1	RBM4B	CACNA2D1	GRIK3	FAM131B		PTPN4	CAGE1	cat	DHX57	CHST9	COL24A1
DTNA	FAHD2A	RCE1	CACNA2D2	HERC2	FAM179A		RHPN1	CALCB	dog	DIAPH3	CLCC1	COX14
DUS4L	FAM172A	RFTN2	CADPS	HIPK2	FAM69A		SCPEP1	CASP7	cat	DIO2	CLEC2D	CPNE7
DYNC1H1	FAM46B	RSPO3	CADPS2	HOPX	FANCB		SEC24A	CAV1	cattle	DISC1FP1	CLECL1	CPSF4
DYSF	FAM83F	SCMH1	CALN1	HSPD1	FAT4		SEC63	CAV2	cattle	DISP1	CLUL1	CRP
E2F6	FBXO41	SEC23IP	CALR3	HSPE1	FBN3		SGCD	CBD118	dog	DLG1	CNBD1	CRPP1
EFCC1	FCN3	SEC24D	CAMK1G	HTR4	FBXL22		SKP1	CBD121	dog	DLGAP4	CNGB3	CTDSPL
EHBP1	FRMD5	SF3B1	CAPN3	IFT80	FBXO28		SLC22A15	CBD122	dog	DMRTA2	CNTN1	CTNNA3
ELAVL4	FXYD4	SLC12A5	CAPN5	IKZF1	FER		SLC43A1	CBX2	cat	DNAI2	COBLL1	CTSF
ELN	GALNT11	SLFNL1	CAPS	IMMP2L	FGA		SMG6	CCDC38	cat	DNASE1L3	COL11A2P1	CTSK
ELP6	GALNTL5	SLITRK3	CASC4	JRKL	FN3K		SPATA19	CCDC64B	cat	DOCK3	COL5A2	CUX2
ENTHD1	GANC	SNAI2	CASP16P	KDM3A	FRMD7		STAB1	CCDC67	dog	DOK5	COL8A1	CYB561D2
EPSTI1	GATA6	SNRPD1	CATSPER2	LHFPL3	GCNT7		STXBP6	CCDC70	cat	DPYS	CPD	CYB5P4
ERBB4	GDPD1	SPTBN2	CBL	LINC01927	GEMIN7		SUSD3	CCDC82	dog	DUSP11	CPEB4	DAOA
ESCO1	GLT8D1	STAC	CBLL1	LINGO2	GGT6		TCTN1	CCNJ	dog	DVL1	CPNE3	DCTPP1
EXOC6B	GLYCTK	STK3	CBLN4	LRIG3	GOLGA1		THYN1	CCNT2	dog	EEF1A1P14	CPNE8	DDN
EXTL1	GNAI2	TAF5	CCDC153	LRRN3	GPATCH8		TRAPPC8	CD27	cat	EHBP1L1	CR1	DDR2
FAAH	GNAT1	TDRD3	CCDC188	LYST	GPR133		TRIO	CD48	cat	EMCN	CR2	DDX39BP2
FAF2	GNL3	TK2	CCDC192	MAOA	GPR15		UBE2B	CD93	cat	ENOX1	CRISP3	DDX55
FAM117A	GOLGA4	UBE2V2	CCDC53	MAOB	GPR174		URB2	CDH1	cattle	EPB41L4A	CSMD1	DENND6A
FAM177B	GPAT2	UQCRH	CCNDBP1	MAP3K1	GPRASP2		VDAC1	CDH6	cat	ESRG	CSMD3	DHFRP2
FAM19A3	GPATCH3	UQCRHL	CCNH	MAP7D3	GPRC5A		VPS26B	CDK5RAP1	horse	EXOC4	CTNNAL1	DLG2
FAM49B	GPN2	WHSC1L1	CCNJL	MARCH7	GPRIN2		VRK1	CDKL3	horse	FAF1	CYSTM1	DMXL2
FAU	GPR22	ZNF638	CCNO	MARK2	GRHL3		WASF3	CDRT1	dog	FAM117B	DACH1	DNAH9
FBXL19	GPR3	ZNF852	CCT7	MARK3	GRIA1		WNK2	CDRT4	dog	FAM150B	DAG1	DPP3
FBXW7	GPR39		CD164L2	MBD2	GRIA2		ZC3H3	CELA1	cat	FAM162B	DCAF10	DRAP1
FDXACB1	GPR62		CDAN1	MBP	HADH			CENPE	cat	FAM83G	DCHS2	DSG4
FERMT2	GPX8		CDC20B	MCF2	HEATR5B			CENPM	cat	FAM89B	DDAH1	DUSP11
FGF12	GRAP2		CDC27	MCHR2	HECA			CEP68	cat	FAM98B	DDX31	DYNC2H1
FGF14	GREB1L		CDC42EP3	MED23	HEPACAM2			CEP97	cat	FBN2	DDX39BP1	EAF1
FHL3	GRM2		CDH10	METAP2	HEPH			CETN3	dog	FBXW2	DDX39BP2	EDNRA
FHOD3	GTDC1		CEBPD	METTL22	HMMR			CHMP4B	cat	FBXW7	DDX42	EFCAB4B
FIBCD1	GYPA		CELF4	MGAM	HPS5			CIB4	cat	FCF1P6	DEFA1B	EFTUD1P2
FKSG51	GYPB		CEP41	MIER3	HSD3B7			CKB	dog	FCGBP	DENND5A	EGFLAM
FLJ45513	GZMA		CEP57L1	MIF4GD	HSPA13			CLCA3	cattle	FER1L5	DENND6B	EIF2B1
FNBP1L	GZMK		CHERP	MINOS1	IFT81			CLDN17	cat	FGFR3	DFNB59	EIF4BP8
FOXO1	HAUS2		CHODL	MKKS	IGHMBP2			CLEC5A	dog, cat	FGFR3P1	DHFRP2	EME1
FRMD8	HBP1		CHRM4	MMP16	INHBC			CLK3	cat	GABRB1	DIP2B	EML5
FSTL5	HEMK1		CISH	MOB4	INPP4B			CNGA2	cat	GALNT14	DLEU2	EVPL
FUBP1	HHIP		CKLF	MPV17L	INPP5J			CNTN6	horse	GALNTL6	DLG2	EYS
FUT5	HMGN2		CKMT1A	MT1F	IPO4			COA5	dog	GAPDHP23	DNAH12	FAM46A
FZD3	HNRNPF		CKMT1B	MT1L	IQCB1			COBL	cattle	GAPDHP70	DNAJB14	FASTKD3
GABRB3	HS6ST3		CLASP2	MT2A	ISG15			COG6	dog	GFOD2	DNAJB9	FBN2
GALNT10	HSF5		CLDN10	MYOF	ITGA2B			COIL	horse	GFRA2	DNAJC13	FBXL17
GALNT2	HYAL1		CLSTN1	NCOA6	ITGA9			COL11A1	dog	GINS3	DOC2B	FER
GBP2	HYAL2		CMTM1	NIPA2	ITGBL1			COL22A1	horse	GLTPD1	DOK6	FER1L6
GBP4	HYAL3		CNGA3	NKAIN2	ITPR3			COL6A3	cat	GLYATL1	DYNLRB2	FGFR3P1
GBP5	IFRD2		CNTNAP4	NOCT	JMJD1C			COL9A3	cat	GLYATL1P2	EEFSEC	FHIT
GBP7	IGF1		COA5	NOL4	KIAA0226			COMMD1	horse	GLYATL2	EFCAB5	FKBP2
GCNT2	IL31RA		COG5	NRF1	KIF1C			COQ10B	dog	GM2A	EHBP1	FNDC3B
GDAP1	IL6ST		COL11A1	NRG2	KIF22			CPEB3	dog	GNAQ	EIF4A3	FOSL1
GDF6	INPP4A		COQ10B	NTAN1	KIF27			CROCC	cat	GPN3	ELAC2	FSTL4
GGT7	IQCF1		CORO2B	NUP54	KIRREL2			CRTC3	horse	GPRC6A	EMID1	FTLP6
GINM1	IQCF2		CR2	NXPE3	KRIT1			CRYM	dog	GPX1	EMR3	GAL3ST3
GK2	IQCF3		CSGALNACT2	OMA1	KYNU			C-SKI	horse	GRAP	EPB41	GALC
GLI3	IQCF5		CSMD2	OR9A4	LAMC2			CSPP1	cat	GRIK1	EPHA3	GALNT13
GLYCTK	IQCF6		CSPG5	PARP12	LAP3			CTTN	cat	GRIN3A	ERCC5	GALNT14
GP9	ITGA9		CTDSPL2	PDE4D	LATS2			CUL1	cattle	GSTP1	ERG	GAPDHP36

AMH POOL				DOMESTICATES POOL						GREAT APE POOL (Cagan et al. 2016)		
Peyrégne et al. 2016 (SF1, SF2, Table 2 and Table S7) [375]	*Racimo 2016 (Table 3) [326]*	*Prüfer et al. 2014 (Table S19b.1) [108]*	*TOTAL AMH (no duplicates) [742]*	*DOG [229]*	*CAT [291]*	*CATTLE [78]*	*HORSE [109]*	*TOTAL DOMESTICATES (no duplicates) [691]*		*Chimp (P.t. troglodytes) [415]*	*Orango (P. abelii) [500]*	*(Gorilla) G.g. gorilla [426]*
GPM6A	ITIH1		CTNNBL1	PDE7B	LCAT			CUX2	dog	HADH	EWSR1	GAS2L3
GPR22	ITIH3		CTNND2	PDILT	LIAS			CXCL10	dog	HCG23	EXOC4	GBP1
GPT2	ITIH4		CTPS	PDXDC1	LIMD1			CYB5R1	cat	HCG4	EXOSC3	GNG13
GRIA1	KCNIP3		CTXN3	PKD1L1	LRRC32			CYFIP1	dog	HCG4P11	EYS	GOLPH3L
GRIK3	KCNJ3		CXCL13	PLCE1	LRRC36			CYP1A1	cat	HCG9P5	FAM172A	GPD1
GRIK5	KIAA0825		CXCL3	PLEKHM3	LSM3			CYP1A2	cat	HDAC9	FAM190A	GPR137
GRM3	KIAA1841		CYB561	POLI	MAP7D2			CYP26A1	dog	HEATR1	FAM5C	GRIA4
GTF3C5	KLF2		CYB561D2	POLR1E	MARVELD3			CYP26C1	dog	HEATR4	FAM71A	GRID2
HEG1	KMT2C		DAPP1	Q2ABD2	MERTK			CYP27B1	cat	HEMK1	FAN1	GRIK2
HERC5	LCMT2		DBIL5P2	RAB3GAP1	METTL8			DACT1	cat	HIST1H2BK	FASTKD1	GSK3B
HHIP	LGALSL		DDHD2	RABGAP1L	MFAP3			DAPK1	cat	HIVEP3	FBLN2	GSPT1
HIVEP2	LIN28A		DDX4	RALY	MIIP			DBI	cattle	HLA-B	FBXW4P1	GTF2H3
HMGB3P1	LRRC57		DGCR8	RANBP17	MORC1			DCC	cat, horse	HLA-C	FCHO2	GTF2IP3
HRASLS2	LRRFIP2		DGKZ	RAPH1	MRPL11			DCST1	cattle	HLA-DPA1	FCRL1	HARS2
HS6ST3	LSMEM2		DGUOK	REEP1	MRPL52			DDC	dog	HLA-DPA2	FGD5P1	HBE1
HSD3B7	MAL		DHDDS	RELT	MTIF2			DEFB119	dog	HLA-DPB1	FGF7P1	HCG4P3
HSDL2	MANF		DHRS12	RFTN2	MTRF1			DEFB122	dog	HLA-DPB2	FGFR3P1	HCG4P5
HTR1E	MAP1A		DHX29	RG9MTD3	MURC			DLGAP1	horse	HLA-DQA1	FKBP6	HCG9
IGFL2	MAP3K6		DLGAP1	RNF103	MVK			DNAH3	dog	HLA-DQB1	FKBP7	HCP5
IGFL3	MAPKAPK3		DNAH1	RNPC3	MYLK3			DNAH9	horse	HLA-DRB1	FLJ39534	HDGFRP3
IGFL4	MCHR1		DNAJA2	RPL3	MYO15A			DNAJA1	dog	HLA-E	FNBP4	HECW1
IL7	MCIDAS		DNAJB4	RPL31	MYO9A			DNAJB9	cat	HLA-F	FTO	HECW1-IT1
INPP5F	MCTP1		DNAJC3	RRN3	NAPRT1			DNTTIP2	cat	HLA-V	FTSJ3	HERC5
JMJD6	MDH1		DOCK3	RRN3P1	NEK1			DOCK2	dog	HMCN1	FYB	HLA-A
KAT7	MED26		DPYSL5	RRNRP2	NEK4			DPEP3	cat	HMGA1P3	GAA	HLA-C
KATNA1	MFAP1		DRAM1	SAE1	NFAM1			DSCAM	cattle	HNRNPA1P60	GABRB1	HLA-DPA1
KCNA4	MGAT4A		DTNA	SCARB2	NFKBIZ			DTD1	dog	HSD3BP1	GABRP	HLA-DPB1
KCND2	MIB1		DUS4L	SCP2D1	NOLC1			DUSP19	cat	HSD3BP2	GAS2L1	HLA-DQA1
KCNH7	MKL1		DUSP11	SDAD1	NOSTRIN			ECHDC1	cat	HSFY1P1	GBE1	HLA-DQA2
KCNJ3	MLH1		DUSP7	SEMA3D	NOTCH2			EDC3	cat	HVCN1	GCSAML	HLA-DQB1
KIAA1143	MRPS5		DYNC1H1	SETBP1	NPFFR2			EEA1	horse	ICA1L	GDPD4	HLA-DQB2
KIAA1958	MTMR4		DYSF	SETD9	NRG2			EHBP1L1	cat	IFITM4P	GHR	HLA-DQB3
KIF15	MTRNR2L7		DZIP1	SF3B1	NUDT15			EIF2S2	dog	IGKV1D-17	GIMAP7	HLA-DRA
KIF18A	MUSTN1		E2F6	SH3GL2	OPTC			ELF2	dog	IGKV6D-41	GLYATL2	HLA-DRB1
KIFAP3	NAT6		EFCC1	SKA2	OR10K1			EMC2	cattle	IGLVIV-64	GPR113	HLA-DRB9
KLHL18	NAT8		EGR4	SLC5A1	OR2B11			ENKUR	cat	IGLVIV-65	GPSM2	HLA-J
LARGE1	NEDD1		EHBP1	SLC5A4	OR4D6			ENSCAFG00000018988	dog	IGLVIV-66-1	GTF2E1	HLA-S
LDHD	NEK4		EIF3J	SLC6A17	OTOF			ENSCAFG00000023289	dog	IGLVV-66	GTF3C4	HLA-W
LEMD3	NISCH		ELAVL4	SLC9A6	PAFAH2			ENTPD1	dog	IL26	H2AFZ	HMCN2
LIMK1	NOTO		ELL3	SMC4	PARVG			ENTPD7	cat	INPP4B	HCG23	HNRNPH1
LOH11CR1I	NPRL2		ELN	SMIM23	PCDHA1			EPHB4	cat	IP6K1	HCG4B	HORMAD1
LPHN3	NR0B2		ELP6	SMO	PCDHB4			EPS15	dog	KCNH7	HCG4P3	HSBP1P2
LRIG2	NR2F1		ENTHD1	SMYD2	PHLDB3			ERBB4	cattle	KCNIP4	HCG4P7	HSD17B7
LRP1B	NT5DC2		EPB42	SNAP29	PITRM1			ETNPPL	dog	KCNK7	HCG4P8	HYAL1
LYST	NUDC		EPM2AIP1	SRP72	PJA2			ETV4	cat	KCTD7	HCG9	HYAL2
MAGI2	NUP37		EPS15L1	STARD6	PLA2G2E			EYA1	cattle	KHDRBS2	HCP5	HYAL3
MAL	NWD1		EPSTI1	STK10	PLA2G3			F9	dog	KIAA0146	HDGFRP3	IGF2R
MAP2	OTUD4		ERBB4	STX7	PLAC1			FABP5	dog	KIAA1217	HES4	IGLV2-33
MATR3	OTX1		ESCO1	TAS2R38	PLAC8L1			FAF1	horse	KIF19	HHLA2	IGLV3-31
MCM4	PARP3		ESM1	TBC1D9	PLEKHH1			FAIM3	cat	KLHDC8B	HLA-DPA1	IGLV3-32
MCMBP	PARPBP		EXOC6B	TBXAS1	PLIN3			FAM107B	dog	KLHL7	HLA-DPA2	IL36B
MDM1	PATL2		EXTL1	TCTN3	PML			FAM114A2	cat	KLHL8	HLA-DPA3	IMMP2L
METTL15	PBRM1		FAAH	TEKT3	PPAP2A			FAM131B	cat	KRT8P26	HLA-DPB1	IMMTP1
METTL23	PCBP4		FAF2	TH	PPAPDC1B			FAM172A	dog, cattle	KRTAP5-10	HLA-DPB2	INTU
MFRP	PCNX		FAHD2A	THEGL	PPFIBP1			FAM179A	cat	KRTAP5-9	HLA-DQA1	IQCF6
MFSD11	PDE4B		FAM117A	TLX3	PPP1R13B			FAM40B	dog	LCAT	HLA-DQB1	IQGAP2
MIB1	PDIA3		FAM150A	TMEM114	PRICKLE4			FAM69A	cat	LDB2	HLA-DRA	IQUB
MIGA1	PELI1		FAM172A	TMEM132D	PRKAG1			FANCA	horse	LETM1	HLA-DRB1	ITGAL
MMP9	PEX13		FAM177B	TMEM159	PRKG2			FANCB	cat	LGALS8	HLA-DRB9	KCNH8
MPND	PHF7		FAM19A3	TMEM242	PRR11			FAT4	cat	LGR4	HLA-G	KDM2A
MRPL49	PIGV		FAM46B	TNKS2	PRX			FBN3	cat	LHFPL3	HLA-H	KDM4C
MRPS5	PIK3CG		FAM49B	TRBV25OR9-2	PSPH			FBXL22	cat	LIMCH1	HLA-J	KIAA1644
MYH3	PLA2G4D		FAM83F	TRIM16	PSTK			FBXO10	dog	LL22NC03-23C6.15	HLA-K	KLC2
MYHAS	PLA2G4E		FAU	TRIM59	PTPRR			FBXO28	cat	LNPEP	HLA-S	KMT2D
MYL4	PLA2GDF		FBXL19	TRMT61A	PTPRS			FBXO31	horse	LOC124685	HLA-T	KPRP
MYLK3	PMCH		FBXO41	TRY1	PUSL1			FBXW10	dog	LOC644100	HLA-U	KRTAP12-1
NADK2	POC1A		FBXW7	TRY2	RABL3			FBXW11	dog	LPHN3	HLA-W	KRTAP12-2
NAV3	POTEC		FCN3	TRY3	RBM11			FCHSD2	horse	LRBA	HMG20A	KRTAP22-1
NCOA6	POU5F2		FDXACB1	TTC39A	RBP5			FCRL4	cattle	LRP5	HNRNPA2B1	KRTAP22-2
NDUFA11	PPAP2A		FERMT2	TUBGCP5	RCSD1			FER	cat	LRWD1	HSFY1P1	KRTAP6-2
NEAT1	PPIP5K1		FGF12	TVP23B	RELL1			FGA	cat	LTBP3	HTATSF1P2	KRTAP6-3
NEB	PPM1E		FGF14	TVP23C	RHBDD1			FGD6	dog	MACROD2	HTN1	KRTAP9-1
NEXN	PPM1M		FHL3	U2	RIMKLA			FGF13	dog	MANF	HTN3	KRTAP9-12P
NMUR2	PRADC1		FHOD3	UMOD	RNASE6			FGF18	dog	MAP3K11	HTR3E	LCE1E
NTM	PRKAR2B		FIBCD1	V1R	RNPC3			FGFBP3	dog	MCC	IFFO2	LCE1F
NTRK2	PRKCD		FKSG51	VEZT	RSL1D1			FHL1	dog	MCL1	IFI16	LCE4A

AMH POOL				*DOMESTICATES POOL*						*GREAT APE POOL (Cagan et al. 2016)*		
Peyrégne et al. 2016 (SF1, SF2, Table 2 and Table S7) [375]	***Racimo 2016 (Table 3) [326]***	***Prüfer et al. 2014 (Table S19b.1) [108]***	***TOTAL AMH (no duplicates) [742]***	***DOG [229]***	***CAT [291]***	***CATTLE [78]***	***HORSE [109]***	***TOTAL DOMESTICATES (no duplicates) [691]***		**Chimp (*P.t. troglodytes*) [415]**	**Orango (*P. abelii*) [500]**	**(Gorilla) *G.g. gorilla* [426]**
NWD2	PROM2		FLJ35017	VWC2	RTP3			FN3K	cat	MDGA2	IFT52	LDHAL6EP
NXPH1	PRR11		FLJ39294	XPBP	S100A12			FOXI1	dog	MDK	IGFL1P1	LEF1
NYAP2	PUS10		FLJ45513	YSK4	SCN9A			FOXJ3	horse	MEI4	IGLC5	LGALSL
ORAI3	PVRL3		FNBP1L	YWHAH	SCRIB			FRMD6	dog	MEPCE	IGLC6	LIPJ
OTUD4	RAB11FIP5		FOXO1	ZNF236	SDK2			FRMD7	cat	MGAM	IGLC7	LMBR1L
OTX1	RAB28		FRMD5	ZNF286A	SEC24A			FRMPD1	dog	MICC	IGLJ5	LMTK2
PACSIN1	RAD51C		FRMD8	ZNF286B	SENP5			GAK	horse	MICE	IGLJ6	LPHN3
PAIP2	RAD54L2		FSTL5	ZNF492	SENP7			GALR1	dog	MIPEP	IGLJ7	LRRC66
PCCB	RASA1		FUBP1	ZNF516	SEPT10			GAPDHS	dog	MIPEPP1	IGLV4-60	LRWD1
PCDH9	RASGEF1A		FUT5	ZNF679	SERINC3			GCNT7	cat	MKNK2P1	IGLVIV-59	LST3
PDCD4	RASSF1		FXYD4	ZP2	SH2D5			GEMIN7	cat	MMP21	IGLVV-58	LUZP2
PDZD2	RBM15B		FZD3	ZPBP	SHC4			GGT6	cat	MRO	IP6K2	MAK
PHACTR1	REL		GABRB3		SIAE			GGT7	dog	MROH8	IPO11	MAP4
PHF7	RET		GALNT10		SLC22A13			GLRA1	dog	MRPL3P1	IQSEC1	MBD3L2
PLA2G16	RFT1		GALNT11		SLC22A18			GNG10	dog	MRPS5	ISG15	MBD3L3
PLAC8L1	RGS6		GALNT2		SLC25A38			GNG4	dog	MSH4	ITFG1	MBD3L4
PLXDC2	RNF43		GALNTL5		SLC35F5			GNPTAB	horse	MTHFD2P1	KANK1	MCCD1P2
POU2F2	ROCK1		GANC		SLC39A7			GOLGA1	cat	MTND1P3	KANSL2	MEGF11
POU3F1	RPL29		GATA6		SLC39A8			GP2	dog	MUC13	KCNK3	METTL6
PPAPDC1A	RPS6KA1		GBP2		SLC46A1			GPATCH8	cat	MUC16	KCNN2	MICA
PPIL4	RRP9		GBP4		SLCO1A2			GPR133	cat	MUT	KCTD7	MICD
PPM1M	SEMA3F		GBP5		SMG1			GPR139	dog	MYL6P4	KDM3A	MLKL
PPP2R1B	SEMA3G		GBP7		SMG6			GPR15	cat	MYO3B	KIAA1009	MRPL11
PRDM10	SEPT4		GCNT2		SPATA21			GPR174	cat	NCOR1	KIAA1430	MSH4
PRDM2	SERF2		GDAP1		SPATA7			GPRASP2	cat	NDFIP1	KIAA1731	MSLNL
PRKDC	SERINC4HYPK		GDF6		SPERT			GPRC5A	cat	NEK5	KIF13B	MTCO3P1
PROM2	SFMBT1		GDPD1		SPHKAP			GPRC5B	dog	NFIX	KLHL1	MTND1P19
PSTPIP2	SFXN5		GGT7		SPINT1			GPRIN2	cat	NICN1	KNTC1	MTND4P1
PTPN23	SGSM3		GINM1		SPTBN5			GRHL3	cat	NIPBL	KRT79	MTND5P3
PTPRD	SIN3B		GK2		SREBF1			GRIA1	cat	NREP	KRTAP5-10	MTRR
RABAC1	SIPA1L1		GLI3		SRRM2			GRIA2	cat	NRXN3	KRTAP5-9	MUC21
RANBP1	SKA2		GLT8D1		STARD5			GRID1	horse	NT5CP1	KRTCAP3	MUT
RANBP3	SKIV2L2		GLYCTK		STK11IP			GRIK3	dog, cattle	NUS1P4	KYNU	MX1
RARRES3	SLC25A17		GNAI2		STS			HADH	cat	NXPE3	LAMC1	MYH14
RASSF3	SLC26A3		GNAT1		SUN3			HEATR5B	cat	NYAP1	LDHAL6A	NABP1
RBFOX2	SLC26A4		GNL3		SURF2			HECA	cat	OR10C1	LGALS4	NAT6
RBL1	SLC35E1		GOLGA4		SYNM			HEPACAM2	cat	OR11A1	LIMD2	NBPF10
RBSG3	SLC38A9		GP9		SYTL1			HEPH	cat	OR1I1	LIPM	NBPF22P
RIF1	SLC9A1		GPAT2		TAS2R1			HERC2	dog	OR2H1	LIPN	NDUFA5
RNF133	SMAD1		GPATCH3		TAS2R3			HIPK2	dog	OR2W3	LRBA	NDUFA7
RNF148	SMG8		GPM6A		TEX14			HMGA2	cattle	OR4K2	MAP2	NEGR1
RNF220	SMIM4		GPN2		TF			HMMR	cat	OR4K4P	MAP3K3	NLGN1
RNF26	SMIM7		GPR22		THBS2			HOPX	dog	OR5AC2	MAPKAPK5	NPCDR1
RNF44	SMYD5		GPR3		THUMPD1			HPS5	cat	OR5AC4P	MAPKAPK5P1	NPRL2
RNPC3	SNAP23		GPR39		TMEM59L			HS3ST4	cattle	OR5P2	MCCD1P1	NR1I2
ROBO2	SNF		GPR62		TMEM71			HSD3B7	cat	OR5P3	MCCD1P2	NRXN3
RPL13AP6	SNRPD1		GPT2		TOE1			HSPA13	cat	OR5V1	MCPH1	NTN3
RPS18P9	SPATS2L		GPX8		TP53BP1			HSPD1	dog	OXR1	METTL7B	NUTF2
SAMHD1	SPCS1		GRAP2		TRPV6			HSPE1	dog	P4HTM	MICA	OLAH
SCAP	SPG11		GREB1L		TSTD2			HTR4	dog	PAH	MICD	OPCML
SCYL3	STAB1		GRIA1		TXN2			IFT80	dog	PC	MICF	OR10A2
SEC23IP	STAMBP		GRID2		TXNRD2			IFT81	cat	PCDH7	MKI67IPP3	OR10A4
SEC24D	STARD9		GRIK3		TYK2			IGHMBP2	cat	PCNP	MKRN2	OR10A5
SEMA3G	STRC		GRIK5		UBXN10			IGSF3	horse	PCNXL3	MRPL32	OR10J5
SEMA6D	SUPT4H1		GRM2		USP45			IGSF9B	horse	PDZRN4	MRPS21P2	OR2D2
SESN1	SYTL1		GRM3		UVRAG			IKZF1	dog	PGLYRP3	MRPS5	OR2L1P
SETD1A	TANC2		GTDC1		VEZT			IMMP2L	dog	PGLYRP4	MST1R	OR2L6P
SF3A3	TEX14		GTF3C5		WDR17			INHBC	cat	PHBP12	MTCO3P1	OR4K2
SGMS2	TEX264		GYPA		WDR62			INPP4B	cat	PHF14	MTL5	OR51AB1P
SGSM3	TGM5		GYPB		WDR90			INPP5J	cat	PIAS1	MTMR2	OR51B3P
SH3GL1	TGM7		GZMA		WFDC8			IPO4	cat	PIGL	MTPAP	OR51B4
SH3RF2	TKT		GZMK		WIPF2			IQCB1	cat	PKD1	MTRNR2L13	OR51B6
SHOC2	TLR9		HARBI1		WIPF3			ISG15	cat	PKN2	MTX2	OR52J2P
SIK2	TMEM110		HAUS2		WWC1			ITGA2B	cat	PLCL1	MX2	OR52J3
SIPA1L1	TMEM115		HBP1		XCR1			ITGA9	cat	PLEK	MYOM2	OR52M1
SKP2	TMEM17		HEG1		XPC			ITGBL1	cat	POLH	NAALADL2	OR52M2P
SLC12A5	TMEM222		HEMK1		ZFAT			ITPR3	cat	POLR2J	NACAD	OR5H14
SLC16A1	TMEM38A		HERC5		ZFYVE19			JAM3	horse	PON3	NBN	OR5H15
SLC1A1	TMEM62		HHIP		ZMYND10			JMJD1C	cat	POT1	NEB	OR5H5P
SLC2A5	TMEM87A		HIVEP2		ZNF436			JPH3	horse	PPP1R35	NECAB2	OR7E14P
SLC30A2	TNNC1		HMGB3P1		ZNF555			JRKL	dog	PPP3CA	NETO2	PACS1
SLC35B1	TNRC6B		HMGN2		ZNF622			KCNK10	horse	PRDM5	NFE2L3	PARN
SLC4A10	TP53BP1		HNRNPF		ZNF780B			KDM3A	dog	PRKAR2A	NLRP11	PARP14
SLC4A4	TPRKB		HRASLS2		ZZEF1			KDR	cattle	PSKH1	NLRP4	PARVG
SLIT2	TRANK1		HS6ST3					KIAA0226	cat	PSMB10	NLRP7	PAWR
SLITRK1	TRIM37		HSD3B7					KIAA0556	horse	PSMD5	NMD3P1	PC

AMH POOL				DOMESTICATES POOL						GREAT APE POOL (Cagan et al. 2016)		
Peyrégne et al. 2016 (SF1, SF2, Table 2 and Table S7) [375]	Racimo 2016 (Table 3) [326]	Prüfer et al. 2014 (Table S19b.1) [108]	TOTAL AMH (no duplicates) [742]	DOG [229]	CAT [291]	CATTLE [78]	HORSE [109]	TOTAL DOMESTICATES (no duplicates) [691]		Chimp (P.t. troglodytes) [415]	Orango (P. abelii) [500]	(Gorilla) G.g. gorilla [426]
SMAD9	TRIM43		HSDL2					KIAA1549	horse	PTCHD3P1	NMNAT1P5	PCDH15
SNHG4	TRIM69		HSF5					KIF1C	cat	PTENP1	NMUR2	PCDH20
SNRPD1	TRNP1		HSPD1					KIF22	cat	PTPRQ	NNT	PDZD9
SORCS1	TTBK2		HSPE1					KIF27	cat	PYHIN1	NONOP2	PELI3
SORCS2	TUBGCP4		HTR1E					KIF6-like	cattle	R3HCC1L	NOP58	PGAP1
SORCS3	TUSC2		HYAL1					KIRREL2	cat	RAB42	NOS1	PHGDH
SPIDR	TWF2		HYAL2					KIT	cattle	RAB43	NQO2	PKD1
SPOP	TYW5		HYAL3					KLHDC4	horse	RABGAP1L	NR2F1	PLA2G5
SRSF2	UBR1		IFRD2					KRIT1	cat	RABGEF1	NRG3	PLCB1
ST7	UGGT2		IGF1					KYNU	cat	RAD54L2	NRXN1	PLCB3
STAC	UGP2		IGFL2					LAMC2	cat	RANBP10	NRXN3	PLEKHM1
STAG1	UNC50		IGFL3					LAMC3	cattle	RANP1	NTF3	POC1A
STMN2	USP34		IGFL4					LAP3	cat	RASA3	NUP153	POLQ
STX1A	VPS39		IL31RA					LATS2	cat	RASA4B	OBP2B	POLR2J
STX1B	VPS54		IL6ST					LCAT	cat	RBFOX1	OBSCN	PPM1L
SYNPO2	WASF2		IL7					LCLAT1	horse	RBM15B	OR10H1	PPP1R14B
SYT1	WDPCP		INA					LEPREL1	horse	RELA	OR10H5	PRKAG1
SYT6	WDR76		INPP4A					LHFPL3	dog	RHCE	OR10V1	PRKG1
SYVN1	WDR82		INPP5F					LIAS	cat	RHD	OR11G2	PTCD1
TAC4	WDTC1		IQCF1					LILRA6	cattle	RHOA	OR11H5P	PTPRO
TAS2R16	XPO1		IQCF2					LIMD1	cat	RNGTT	OR11H6	PWP1
TBC1D23	ZDHHC18		IQCF3					LIN28B	cattle	RPL23AP1	OR11H7	RAB1B
TBX1	ZEB2		IQCF5					LINC01927	dog	RPL32P1	OR1B1	RAB27A
TDRD3	ZMYND10		IQCF6					LINGO2	dog	RPL3P2	OR1H1P	RASA4B
TDRD7	ZNF106		ITFG1					LMF1	horse	RPL5P18	OR1Q1	RBM14
TFAP2D	ZNF2		ITGA9					LRIG3	dog	RPL7AP7	OR2G2	RBM4
TGM4	ZNF248		ITIH1					LRP1B	cattle	RPLP1P11	OR4K1	RBM4B
THSD7B	ZNF25		ITIH3					LRRC32	cat	RPS4XP6	OR4K15	RERGL
THTPA	ZNF33A		ITIH4					LRRC36	cat	SCARNA20	OR4K16P	RHCE
TLE3	ZNF33B		JMJD6					LRRN3	dog	SCEL	OR4K5	RHD
TLR9	ZNF37A		KAT7					LSM3	cat	SCRN1	OR5AK3P	RHEBL1
TM7SF2	ZNF514		KATNA1					LTF	cattle	SCYL1	OR5AN2P	RIPK2
TMEM123	ZSCAN29		KCNA4					LYST	dog	SDHAP3	OR6Q1	ROR2
TMEM235			KCND2					MAFK	cattle	SDHCP1	OR7E116P	RPL13
TMEM262			KCNH7					MAGEA13P-like	cattle	SETP22	OR7E31P	RPL32P1
TMEM42			KCNIP3					MAOA	dog	SGCB	OR8G1	RPL37P10
TMOD1			KCNJ3					MAOB	dog	SGMS2	OR8G2P	RPL3P2
TNFRSF21			KIAA0825					MAP3K1	dog	SIPA1	OR8G7P	RPRD2
TNRC6B			KIAA1143					MAP3K4	horse	SLC12A4	OR9G2P	RPUSD1
TNS1			KIAA1841					MAP7D2	cat	SLC1A3	OR9G3P	RSL1D1
TP53INP2			KIAA1958					MAP7D3	dog	SLC22A3	OR9G4	RUNDC3B
TPD52			KIF15					MAPK10	horse	SLC4A7	OR9Q1	SCN11A
TRIM71			KIF18A					MARCH10	horse	SLC5A10	OVOL3	SCN9A
TRMT2A			KIFAP3					MARCH7	dog	SLC7A14	PAH	SEC11C
TTC6			KLF2					MARK2	dog	SLC9A9	PAK7	SEC23A
U6			KLHL18					MARK3	dog	SLCO4C1	PAM	SEC24D
U7			KMT2C					MARVELD3	cat	SLIT1	PAQR9	SF3B2
UBE2V2			LARGE1					MATN2	horse	SLIT3	PBRM1	SGCB
UGT8			LCMT2					MBD2	dog	SMIM14	PCDH15	SGCD
USP33			LDHD					MBP	dog	SNX29	PCYOX1	SLC25A13
USP54			LEMD3					MC1R	cattle	SOX5	PEAK1	SLC2A13P1
UTP11			LGALSL					MCF2	dog	SP7	PEBP1P3	SLC4A4
VAPA			LIMK1					MCHR2	dog	SPEF2	PEX26	SLC7A2
VMAC			LIN28A					MED23	dog	SPOCK3	PHKB	SLC8A1
VOPP1			LOH11CR1I					MERTK	cat	SPRR2B	PITRM1	SLCO1B3
VPS51			LPHN3					METAP2	dog	SPRR2G	PLA2G5	SLCO1B7
WBSCR22			LRFN4					METTL22	dog	SSSCA1	PLCE1	SLFN13
WDPCP			LRIG2					METTL8	cat	STAG1	PLEKHA3	SMARCC1
WDR59			LRP1B					MFAP3	cat	STK33	PLEKHN1	SMARCD1
WDR82			LRRC41					MGAM	dog	STXBP6	PLXNB2	SNTG1
WIZ			LRRC57					MGC12345	cattle	SUCLA2P1	PMS1	SNX29
ZBBX			LRRFIP2					MIER3	dog	SUMO1	PNKD	SOD1P3
ZBTB20			LSM1					MIF4GD	dog	SUSD2	PNLDC1	SOGA1
ZBTB34			LSMEM2					MIIP	cat	SVIL	PNPLA5	SP140
ZDHHC8			LSR7					MINOS1	dog	SYNDIG1	POLI	SPAM1
ZFHX4			LYST					MITF	cattle	TACC1P1	POT1	SPATS2L
ZFPL1			MAGI2					MKKS	dog	TAF12	PPARD	SPDL1
ZIC4			MAL					MMP16	dog	TARSL2	PPP2R2B	SPIRE1
ZNF197			MANF					MOB4	dog	TAS1R3	PRICKLE2	SPTBN2
ZNF2			MAP1A					MORC1	cat	TBC1D5	PRKDC	SRIP1
ZNF205			MAP2					MPV17L	dog	TCP10	PRKRA	SRP68
ZNF213			MAP3K6					MRPL11	cat	TCP10L	PRKRIRP6	SSPO
ZNF35			MAP7					MRPL52	cat	TCTA	PRMT8	ST8SIA6
ZNF407			MAPKAPK3					MSI2	horse	TDRD1	PROSER1	STK32B
ZNF501			MATR3					MT1F	dog	TEKT5	PRR4	SYCP2L
ZNF502			MCAM					MT1L	dog	TFAMP2	PRUNE	SYNE1

AMH POOL				DOMESTICATES POOL						GREAT APE POOL (Cagan et al. 2016)		
Peyrégne et al. 2016 (SF1, SF2, Table 2 and Table S7) [375]	***Racimo 2016 (Table 3) [326]***	***Prüfer et al. 2014 (Table S19b.1) [108]***	***TOTAL AMH (no duplicates) [742]***	***DOG [229]***	***CAT [291]***	***CATTLE [78]***	***HORSE [109]***	***TOTAL DOMESTICATES (no duplicates) [691]***		***Chimp (P.t. troglodytes) [415]***	***Orango (P. abelii) [500]***	***(Gorilla) G.g. gorilla [426]***
ZNF514			MCHR1					MT2A	dog	THSD7A	PRUNEP1	SYNPR
ZNF521			MCIDAS					MTIF2	cat	TMC1	PSD3	SYT12
ZNF574			MCM4					MTRF1	cat	TMEM245	PSMC5	TBC1D12
ZNF638			MCMBP					MURC	cat	TMEM50A	PTCD2	TBC1D24
ZNHIT2			MCTP1					MVK	cat	TMPOP1	PTF1A	TBX20
ZNRF1			MDH1					MYBPC1	horse	TNIK	PTPRC	TEC
			MDK					MYLK3	cat	TNR	PWRN4	TERF2IP
			MDM1					MYO15A	cat	TNXB	PYHIN1	THADA
			MED26					MYO9A	cat	TOMM7	PZP	THAP11
			MEGF10					MYOF	dog	TRAF5	RABGAP1L	THSD4
			METTL15					NAPRT1	cat	TRBV25OR9-2	RABGEF1	TMC7
			METTL23					NCAPD3	horse	TRBV26OR9-2	RAD23B	TMEM115
			MFAP1					NCAPG	cattle	TRIM58	RAET1K	TMEM14B
			MFRP					NCOA6	dog	TRPC7	RAET1L	TMEM151A
			MFSD11					NDUFB1	cattle	TSC22D1	RAF1	TMEM50A
			MGAT4A					NEK1	cat	TSC22D4	RASSF6	TMEM87A
			MIB1					NEK4	cat	TSGA10	RBMS3	TMPRSS11A
			MIGA1					NFAM1	cat	TTC19	RCBTB2P1	TRAIP
			MKL1					NFKBIZ	cat	UBE2D2	REL	TRIM42
			MKLN1					NID2	horse	UGDH	RGS3	TSC2
			MLH1					NINJ1	horse	UQCRHP4	RHBDD3	TSNAXIP1
			MMP9					NIPA2	dog	USP32	RHOA	TTC39CP1
			MOB4					NIPBL	horse	USP4	RIMS2	TTC8
			MPND					NKAIN2	dog	USP8P1	RNF213	TTI2
			MRPL49					NOCT	dog	VAV3	RNF6	TUSC2
			MRPS5					NOL4	dog	VN1R66P	RPL12P41	U3
			MTMR4					NOLC1	cat	VN1R67P	RPL27AP	UBE2D3P3
			MTRNR2L7					NOSTRIN	cat	VPRBP	RPL32	UBL5P4
			MUSTN1					NOTCH2	cat	VPS41	RPL32P1	USP24P1
			MYH3					NPAS3	cattle	WASF5P	RPL35P6	USP43
			MYHAS					NPFFR2	cat	XKR3	RPL36AP40	USP46
			MYL4					NPTX1	cattle	XPNPEP3	RPS6P7	USP8P1
			MYLK3					NR2F2	cattle	XPO5	RSPO2	VCAN
			NADK2					NR3C2	horse	YWHAZP4	RTTN	VDAC1P5
			NAT6					NRF1	dog	ZCWPW1	RWDD4P2	VPS13B
			NAT8					NRG2	dog, cat, cattle	ZNF514	SBF2	VPS26BP1
			NAV3					NRG4	cattle	ZNF568	SCARA3	VPS39
			NCOA5					NRXN1	cattle	ZNF670	SCN10A	VPS8
			NCOA6					NT5DC2	horse	ZNF675	SCUBE2	VWF
			NDUFA11					NTAN1	dog	ZNF695	SDHAP3	WASF5P
			NEAT1					NTM	horse	ZNF720	SDK1	WDPCP
			NEB					NUDT15	cat	ZNF725P	SEC11A	WDR95P
			NEDD1					NUMB	horse	ZNF815P	SEC63	WWP2
			NEK4					NUP133	horse	ZNF92	SEMA5B	YIF1A
			NETO2					NUP54	dog	ZPBP	SENP7	ZDHHC20P2
			NEXN					NXPE3	dog	ZSWIM7	SERPINA3	ZDHHC24
			NRG3					OLIG1	cattle		SH2D4A	ZMYM1
			NISCH					OMA1	dog		SLC17A6	ZMYND10
			NLK					OPCML	horse		SLC25A1P2	ZNF394
			NLRX1					OPTC	cat		SLC25A26	ZNF48
			NMUR2					OR10K1	cat		SLC38A8	ZNF493
			NOTO					OR13C8	cattle		SLC6A16	ZNF675
			NPRL2					OR2B11	cat		SLC9C2	ZNF682
			NR0B2					OR4D6	cat		SLFN12	ZNF747
			NR2F1					OR51A7	cattle		SMARCD2	ZNF768
			NT5DC2					OR9A4	dog		SMC3	ZNF771
			NTM					ORF olfactory receptor family cluster (Chromo	cattle		SNRPG	ZNF789
			NTRK2					ORF olfactory receptor family cluster (Chromo	cattle		SOCS2P2	
			NUDC					ORF olfactory receptor family cluster (Chromo	cattle		SOD2	
			NUFIP1					ORF olfactory receptor family cluster (Chromo	cattle		SORBS1	
			NUP37					OTOF	cat		SORBS2	
			NWD1					PAFAH2	cat		SP140	
			NWD2					PARP12	dog		SPATS2L	
			NXPH1					PARVG	cat		SPDL1	
			NYAP2					PCDH18	cattle		SPECC1L	
			ORAI3					PCDHA1	cat		SPINT3	
			OTUD4					PCDHB4	cat		SPOCK3	
			OTX1					PCSK5	horse		SPTLC3	
			PACSIN1					PDE4D	dog		ST6GAL2	
			PAIP2					PDE4DIP	horse		ST6GALNAC3	
			PARP3					PDE5A	horse		STAB2	
			PARPBP					PDE7B	dog		STAT4	
			PATL2					PDILT	dog		STK31	
			PBRM1					PDRG1	horse		STXBP5L	
			PC					PDXDC1	dog		TBC1D22A	
			PCBP4					PEX7	cattle		TBC1D27	

AMH POOL				DOMESTICATES POOL						GREAT APE POOL (Cagan et al. 2016)		
Peyrégne et al. 2016 (SF1, SF2, Table 2 and Table S7) [375]	*Racimo 2016 (Table 3) [326]*	*Prüfer et al. 2014 (Table S19b.1) [108]*	*TOTAL AMH (no duplicates) [742]*	*DOG [229]*	*CAT [291]*	*CATTLE [78]*	*HORSE [109]*	*TOTAL DOMESTICATES (no duplicates) [691]*		Chimp (*P.t. troglodytes*) [415]	Orango (*P. abelii*) [500]	(Gorilla) *G.g. gorilla* [426]
			PCCB					PHF2	horse		TBRG4	
			PCDH17					PHF20	horse		TDP1	
			PCDH9					PHLDB3	cat		TDRKH	
			PCGF6					PIK3C3	horse		TF	
			PCNX					PITRM1	cat		TFG	
			PDCD4					PJA2	cat		TMEM117	
			PDE4B					PKD1L1	dog		TMEM200B	
			PDIA3					PLA2G2E	cat		TMEM40	
			PDZD2					PLA2G3	cat		TMEM5	
			PDZD3					PLAC1	cat		TMPRSS2	
			PELI1					PLAC8L1	cat, cattle		TMPRSS3	
			PEX13					PLAG1	cattle		TNFRSF13B	
			PHACTR1					PLCE1	dog		TNFRSF1B	
			PHF7					PLEKHH1	cat		TNKS1BP1	
			PHKB					PLEKHM3	dog		TNPO1	
			PIGV					PLIN3	cat		TP53BP1	
			PIK3CG					PLXNA4	horse		TPRXL	
			PLA2G16					PML	cat		TPX2	
			PLA2G4D					Pol	cattle		TRAT1	
			PLA2G4E					POLI	dog		TRIM25	
			PLA2GDF					POLR1E	dog		TRIM34	
			PLAC8L1					POP1	horse		TRIM6	
			PLXDC2					PPAP2A	cat		TSG101	
			PMCH					PPAPDC1B	cat		TTC23L	
			POC1A					PPFIBP1	cat		TTN	
			PODXL					PPM1D	horse		TUBA8	
			POMGNT1					PPP1R13B	cat		TUBB4BP5	
			POTEC					PPP2CA	horse		U2SURP	
			POU2F2					PRICKLE4	cat		U3	
			POU3F1					PRKAG1	cat		UBASH3A	
			POU5F2					PRKCZ	horse		UTP20	
			PPAP2A					PRKG2	cat		VN1R20P	
			PPAPDC1A					PRMT3	horse		VN1R31P	
			PPAPDC1B					PRR11	cat		VN1R32P	
			PPIL4					PRX	cat		VN1R33P	
			PPIP5K1					PSMB7	horse		VN2R9P	
			PPM1E					PSPH	cat		VWA5A	
			PPM1M					PSTK	cat		WDFY4	
			PPP2R1B					PTPN4	horse		WDR47	
			PRADC1					PTPRR	cat		WDR62	
			PRDM10					PTPRS	cat		WDR87	
			PRDM2					PUSL1	cat		XKR3	
			PRKAR2B					PVRL3	cattle		YTHDF2	
			PRKCD					Q2ABD2	dog		ZDHHC13	
			PRKDC					RAB3GAP1	dog		ZDHHC20P2	
			PROM2					RABGAP1L	dog		ZNF280D	
			PRR11					RABL3	cat		ZNF33BP1	
			PSTPIP2					RALY	dog		ZNF592	
			PTPN23					RANBP17	dog		ZNF605	
			PTPRD					RAPH1	dog		ZNF7	
			PUS10					RBM11	cat		ZNF718	
			PVRL3					RBP5	cat		ZNF804B	
			QSER1					RCSD1	cat		ZNF850	
			RAB11FIP5					REEP1	dog		ZNF861P	
			RAB28					RELL1	cat		ZSWIM5	
			RABAC1					RELT	dog			
			RAD51C					RFTN2	dog			
			RAD54L					RG9MTD3	dog			
			RAD54L2					RHBDD1	cat			
			RANBP1					RHPN1	horse			
			RANBP3					RIMKLA	cat			
			RARRES3					RNASE6	cat			
			RASA1					RNF103	dog			
			RASGEF1A					RNF144B	cattle			
			RASSF1					RNPC3	dog, cat			
			RASSF3					ROBO1	cattle			
			RB1CC1					RPL3	dog			
			RBFOX2					RPL31	dog			
			RBL1					RRN3	dog			
			RBM14					RRN3P1	dog			
			RBM15B					RRNRP2	dog			
			RBM4					RSL1D1	cat			
			RBM4B					RTP3	cat			
			RBSG3					S100A12	cat			
			RCE1					SAE1	dog			
			REL					SCARB2	dog			

AMH POOL				DOMESTICATES POOL						GREAT APE POOL (Cagan et al. 2016)		
Peyrégne et al. 2016 (SF1, SF2, Table 2 and Table S7) [375]	Racimo 2016 (Table 3) [326]	Prüfer et al. 2014 (Table S19b.1) [108]	TOTAL AMH (no duplicates) [742]	DOG [229]	CAT [291]	CATTLE [78]	HORSE [109]	TOTAL DOMESTICATES (no duplicates) [691]		Chimp (P.t. troglodytes) [415]	Orango (P. abelii) [500]	(Gorilla) G.g. gorilla [426]
			RET					SCN9A	cat			
			RFT1					SCP2D1	dog			
			RFTN2					SCPEP1	horse			
			RGS6					SCRIB	cat			
			RIF1					SDAD1	dog			
			RNF133					SDK2	cat			
			RNF148					SEC24A	cat, horse			
			RNF220					SEC63	horse			
			RNF26					SEMA3D	dog			
			RNF43					SEMA6A	cattle			
			RNF44					SENP5	cat			
			RNPC3					SENP7	cat			
			ROBO2					SEPT10	cat			
			ROCK1					SERINC3	cat			
			RPL13AP6					SETBP1	dog			
			RPL29					SETD9	dog			
			RPS18P9					SETMAR	cattle			
			RPS6KA1					SF3B1	dog			
			RRP9					SGCD	horse			
			RSPO3					SH2D5	cat			
			SAMHD1					SH3GL2	dog			
			SCAP					SHC4	cat			
			SCMH1					SIAE	cat			
			SCYL3					SKA2	dog			
			SEC23IP					SKP1	horse			
			SEC24D					SLC22A13	cat			
			SEMA3F					SLC22A15	horse			
			SEMA3G					SLC22A18	cat			
			SEMA6D					SLC25A38	cat			
			SEPT4					SLC35D1	cattle			
			SERF2					SLC35F5	cat			
			SERINC4HYPK					SLC39A7	cat			
			SESN1					SLC39A8	cat			
			SETD1A					SLC41A2	cattle			
			SF3A3					SLC43A1	horse			
			SF3B1					SLC46A1	cat			
			SFMBT1					SLC5A1	dog			
			SFXN5					SLC5A4	dog			
			SGMS2					SLC6A1	cattle			
			SGSM3					SLC6A17	dog			
			SH3GL1					SLC9A6	dog			
			SH3RF2					SLCO1A2	cat			
			SHOC2					SMC4	dog			
			SIK2					SMG1	cat			
			SIN3B					SMG6	cat, horse			
			SIPA1L1					SMIM23	dog			
			SKA2					SMO	dog			
			SKIV2L2					SMYD2	dog			
			SKP2					SNAP29	dog			
			SLC12A5					SNRPD1	cattle			
			SLC16A1					SPATA19	horse			
			SLC1A1					SPATA21	cat			
			SLC25A17					SPATA7	cat			
			SLC26A3					SPERT	cat			
			SLC26A4					SPHKAP	cat			
			SLC2A5					SPINT1	cat			
			SLC30A2					SPTAN1	cattle			
			SLC35B1					SPTBN5	cat			
			SLC35E1					SREBF1	cat			
			SLC38A9					SRP72	dog			
			SLC4A10					SRRM2	cat			
			SLC4A4					STAB1	horse			
			SLC9A1					STARD5	cat			
			SLFNL1					STARD6	dog			
			SLIT2					STK10	dog, cattle			
			SLITRK1					STK11IP	cat			
			SLITRK3					STS	cat			
			SMAD1					STX7	dog			
			SMAD9					STXBP6	horse			
			SMG8					SUN3	cat			
			SMIM4					SURF2	cat			
			SMIM7					SUSD3	horse			
			SMYD5					SYNM	cat			
			SNAI2					SYTL1	cat			
			SNAP23					TAOK1	cattle			
			SNF					TAS2R1	cat			

AMH POOL				DOMESTICATES POOL						GREAT APE POOL (Cagan et al. 2016)		
Peyrégne et al. 2016 (SF1, SF2, Table 2 and Table S7) [375]	*Racimo 2016 (Table 3) [326]*	*Prüfer et al. 2014 (Table S19b.1) [108]*	*TOTAL AMH (no duplicates) [742]*	*DOG [229]*	*CAT [291]*	*CATTLE [78]*	*HORSE [109]*	*TOTAL DOMESTICATES (no duplicates) [691]*		Chimp (*P.t. troglodytes*) [415]	Orango (*P. abelii*) [500]	(Gorilla) *G.g. gorilla* [426]
			SNHG4					TAS2R16	cattle			
			SNRPD1					TAS2R3	cat			
			SORCS1					TAS2R38	dog			
			SORCS2					TBC1D9	dog			
			SORCS3					TBXAS1	dog			
			SPATS2L					TCTN1	horse			
			SPCS1					TCTN3	dog			
			SPG11					TEKT3	dog			
			SPIDR					TEX14	cat			
			SPOP					TF	cat			
			SPTBN2					TFCP2L1	cattle			
			SRSF2					TH	dog			
			ST7					THBS2	cat			
			STAB1					THEGL	dog			
			STAC					THUMPD1	cat			
			STAG1					THYN1	horse			
			STAMBP					TLX3	dog			
			STARD9					TMEM114	dog			
			STK3					TMEM132D	dog, cattle			
			STMN2					TMEM159	dog			
			STRC					TMEM242	dog			
			STX1A					TMEM59L	cat			
			STX1B					TMEM71	cat			
			SUPT4H1					TNFRSF9	cattle			
			SYNPO2					TNKS2	dog			
			SYT1					TOE1	cat			
			SYT6					TP53BP1	cat			
			SYTL1					TRAPPC8	horse			
			SYVN1					TRBV25OR9-2	dog			
			TAC4					TRIM16	dog			
			TAF5					TRIM59	dog			
			TANC2					TRIO	horse			
			TAS2R16					TRMT61A	dog			
			TBC1D23					TRPV6	cat			
			TBX1					TRY1	dog			
			TDRD3					TRY2	dog			
			TDRD7					TRY3	dog			
			TEX14					TSTD2	cat			
			TEX264					TTC39A	dog			
			TFAP2D					TUBGCP5	dog			
			TGM4					TVP23B	dog			
			TGM5					TVP23C	dog			
			TGM7					TXN2	cat			
			THSD7B					TXNRD2	cat			
			THTPA					TYK2	cat			
			TK2					U2	dog			
			TKT					UBE2B	horse			
			TLE3					UBXN10	cat			
			TLR9					ULBP3	cattle			
			TM7SF2					UMOD	dog			
			TMEM110					UNC93A	cattle			
			TMEM115					URB2	horse			
			TMEM123					USP45	cat			
			TMEM17					UVRAG	cat			
			TMEM222					V1R	dog			
			TMEM235					VDAC1	horse			
			TMEM262					VEZT	dog, cat			
			TMEM38A					VPS26B	horse			
			TMEM42					VRK1	horse			
			TMEM62					VWC2	dog			
			TMEM87A					VWDE	cattle			
			TMOD1					WASF3	horse			
			TNFRSF21					WDR17	cat			
			TNNC1					WDR62	cat			
			TNRC6B					WDR90	cat			
			TNS1					WFDC8	cat			
			TP53BP1					WIPF2	cat			
			TP53INP2					WIPF3	cat			
			TPD52					WNK2	horse			
			TPRKB					WWC1	cat			
			TRANK1					XCR1	cat			
			TRIM37					XPBP	dog			
			TRIM43					XPC	cat			
			TRIM69					XPO6	cattle			
			TRIM71					YSK4	dog			
			TRMT2A					YWHAH	dog			

AMH POOL				DOMESTICATES POOL						GREAT APE POOL (Cagan et al. 2016)		
Peyrégne et al. 2016 (SF1, SF2, Table 2 and Table S7) [375]	***Racimo 2016 (Table 3) [326]***	***Prüfer et al. 2014 (Table S19b.1) [108]***	***TOTAL AMH (no duplicates) [742]***	***DOG [229]***	***CAT [291]***	***CATTLE [78]***	***HORSE [109]***	***TOTAL DOMESTICATES (no duplicates) [691]***		**Chimp (*P.t. troglodytes*) [415]**	**Orango (*P. abelii*) [500]**	**(Gorilla) *G.g. gorilla* [426]**
			TRNP1					ZC3H3	horse			
			TTBK2					ZFAT	cat			
			TTC6					ZFYVE19	cat			
			TUBGCP4					ZMYND10	cat			
			TUSC2					ZNF236	dog			
			TWF2					ZNF286A	dog			
			TYW5					ZNF286B	dog			
			U6					ZNF436	cat			
			U7					ZNF492	dog			
			UBE2V2					ZNF516	dog			
			UBR1					ZNF521	cattle			
			UGGT2					ZNF555	cat			
			UGP2					ZNF622	cat			
			UGT8					ZNF679	dog			
			UNC50					ZNF780B	cat			
			UQCRH					ZP2	dog			
			UQCRHL					ZPBP	dog			
			USP33					ZZEF1	cat			
			USP34									
			USP54									
			UTP11									
			VAPA									
			VMAC									
			VOPP1									
			VPS39									
			VPS51									
			VPS54									
			WASF2									
			WBSCR22									
			WDPCP									
			WDR59									
			WDR76									
			WDR82									
			WDTC1									
			WHSC1L1									
			WIZ									
			XPO1									
			ZBBX									
			ZBTB20									
			ZBTB34									
			ZDHHC18									
			ZDHHC8									
			ZEB2									
			ZFHX4									
			ZFPL1									
			ZIC4									
			ZMYND10									
			ZNF106									
			ZNF197									
			ZNF2									
			ZNF205									
			ZNF213									
			ZNF248									
			ZNF25									
			ZNF33A									
			ZNF33B									
			ZNF35									
			ZNF37A									
			ZNF407									
			ZNF501									
			ZNF502									
			ZNF514									
			ZNF521									
			ZNF574									
			ZNF638									
			ZNF852									
			ZNHIT2									
			ZNRF1									
			ZSCAN29									

(B) Contrasts between domesticates, between AMH and each domesticate, and between great apes and domesticates

	dog = **229**	cat = **291**	cattle = **78**	horse = **109**	AMH = **742**
dog = **229**		v = 5; p = 0.2575646	**v = 5; p = 0.002250835**	v = 0	**v = 15; p = 0.02934342**
cat = **291**	v = 5; p = 0.2575646		v = 3; p = 0.1111013	v = 4; p = 0.0805177	v = 15; p = 0.1454225
cattle = **78**	**v = 5; p = 0.002250835**	v = 3; p = 0.1111013		v = 0	**v = 9; p = 0.002784603**
horse = **109**	v = 0	v = 4; p = 0.0805177	v = 0		v = 7; p = 0.1219762

p < 0.05 (orange)
p < 0.01 (red)

Intersections between domesticates				Intersections between total AMH and each domesticate			
**DOG-CAT [5]**	**DOG-CATTLE [5] ****	**CAT-CATTLE [3]**	**CAT-HORSE [4]**	**AMH-DOG [15] ***	**AMH-CAT [15]**	**AMH-CATTLE [9] ****	**AMH-HORSE [7]**
ATXN7L1	FAM172A	ADAMTS13	BRAF	COA5	BRAF	ERBB4	AMBRA1
CLEC5A	GRIK3	NRG2	DCC	COL11A1	GRIA1	FAM172A	BRAF
NRG2	NRG2	PLAC8L1	SEC24A	COQ10B	HSD3B7	GRIK3	CACNA1D
RNPC3	STK10		SMG6	FAM172A	ITGA9	LRP1B	DLGAP1
VEZT	TMEM132D			GGT7	MYLK3	PLAC8L1	NT5DC2
				GRIK3	NEK4	PVRL3	NTM
				HSPD1	PLAC8L1	SNRPD1	STAB1
				HSPE1	PPAP2A	TAS2R16	
				LYST	PPAPDC1B	ZNF521	
				MOB4	PRR11		
				NCOA6	RNPC3		
				RFTN2	SYTL1		
				RNPC3	TEX14		
				SF3B1	TP53BP1		
				SKA2	ZMYND10		

* p < 0.05
** p < 0.01

Intersections between great apes and domesticates					
**DOM-CHIMP [16]**		**DOM-ORANGUTAN [20]**		**DOM-GORILLA [12]**	
ALK	horse	ACOX3	cat	CUX2	dog
AMBRA1	horse	ADAMDEC1	cat	DNAH9	horse
C3orf62	cat	ADAMTSL3	cat	FER	cat
EHBP1L1	cat	ALK	horse	IMMP2L	dog
FAF1	horse	BARD1	cat	MRPL11	cat
HADH	cat	DNAJB9	cat	OPCML	horse
INPP4B	cat	FAM172A	cattle, dog	PARVG	cat
LCAT	cat	ISG15	cat	PRKAG1	cat
LHFPL3	dog	KDM3A	dog	RSL1D1	cat
MGAM	dog	KYNU	cat	SCN9A	cat
NIPBL	horse	NRXN1	cattle	SGCD	horse
NXPE3	dog	PITRM1	cat	ZMYND10	cat
RABGAP1L	dog	PLCE1	dog		
STXBP6	horse	POLI	dog		
TRBV25OR9-2	dog	RABGAP1L	dog		
ZPBP	dog	SEC63	horse		
		SENP7	cat		
		TF	cat		
		TP53BP1	cat		
		WDR62	cat		

(C) Results of hypergeometric intersection tests on data from S4 Table (B)

		Domesticates				
		Dog = **229**	Cat = **291**	Cattle = **78**	Horse = **109**	Total domesticates = **691**
AMH	Peyrégne et al. 2016 = **375**	-	-	-	-	*v* = 18; *p* = 0.1199135
	Racimo 2016 = **326**	-	-	-	-	*v* = 16; *p* = 0.1191519
	Prüfer et al. 2014 = **108**	-	-	-	-	*v* = 9; *p* = 0.01456058 *
	Prüfer et al. 2014 + Racimo 2016 = **419**	-	-	-	-	*v* = 24; *p* = 0.01478488 *
	Total AMH (Peyrégne et al. 2016, Racimo 2016, Prüfer et al. 2014) = **742**	*v* = 15; *p* = 0.02934342 *	*v* = 15; *p* = 0.1454225	*v* = 9; *p* = 0.002784603 **	*v* = 7; *p* = 0.1219762	*v* = 41; *p* = 0.003407041 **
Great apes (Cagan et al. 2016)	Chimpanzee (*Pan t. troglodytes*) = **415**	*v* = 6; *p* = 0.3614671	*v* = 5; *p* = 0.7449666	*v* = 0	*v* = 5; *p* = 0.08336393	*v* = 16; *p* = 0.4008807
	Orango (*Pongo abelii*) = **500**	*v* = 5; *p* = 0.7023469	*v* = 12; *p* = 0.07285972	*v* = 2; *p* = 0.598045	*v* = 2; *p* = 0.7728682	*v* = 20; *p* = 0.3207204
	Gorilla (*G. g. gorilla*) = **426**	*v* = 2; *p* = 0.9619034	*v* = 7; *p* = 0.4519085	*v* = 0	*v* = 3; *p* = 0.4267467	*v* = 12; *p* = 0.8291688
