# Supplementary material for: Self-domestication in Homo sapiens: Insights from comparative genomics
Source: PLoS One. 2017 Oct 18;12(10):e0185306. doi: 10.1371/journal.pone.0185306 (PMC5646786; doi:10.1371/journal.pone.0185306)
Supplement: S5 Table — Gene lists and statistical analysis of data from AMH and non-domesticated species. (PDF) [file pone.0185306.s006.pdf]

S5 Table. Genes overlapping between AMH and non-domesticated *Canis* (grey wolf), and non-domesticated bovine (wisent, European bison)

(A) Genes overlapping between AMH and grey wolf. The overlap between each list and AMH is given in square brackets and overlapping genes are highlighted in pink. **Sources:** *Stronen et al. 2015* http://onlinelibrary.wiley.com/doi/10.1002/ece3.1695/full
Pilot et al. 2014 http://www.nature.com/hdy/journal/v112/n4/full/hdy2013122a.html

AMH	***Stronen et al. T2 [3]***	***Stronen et al. TS3 [0]***	***Stronen et al. TS5 [1]***	***Pilot et al. TS4 [1]***
ABCE1	ADAMTS1	ADCY5	AGER	ADAMTS3
ABHD14A	ADORA2A	ADORA2A	ATXN7	ADD2
ABHD14B	AGTR1	AGER	BHLHE41	AMBP
ABHD3	ANO3	AIRE	CLSTN2	ASTN2
ACE	ATP2B1	APP	COL17A1	CACNA2D3
ACTG1P4	BMP7	ATXN7	cOR1P2	CIDEC
ACTG2	COL9A3	BHLHE41	CPT1A	CX984702
ACY1	CP	CAFA-T2R46	CTNNA2	CX987143
ADAL	CPT1A	CAFA-T2R67	DARS	DMGDH
ADRA2A	CRAT	CALB1	DDX39B	DOK5
ADRA2B	DARS	CLDN9	DLA-12	EFCAB11
ADSL	DNM1	CLSTN2	DLA-64	ENO1
AGO1	FGF4	COL17A1	DLA88	FOXP1
AGO3	FNIP2	COMT	DLA-DMB	GCNT2
AHDC1	HPS3	cOR10A3	DLA-DQA1	GLP1R
AHSA2	HPS5	cOR10A9	DLA-DQB1	GRID1
AIDA	IGFI1R	cOR10AB2	DLA-DRA	Hbs11
AIG1	KIT	cOR1P2	FGF4	INTS10
AKAP8	LEPR	cOR5E1P	HLA-DRB1	KANK4
AKAP8L	NAV2	CSDA	HPS5	KCNK5
AKR7A2P1	NPYR1	CTNNA2	LY6G5C	Lactb11
AL122050	PPFIBP2	DDX39B	NEO1	NAALAD2
ALAS1	RPTOR	DLA12	OR01E11	NR4A2
ALG9	RSPO2	DLA64	OR08H10	PDGFA
ALMS1	SCD5	DLA88	OR3A2	PRELID2
AMBRA1	SGIP1	DLA-DMB	PDE6D	SETD4
AMPH	SLC5A1	DLA-DQA1	PSMB8	SH3RF1
AMY1A	SPRCS3	DLA-DQB1	SCD5	THBS1
AMY1B	TK2	DLA-DRA	SGIP1	TXNL1
AMY1C	TRPV1	DLG2	TNF	URI1
AMY2A	TRPV3	DSC1	TPH1	ZCWPW1
AMY2B	ZFR	DSG1	TRPV1	ZMAT4
ANAPC10		DSG3	TRPV3	
ANK2		FGD4		
ANKRD30A		HLA-DRB1		
ANKRD30B		HSF4		
ANKRD32		HTR2A		
ANKRD55		IL10		
ANO10		IL13		
ANO3		IL4		
ANXA2		IL5		
ARHGAP1		ITGB2		
ARHGAP15		LY6G5C		
ARID1A		NEO1		
ARSJ		OLFM2		
ASAP2		OR01E11		
ASIC2		OR08C06		
ASTL		OR08H09		
ATG10		OR08H10		
ATG13		OR10A4		
ATP1A3		OR10B10		
ATXN10		OR10F05		
B2M		OR2D3		
BAG4		OR3A2		
BAI3		OR4B06		
BAP1		OR7G2		
BBIP1		PDE6B		

AMH	*Stronen et al. T2 [3]*	*Stronen et al. TS3 [0]*	*Stronen et al. TS5 [1]*	*Pilot et al. TS4 [1]*
BCAP29		PDE6D		
BCAR3		PEBP1		
BCL2		PSMB8		
BEAN1		SAG		
BIRC2		TAS2R42		
BMS1		TASR10		
BRAF		TASR42		
BRD4		TASR7		
BROX		TNF		
BZRAP1		TPH1		
C11orf1		ULK1		
C11orf80		USH2A		
C16orf87		YWHAH		
C17orf47				
C18orf42				
C19orf44				
C1orf112				
C1orf172				
C1orf190				
C1QTNF5				
C2orf47				
C2orf69				
C2orf78				
C3orf18				
C3orf35				
CACNA1D				
CACNA2D1				
CACNA2D2				
CADPS				
CADPS2				
CALN1				
CALR3				
CAMK1G				
CAPN3				
CAPN5				
CAPS				
CASC4				
CASP16P				
CATSPER2				
CBL				
CBLL1				
CBLN4				
CCDC153				
CCDC188				
CCDC192				
CCDC53				
CCNDBP1				
CCNH				
CCNJL				
CCNO				
CCT7				
CD164L2				
CDAN1				
CDC20B				
CDC27				
CDC42EP3				
CDH10				
CEBPD				
CELF4				
CEP41				
CEP57L1				
CHERP				
CHODL				
CHRM4				

AMH	*Stronen et al. T2 [3]*	*Stronen et al. TS3 [0]*	*Stronen et al. TS5 [1]*	*Pilot et al. TS4 [1]*
CISH				
CKLF				
CKMT1A				
CKMT1B				
CLASP2				
CLDN10				
CLSTN1				
CMTM1				
CNGA3				
CNTNAP4				
COA5				
COG5				
COL11A1				
COQ10B				
CORO2B				
CR2				
CSGALNACT2				
CSMD2				
CSPG5				
CTDSPL2				
CTNNBL1				
CTNND2				
CTPS				
CTXN3				
CXCL13				
CXCL3				
CYB561				
CYB561D2				
DAPP1				
DBIL5P2				
DDHD2				
DDX4				
DGCR8				
DGKZ				
DGUOK				
DHDDS				
DHRS12				
DHX29				
DLGAP1				
DNAH1				
DNAJA2				
DNAJB4				
DNAJC3				
DOCK3				
DPYSL5				
DRAM1				
DTNA				
DUS4L				
DUSP11				
DUSP7				
DYNC1H1				
DYSF				
DZIP1				
E2F6				
EFCC1				
EGR4				
EHBP1				
EIF3J				
ELAVL4				
ELL3				
ELN				
ELP6				
ENTHD1				
EPB42				

AMH	*Stronen et al. T2 [3]*	*Stronen et al. TS3 [0]*	*Stronen et al. TS5 [1]*	*Pilot et al. TS4 [1]*
EPM2AIP1				
EPS15L1				
EPSTI1				
ERBB4				
ESCO1				
ESM1				
EXOC6B				
EXTL1				
FAAH				
FAF2				
FAHD2A				
FAM117A				
FAM150A				
FAM172A				
FAM177B				
FAM19A3				
FAM46B				
FAM49B				
FAM83F				
FAU				
FBXL19				
FBXO41				
FBXW7				
FCN3				
FDXACB1				
FERMT2				
FGF12				
FGF14				
FHL3				
FHOD3				
FIBCD1				
FKSG51				
FLJ35017				
FLJ39294				
FLJ45513				
FNBP1L				
FOXO1				
FRMD5				
FRMD8				
FSTL5				
FUBP1				
FUT5				
FXYD4				
FZD3				
GABRB3				
GALNT10				
GALNT11				
GALNT2				
GALNTL5				
GANC				
GATA6				
GBP2				
GBP4				
GBP5				
GBP7				
GCNT2				
GDAP1				
GDF6				
GDPD1				
GGT7				
GINM1				
GK2				
GLI3				
GLT8D1				

AMH	*Stronen et al. T2 [3]*	*Stronen et al. TS3 [0]*	*Stronen et al. TS5 [1]*	*Pilot et al. TS4 [1]*
GLYCTK				
GNAI2				
GNAT1				
GNL3				
GOLGA4				
GP9				
GPAT2				
GPATCH3				
GPM6A				
GPN2				
GPR22				
GPR3				
GPR39				
GPR62				
GPT2				
GPX8				
GRAP2				
GREB1L				
GRIA1				
GRID2				
GRIK3				
GRIK5				
GRM2				
GRM3				
GTDC1				
GTF3C5				
GYPA				
GYPB				
GZMA				
GZMK				
HARBI1				
HAUS2				
HBP1				
HEG1				
HEMK1				
HERC5				
HHIP				
HIVEP2				
HMGB3P1				
HMGN2				
HNRNPF				
HRASLS2				
HS6ST3				
HSD3B7				
HSDL2				
HSF5				
HSPD1				
HSPE1				
HTR1E				
HYAL1				
HYAL2				
HYAL3				
IFRD2				
IGF1				
IGFL2				
IGFL3				
IGFL4				
IL31RA				
IL6ST				
IL7				
INA				
INPP4A				
INPP5F				
IQCF1				

AMH	*Stronen et al. T2 [3]*	*Stronen et al. TS3 [0]*	*Stronen et al. TS5 [1]*	*Pilot et al. TS4 [1]*
IQCF2				
IQCF3				
IQCF5				
IQCF6				
ITFG1				
ITGA9				
ITIH1				
ITIH3				
ITIH4				
JMJD6				
KAT7				
KATNA1				
KCNA4				
KCND2				
KCNH7				
KCNIP3				
KCNJ3				
KIAA0825				
KIAA1143				
KIAA1841				
KIAA1958				
KIF15				
KIF18A				
KIFAP3				
KLF2				
KLHL18				
KMT2C				
LARGE1				
LCMT2				
LDHD				
LEMD3				
LGALSL				
LIMK1				
LIN28A				
LOH11CR1I				
LPHN3				
LRFN4				
LRIG2				
LRP1B				
LRRC41				
LRRC57				
LRRFIP2				
LSM1				
LSMEM2				
LSR7				
LYST				
MAGI2				
MAL				
MANF				
MAP1A				
MAP2				
MAP3K6				
MAP7				
MAPKAPK3				
MATR3				
MCAM				
MCHR1				
MCIDAS				
MCM4				
MCMBP				
MCTP1				
MDH1				
MDK				
MDM1				

AMH	*Stronen et al. T2 [3]*	*Stronen et al. TS3 [0]*	*Stronen et al. TS5 [1]*	*Pilot et al. TS4 [1]*
MED26				
MEGF10				
METTL15				
METTL23				
MFAP1				
MFRP				
MFSD11				
MGAT4A				
MIB1				
MIGA1				
MKL1				
MKLN1				
MLH1				
MMP9				
MOB4				
MPND				
MRPL49				
MRPS5				
MTMR4				
MTRNR2L7				
MUSTN1				
MYH3				
MYHAS				
MYL4				
MYLK3				
NADK2				
NAT6				
NAT8				
NAV3				
NCOA5				
NCOA6				
NDUFA11				
NEAT1				
NEB				
NEDD1				
NEK4				
NETO2				
NEXN				
NFG3				
NISCH				
NLK				
NLRX1				
NMUR2				
NOTO				
NPRL2				
NR0B2				
NR2F1				
NT5DC2				
NTM				
NTRK2				
NUDC				
NUFIP1				
NUP37				
NWD1				
NWD2				
NXPH1				
NYAP2				
ORAI3				
OTUD4				
OTX1				
PACSIN1				
PAIP2				
PARP3				
PARPBP				

AMH	*Stronen et al. T2 [3]*	*Stronen et al. TS3 [0]*	*Stronen et al. TS5 [1]*	*Pilot et al. TS4 [1]*
PATL2				
PBRM1				
PC				
PCBP4				
PCCB				
PCDH17				
PCDH9				
PCGF6				
PCNX				
PDCD4				
PDE4B				
PDIA3				
PDZD2				
PDZD3				
PELI1				
PEX13				
PHACTR1				
PHF7				
PHKB				
PIGV				
PIK3CG				
PLA2G16				
PLA2G4D				
PLA2G4E				
PLA2GDF				
PLAC8L1				
PLXDC2				
PMCH				
POC1A				
PODXL				
POMGNT1				
POTEC				
POU2F2				
POU3F1				
POU5F2				
PPAP2A				
PPAPDC1A				
PPAPDC1B				
PPIL4				
PPIP5K1				
PPM1E				
PPM1M				
PPP2R1B				
PRADC1				
PRDM10				
PRDM2				
PRKAR2B				
PRKCD				
PRKDC				
PROM2				
PRR11				
PSTPIP2				
PTPN23				
PTPRD				
PUS10				
PVRL3				
QSER1				
RAB11FIP5				
RAB28				
RABAC1				
RAD51C				
RAD54L				
RAD54L2				
RANBP1				

AMH	*Stronen et al. T2 [3]*	*Stronen et al. TS3 [0]*	*Stronen et al. TS5 [1]*	*Pilot et al. TS4 [1]*
RANBP3				
RARRES3				
RASA1				
RASGEF1A				
RASSF1				
RASSF3				
RB1CC1				
RBFOX2				
RBL1				
RBM14				
RBM15B				
RBM4				
RBM4B				
RBSG3				
RCE1				
REL				
RET				
RFT1				
RFTN2				
RGS6				
RIF1				
RNF133				
RNF148				
RNF220				
RNF26				
RNF43				
RNF44				
RNPC3				
ROBO2				
ROCK1				
RPL13AP6				
RPL29				
RPS18P9				
RPS6KA1				
RRP9				
RSPO3				
SAMHD1				
SCAP				
SCMH1				
SCYL3				
SEC23IP				
SEC24D				
SEMA3F				
SEMA3G				
SEMA6D				
SEPT4				
SERF2				
SERINC4HYPK				
SESN1				
SETD1A				
SF3A3				
SF3B1				
SFMBT1				
SFXN5				
SGMS2				
SGSM3				
SH3GL1				
SH3RF2				
SHOC2				
SIK2				
SIN3B				
SIPA1L1				
SKA2				
SKIV2L2				

AMH	*Stronen et al. T2 [3]*	*Stronen et al. TS3 [0]*	*Stronen et al. TS5 [1]*	*Pilot et al. TS4 [1]*
SKP2				
SLC12A5				
SLC16A1				
SLC1A1				
SLC25A17				
SLC26A3				
SLC26A4				
SLC2A5				
SLC30A2				
SLC35B1				
SLC35E1				
SLC38A9				
SLC4A10				
SLC4A4				
SLC9A1				
SLFNL1				
SLIT2				
SLITRK1				
SLITRK3				
SMAD1				
SMAD9				
SMG8				
SMIM4				
SMIM7				
SMYD5				
SNAI2				
SNAP23				
SNF				
SNHG4				
SNRPD1				
SORCS1				
SORCS2				
SORCS3				
SPATS2L				
SPCS1				
SPG11				
SPIDR				
SPOP				
SPTBN2				
SRSF2				
ST7				
STAB1				
STAC				
STAG1				
STAMBP				
STARD9				
STK3				
STMN2				
STRC				
STX1A				
STX1B				
SUPT4H1				
SYNPO2				
SYT1				
SYT6				
SYTL1				
SYVN1				
TAC4				
TAF5				
TANC2				
TAS2R16				
TBC1D23				
TBX1				
TDRD3				

AMH	*Stronen et al. T2 [3]*	*Stronen et al. TS3 [0]*	*Stronen et al. TS5 [1]*	*Pilot et al. TS4 [1]*
TDRD7				
TEX14				
TEX264				
TFAP2D				
TGM4				
TGM5				
TGM7				
THSD7B				
THTPA				
TK2				
TKT				
TLE3				
TLR9				
TM7SF2				
TMEM110				
TMEM115				
TMEM123				
TMEM17				
TMEM222				
TMEM235				
TMEM262				
TMEM38A				
TMEM42				
TMEM62				
TMEM87A				
TMOD1				
TNFRSF21				
TNNC1				
TNRC6B				
TNS1				
TP53BP1				
TP53INP2				
TPD52				
TPRKB				
TRANK1				
TRIM37				
TRIM43				
TRIM69				
TRIM71				
TRMT2A				
TRNP1				
TTBK2				
TTC6				
TUBGCP4				
TUSC2				
TWF2				
TYW5				
U6				
U7				
UBE2V2				
UBR1				
UGGT2				
UGP2				
UGT8				
UNC50				
UQCRH				
UQCRHL				
USP33				
USP34				
USP54				
UTP11				
VAPA				
VMAC				
VOPP1				

AMH	*Stronen et al. T2 [3]*	*Stronen et al. TS3 [0]*	*Stronen et al. TS5 [1]*	*Pilot et al. TS4 [1]*
VPS39				
VPS51				
VPS54				
WASF2				
WBSCR22				
WDPCP				
WDR59				
WDR76				
WDR82				
WDTC1				
WHSC1L1				
WIZ				
XPO1				
ZBBX				
ZBTB20				
ZBTB34				
ZDHHC18				
ZDHHC8				
ZEB2				
ZFHX4				
ZFPL1				
ZIC4				
ZMYND10				
ZNF106				
ZNF197				
ZNF2				
ZNF205				
ZNF213				
ZNF248				
ZNF25				
ZNF33A				
ZNF33B				
ZNF35				
ZNF37A				
ZNF407				
ZNF501				
ZNF502				
ZNF514				
ZNF521				
ZNF574				
ZNF638				
ZNF852				
ZNHIT2				
ZNRF1				
ZSCAN29				

(B) Genes overlapping between AMH and non-domesticated bovine (wisent, European bison)

The overlap between each list and AMH is given in square brackets and overlapping genes are highlighted in pink.

Sources:

Gautier et al. 2016 https://academic.oup.com/mbe/article/33/11/2801/2271657/Deciphering-the-Wisent-Demographic-and-Adaptive

Wang et al. 2017 https://academic.oup.com/gigascience/article/3065124

AMH	***Gautier et al. TS3 [11]***	***Wang et al. 2017 (S14) [3]***
ABCE1	4833423E24Rik	ABCB4
ABHD14A	4930503B20Rik	ABCC4
ABHD14B	ACADL	ASPG
ABHD3	ACBD5	ATP1A3
ACE	Ace3	ATP2B2
ACTG1P4	ACSM1	BRSK2
ACTG2	ADAM32	BRWD1
ACY1	ADAT1	CASP8AP2
ADAL	ADGRE3	CD97
ADRA2A	ADH7	CDC42BPG
ADRA2B	AGER	CELF4
ADSL	AMHR2	CNTNAP1
AGO1	ANGPTL3	COL1A1
AGO3	ANKDD1B	DIAPH3
AHDC1	ANKRD31	EHMT1
AHSA2	ANKZF1	FOXP1
AIDA	APLF	FZD8
AIG1	APOBEC3A	GNAS
AKAP8	APOBEC3B	HCN3
AKAP8L	APOF	HOXA3
AKR7A2P1	AQP8	HSPA4L
AL122050	ARHGAP29	KCNH6
ALAS1	ARL13A	KCNK1
ALG9	ARPC2	KLHL21
ALMS1	AU018091	LILRB3
AMBRA1	BC026585	LMNB2
AMPH	BCKDHB	MAPK3K13
AMY1A	BCO2	MAPK8IP3
AMY1B	BLZF1	MAT2B
AMY1C	BPHL	MUC20
AMY2A	BRIP1	MYO5B
AMY2B	BTBD8	NACC1
ANAPC10	Btnl1	NEFH
ANK2	C12orf71	NOS3
ANKRD30A	C14orf39	OR11G2
ANKRD30B	C14orf79	OR12D2
ANKRD32	C17orf53	PARPBP
ANKRD55	C19orf57	PDE6A
ANO10	C1orf112	PLIN4
ANO3	C1orf158	PTH1R
ANXA2	C1orf87	PTK2B
ARHGAP1	C2orf80	RADIL
ARHGAP15	C4BPA	RANBP17
ARID1A	C6orf141	SEPT8
ARSJ	C7orf50	SLC12A7
ASAP2	C9orf43	SLC24A1
ASIC2	C9orf50	SLC4A8
ASTL	CCDC102B	SMARCA2
ATG10	CCDC154	SYNGAP1
ATG13	CCDC185	TFE3
ATP1A3	CCDC190	TOMM20
ATXN10	CCDC30	TRAF3IP1
B2M	CCDC57	UNC93B1
BAG4	CCDC66	XP_001249346.4
BAI3	Ccdc71	XP_001249583.2
BAP1	CCDC78	XP_001251951.2
BBIP1	CCDC79	XP_001255218.2
BCAP29	CCDC82	XP_001255375.1
BCAR3	CCL16	XP_001255473.3
BCL2	CCL24	XP_003584089.2
BEAN1	CCL5	XP_005193688.1

AMH	*Gautier et al. TS3 [11]*	*Wang et al. 2017 (S14) [3]*
BIRC2	CD180	XP_005195701.1
BMS1	CD1E	XP_005195799.1
BRAF	CD244	XP_005197067.1
BRD4	CD4	XP_005197353.1
BROX	CD44	XP_005198975.1
BZRAP1	CD48	XP_588462.3
C11orf1	CD55	XP_592549.4
C11orf80	CD72	XP_594531.4
C16orf87	CD9	XP_600867.4
C17orf47	CDC25C	XP_869208.6
C18orf42	CDCA4	ZNF526
C19orf44	CDHR4	
C1orf112	CDKN2A	
C1orf172	CEACAM18	
C1orf190	CENPQ	
C1QTNF5	CER1	
C2orf47	CFH	
C2orf69	CIDEC	
C2orf78	CKAP2L	
C3orf18	CLCA4	
C3orf35	CLDN15	
CACNA1D	CLDN25	
CACNA2D1	CLEC14A	
CACNA2D2	CLIC5	
CADPS	CMTM6	
CADPS2	CNGB1	
CALN1	CROT	
CALR3	CXCL16	
CAMK1G	Cyp2c44	
CAPN3	CYP4B1	
CAPN5	CYP4X1	
CAPS	DHRS4	
CASC4	DMRTB1	
CASP16P	DMRTC2	
CATSPER2	DNAJB1	
CBL	DNAJB7	
CBLL1	DNASE2B	
CBLN4	DPEP2	
CCDC153	DSC3	
CCDC188	DUSP12	
CCDC192	DYTN	
CCDC53	DZIP1	
CCNDBP1	ECH1	
CCNH	EFHB	
CCNJL	EGFL6	
CCNO	ENTHD1	
CCT7	Epb4.1l5	
CD164L2	EPS8L3	
CDAN1	ERCC6L2	
CDC20B	ERICH5	
CDC27	EVI2A	
CDC42EP3	EVI5	
CDH10	FAM111B	
CEBPD	FAM189A1	
CELF4	FAM204A	
CEP41	FAM3D	
CEP57L1	FAM64A	
CHERP	FANCE	
CHODL	FCAR	
CHRM4	FCRL1	
CISH	FCRL3	
CKLF	FCRL5	
CKMT1A	FCRLA	
CKMT1B	FETUB	
CLASP2	FGFBP1	
CLDN10	FMO5	
CLSTN1	FOXI1	

AMH	*Gautier et al. TS3 [11]*	*Wang et al. 2017 (S14) [3]*
CMTM1	FSCN3	
CNGA3	GCC1	
CNTNAP4	GGACT	
COA5	GHRL	
COG5	GIMAP7	
COL11A1	GIMAP8	
COQ10B	GINS4	
CORO2B	GJA10	
CR2	GLOD4	
CSGALNACT2	Gm10257	
CSMD2	Gm34653	
CSPG5	Gm766	
CTDSPL2	GPLD1	
CTNNBL1	GPR50	
CTNND2	GPRIN2	
CTPS	Gramd1c	
CTXN3	GSDMC	
CXCL13	GSTA1	
CXCL3	GTF2F1	
CYB561	GZMB	
CYB561D2	HBD	
DAPP1	HEMK1	
DBIL5P2	HLA-B	
DDHD2	HLA-DMB	
DDX4	HPX	
DGCR8	HSD17B12	
DGKZ	HSD17B3	
DGUOK	HSD17B8	
DHDDS	ICAM1	
DHRS12	Ifi27	
DHX29	IFI44	
DLGAP1	IFNA16	
DNAH1	IFNAR1	
DNAJA2	IGSF23	
DNAJB4	IL10RB	
DNAJC3	IQCC	
DOCK3	KDM7A	
DPYSL5	KIAA0825	
DRAM1	KIAA1143	
DTNA	KIAA1328	
DUS4L	KIAA1551	
DUSP11	KLF5	
DUSP7	KLHDC7B	
DYNC1H1	KLK15	
DYSF	Klra2	
DZIP1	KLRC1	
E2F6	KLRD1	
EFCC1	Klrk1	
EGR4	KPRP	
EHBP1	KRT32	
EIF3J	KRT74	
ELAVL4	KRT77	
ELL3	LAG3	
ELN	LCA5	
ELP6	Lcn3	
ENTHD1	LCN8	
EPB42	LGALS14	
EPM2AIP1	LIPJ	
EPS15L1	LOC100359535	
EPSTI1	LOC100360601	
ERBB4	LRRC31	
ESCO1	LYPD8	
ESM1	MAGEB4	
EXOC6B	MALSU1	
EXTL1	MAP3K19	
FAAH	MAP7D1	
FAF2	MAVS	

AMH	*Gautier et al. TS3 [11]*	*Wang et al. 2017 (S14) [3]*
FAHD2A	MCHR2	
FAM117A	MCM10	
FAM150A	MDM1	
FAM172A	MFSD6L	
FAM177B	MICALCL	
FAM19A3	MOGAT3	
FAM46B	MRAP	
FAM49B	MRGPRD	
FAM83F	MROH8	
FAU	MRPL1	
FBXL19	MRPL16	
FBXO41	MRPS23	
FBXW7	MRPS30	
FCN3	MRPS35	
FDXACB1	MS4A3	
FERMT2	MSANTD1	
FGF12	MSRB2	
FGF14	MTG1	
FHL3	MUC15	
FHOD3	MYBPH	
FIBCD1	MYOC	
FKSG51	NAT8B	
FLJ35017	NCCRP1	
FLJ39294	NCOA1	
FLJ45513	NEIL3	
FNBP1L	NFE2L3	
FOXO1	NMRAL1	
FRMD5	NOL11	
FRMD8	NOL12	
FSTL5	NOX1	
FUBP1	NRIP2	
FUT5	NUCB1	
FXYD4	NUDT14	
FZD3	NUDT17	
GABRB3	NUPL2	
GALNT10	OCA2	
GALNT11	ODC1	
GALNT2	Olfr1178	
GALNTL5	Olfr1231	
GANC	Olfr1280	
GATA6	Olfr1353	
GBP2	Olfr1358	
GBP4	Olfr424	
GBP5	Olfr49	
GBP7	Olfr541	
GCNT2	Olfr600	
GDAP1	Olfr606	
GDF6	Olfr610	
GDPD1	Olfr711	
GGT7	Olfr794	
GINM1	Olfr867	
GK2	Olfr905	
GLI3	Olfr922	
GLT8D1	Olfr963	
GLYCTK	Olr1158	
GNAI2	Olr508	
GNAT1	Olr837	
GNL3	Olr86	
GOLGA4	OR10AG1	
GP9	OR10J5	
GPAT2	OR10K1	
GPATCH3	OR10R2	
GPM6A	OR10V1	
GPN2	OR12D3	
GPR22	OR13F1	
GPR3	OR1E2	
GPR39	OR1J1	

AMH	*Gautier et al. TS3 [11]*	*Wang et al. 2017 (S14) [3]*
GPR62	OR1L3	
GPT2	OR1M1	
GPX8	OR2AG2	
GRAP2	OR2D2	
GREB1L	OR2T12	
GRIA1	OR4C12	
GRID2	OR4C46	
GRIK3	OR4F15	
GRIK5	OR4F6	
GRM2	OR4K14	
GRM3	OR51A7	
GTDC1	OR52E8	
GTF3C5	OR52H1	
GYPA	OR52R1	
GYPB	OR5C1	
GZMA	OR5M11	
GZMK	OR6K2	
HARBI1	OR6N2	
HAUS2	OR6Y1	
HBP1	OR7G3	
HEG1	OR8A1	
HEMK1	OR9K2	
HERC5	OVCH1	
HHIP	PALM3	
HIVEP2	PAOX	
HMGB3P1	PATL2	
HMGN2	PCED1B	
HNRNPF	PDCD2L	
HRASLS2	PDHX	
HS6ST3	PER2	
HSD3B7	Pga5	
HSDL2	PGLYRP1	
HSF5	PGLYRP2	
HSPD1	PI4K2B	
HSPE1	PIGV	
HTR1E	PKD2L1	
HYAL1	PLEKHO2	
HYAL2	PLG	
HYAL3	PLIN2	
IFRD2	PLLP	
IGF1	PMVK	
IGFL2	PNLIPRP3	
IGFL3	PNMA1	
IGFL4	PNMAL1	
IL31RA	POC5	
IL6ST	POLI	
IL7	POM121L2	
INA	POU2F1	
INPP4A	PPP1R15A	
INPP5F	PRR23A	
IQCF1	PRSS16	
IQCF2	PRSS53	
IQCF3	PSTK	
IQCF5	QRICH2	
IQCF6	RAD9B	
ITFG1	RANBP3L	
ITGA9	RDH16	
ITIH1	RGL3	
ITIH3	RHBDD3	
ITIH4	RIBC1	
JMJD6	RILP	
KAT7	RITA1	
KATNA1	RNF135	
KCNA4	RNF212B	
KCND2	RSPH4A	
KCNH7	RUFY4	
KCNIP3	SAMD15	

AMH	*Gautier et al. TS3 [11]*	*Wang et al. 2017 (S14) [3]*
KCNJ3	SAMD3	
KIAA0825	SAXO2	
KIAA1143	SCAND1	
KIAA1841	SCGB2A2	
KIAA1958	SCN7A	
KIF15	Sectm1b	
KIF18A	SEMA4G	
KIFAP3	41883	
KLF2	SERPINB4	
KLHL18	SFTPC	
KMT2C	SFTPD	
LARGE1	SH2D6	
LCMT2	SHISA5	
LDHD	SIRPA	
LEMD3	SIRPB2	
LGALSL	SIX1	
LIMK1	Skint1	
LIN28A	SLC15A5	
LOH11CR1I	SLC16A11	
LPHN3	SLC16A4	
LRFN4	SLC17A1	
LRIG2	SLC17A3	
LRP1B	SLC22A10	
LRRC41	SLC22A13	
LRRC57	SLC35F4	
LRRFIP2	SLC39A4	
LSM1	SMCO2	
LSMEM2	SNAPC2	
LSR7	SNTN	
LYST	SP4	
MAGI2	SPAG5	
MAL	SPAM1	
MANF	SPATA25	
MAP1A	SPHKAP	
MAP2	SPN	
MAP3K6	SPZ1	
MAP7	ST7L	
MAPKAPK3	STRA8	
MATR3	STXBP4	
MCAM	SUPT7L	
MCHR1	SYNE4	
MCIDAS	Taar7a	
MCM4	TARS2	
MCMBP	TAS1R1	
MCTP1	TAS2R16	
MDH1	TAS2R46	
MDK	TBL2	
MDM1	TEKT2	
MED26	TEX28	
MEGF10	TF	
METTL15	THEM4	
METTL23	THPO	
MFAP1	TIGD3	
MFRP	TKTL1	
MFSD11	TMC5	
MGAT4A	TMEM223	
MIB1	TMEM260	
MIGA1	TMEM86B	
MKL1	TMPRSS11D	
MKLN1	TNFSF9	
MLH1	TRIM40	
MMP9	TRIM64C	
MOB4	TRIM77	
MPND	Tstd3	
MRPL49	UBL5	
MRPS5	UBQLNL	
MTMR4		

AMH	*Gautier et al. TS3 [11]*	*Wang et al. 2017 (S14) [3]*
MTRNR2L7	UGT2B17	
MUSTN1	ULBP3	
MYH3	UPF3A	
MYHAS	UPP2	
MYL4	USHBP1	
MYLK3	USP16	
NADK2	USP45	
NAT6	VASP	
NAT8	VGLL1	
NAV3	VPS37C	
NCOA5	VPS72	
NCOA6	Wfdc3	
NDUFA11	WISP3	
NEAT1	XAF1	
NEB	ZBTB10	
NEDD1	Zcchc13	
NEK4	ZCCHC5	
NETO2	ZGPAT	
NEXN	ZNF239	
NFG3	ZNF584	
NISCH	ZNF599	
NLK	ZNF605	
NLRX1	ZNF622	
NMUR2	ZNF674	
NOTO	ZNF683	
NPRL2	ZNHIT2	
NR0B2	ZNHIT6	
NR2F1	ZP2	
NT5DC2	ZSCAN5B	
NTM		
NTRK2		
NUDC		
NUFIP1		
NUP37		
NWD1		
NWD2		
NXPH1		
NYAP2		
ORAI3		
OTUD4		
OTX1		
PACSIN1		
PAIP2		
PARP3		
PARPBP		
PATL2		
PBRM1		
PC		
PCBP4		
PCCB		
PCDH17		
PCDH9		
PCGF6		
PCNX		
PDCD4		
PDE4B		
PDIA3		
PDZD2		
PDZD3		
PELI1		
PEX13		
PHACTR1		
PHF7		
PHKB		
PIGV		
PIK3CG		
PLA2G16		

AMH	*Gautier et al. TS3 [11]*	*Wang et al. 2017 (S14) [3]*
PLA2G4D		
PLA2G4E		
PLA2GDF		
PLAC8L1		
PLXDC2		
PMCH		
POC1A		
PODXL		
POMGNT1		
POTEC		
POU2F2		
POU3F1		
POU5F2		
PPAP2A		
PPAPDC1A		
PPAPDC1B		
PPIL4		
PPIP5K1		
PPM1E		
PPM1M		
PPP2R1B		
PRADC1		
PRDM10		
PRDM2		
PRKAR2B		
PRKCD		
PRKDC		
PROM2		
PRR11		
PSTPIP2		
PTPN23		
PTPRD		
PUS10		
PVRL3		
QSER1		
RAB11FIP5		
RAB28		
RABAC1		
RAD51C		
RAD54L		
RAD54L2		
RANBP1		
RANBP3		
RARRES3		
RASA1		
RASGEF1A		
RASSF1		
RASSF3		
RB1CC1		
RBFOX2		
RBL1		
RBM14		
RBM15B		
RBM4		
RBM4B		
RBSG3		
RCE1		
REL		
RET		
RFT1		
RFTN2		
RGS6		
RIF1		
RNF133		
RNF148		
RNF220		
RNF26		

AMH	*Gautier et al. TS3 [11]*	*Wang et al. 2017 (S14) [3]*
RNF43		
RNF44		
RNPC3		
ROBO2		
ROCK1		
RPL13AP6		
RPL29		
RPS18P9		
RPS6KA1		
RRP9		
RSPO3		
SAMHD1		
SCAP		
SCMH1		
SCYL3		
SEC23IP		
SEC24D		
SEMA3F		
SEMA3G		
SEMA6D		
SEPT4		
SERF2		
SERINC4HYPK		
SESN1		
SETD1A		
SF3A3		
SF3B1		
SFMBT1		
SFXN5		
SGMS2		
SGSM3		
SH3GL1		
SH3RF2		
SHOC2		
SIK2		
SIN3B		
SIPA1L1		
SKA2		
SKIV2L2		
SKP2		
SLC12A5		
SLC16A1		
SLC1A1		
SLC25A17		
SLC26A3		
SLC26A4		
SLC2A5		
SLC30A2		
SLC35B1		
SLC35E1		
SLC38A9		
SLC4A10		
SLC4A4		
SLC9A1		
SLFNL1		
SLIT2		
SLITRK1		
SLITRK3		
SMAD1		
SMAD9		
SMG8		
SMIM4		
SMIM7		
SMYD5		
SNAI2		
SNAP23		
SNF		

AMH	*Gautier et al. TS3 [11]*	*Wang et al. 2017 (S14) [3]*
SNHG4		
SNRPD1		
SORCS1		
SORCS2		
SORCS3		
SPATS2L		
SPCS1		
SPG11		
SPIDR		
SPOP		
SPTBN2		
SRSF2		
ST7		
STAB1		
STAC		
STAG1		
STAMBP		
STARD9		
STK3		
STMN2		
STRC		
STX1A		
STX1B		
SUPT4H1		
SYNPO2		
SYT1		
SYT6		
SYTL1		
SYVN1		
TAC4		
TAF5		
TANC2		
TAS2R16		
TBC1D23		
TBX1		
TDRD3		
TDRD7		
TEX14		
TEX264		
TFAP2D		
TGM4		
TGM5		
TGM7		
THSD7B		
THTPA		
TK2		
TKT		
TLE3		
TLR9		
TM7SF2		
TMEM110		
TMEM115		
TMEM123		
TMEM17		
TMEM222		
TMEM235		
TMEM262		
TMEM38A		
TMEM42		
TMEM62		
TMEM87A		
TMOD1		
TNFRSF21		
TNNC1		
TNRC6B		
TNS1		
TP53BP1		

AMH	*Gautier et al. TS3 [11]*	*Wang et al. 2017 (S14) [3]*
TP53INP2		
TPD52		
TPRKB		
TRANK1		
TRIM37		
TRIM43		
TRIM69		
TRIM71		
TRMT2A		
TRNP1		
TTBK2		
TTC6		
TUBGCP4		
TUSC2		
TWF2		
TYW5		
U6		
U7		
UBE2V2		
UBR1		
UGGT2		
UGP2		
UGT8		
UNC50		
UQCRH		
UQCRHL		
USP33		
USP34		
USP54		
UTP11		
VAPA		
VMAC		
VOPP1		
VPS39		
VPS51		
VPS54		
WASF2		
WBSCR22		
WDPCP		
WDR59		
WDR76		
WDR82		
WDTC1		
WHSC1L1		
WIZ		
XPO1		
ZBBX		
ZBTB20		
ZBTB34		
ZDHHC18		
ZDHHC8		
ZEB2		
ZFHX4		
ZFPL1		
ZIC4		
ZMYND10		
ZNF106		
ZNF197		
ZNF2		
ZNF205		
ZNF213		
ZNF248		
ZNF25		
ZNF33A		
ZNF33B		
ZNF35		
ZNF37A		

AMH	*Gautier et al. TS3 [11]*	*Wang et al. 2017 (S14) [3]*
ZNF407		
ZNF501		
ZNF502		
ZNF514		
ZNF521		
ZNF574		
ZNF638		
ZNF852		
ZNHIT2		
ZNRF1		
ZSCAN29		

(C) Results of hypergeometric intersection tests on data from S5 Table (A) and (B)

	wild *Canis* (grey wolf)				wild bovine (wisent)	
	Stronen et al. T2 [32]	*Stronen et al. TS3 [70]*	*Stronen et al. TS5 [33]*	*Pilot et al. TS4 [32]*	*Gautier et al. TS3 [425]*	*Wang et al. S14 [72]*
**AMH [742]**	*a* = 742, *b* = 32, v = 3; p = 0.120816	a = 742, b = 70, v = 0; p = 1	a = 742, b = 33, v = 1; p = 0.722318	*a* = 742, *b* = 32, v = 1; p = 0.7113151	*a* = 742, *b* = 425, v = 11; p = 0.9341423	*a* = 742, *b* = 72, v = 3; p = 0.5198493
