# Supplementary material for: Self-domestication in Homo sapiens: Insights from comparative genomics
Source: PLoS One. 2017 Oct 18;12(10):e0185306. doi: 10.1371/journal.pone.0185306 (PMC5646786; doi:10.1371/journal.pone.0185306)
Supplement: S6 Table — (PDF) [file pone.0185306.s007.pdf]

S6 Table. Synteny analysis of the genes overlapping between AMH and domesticated species, and those under selection in multiple domesticated species

Gene	Organism	Chromosome	NCBI Accession	Adjacent Protein-coding genes
ADAMTS13	Bos taurus (cattle)	11	AC_000168.1	ADAMTSL2, TMEM8C, SLC2A6, CACFD1, *, REXO4, STKLD1, SURF4, SURF2
	Canis lupus familiaris (dog)	9	NC_006591.3	ADAMTSL2, TMEM8C, SLC2A6, CACFD1, *, REXO4, STKLD1, SURF4, SURF2
	Equus caballus (horse)	25	NC_009168.2	DBH, FAM163B, ADAMTSL2, TMEM8C, *, REXO4, STKLD1, SURF4, SURF2
	Felis catus (domestic cat)	D4	NC_018735.2	ADAMTSL2, TMEM8C, SLC2A6, CACFD1, *, REXO4, STKLD1, SURF4, SURF2
	Homo sapiens (human)	9	NC_000009.12	ADAMTSL2, TMEM8C, SLC2A6, CACFD1, *, REXO4, STKLD1, SURF4, SURF2
AMBRA1	Bos taurus (cattle)	15	AC_000172.1	CREB3L1, DGKZ, MDK, CHRM4, *, HARBI1, ATG13, ARHGAP1, ZNF408
	Canis lupus familiaris (dog)	18	NC_006600.3	CREB3L1, DGKZ, MDK, CHRM4, *, HARBI1, ATG13, ARHGAP1, ZNF408
	Equus caballus (horse)	12	NC_009155.2	CREB3L1, DGKZ, MDK, CHRM4, *, HARBI1, ATG13, ARHGAP1, ZNF408
	Felis catus (domestic cat)	D1	NC_018732.2	CREB3L1, DGKZ, MDK, CHRM4, *, HARBI1, ATG13, ARHGAP1, ZNF408
	Homo sapiens (human)	11	NC_000011.10	CREB3L1, DGKZ, MDK, CHRM4, *, HARBI1, ATG13, ARHGAP1, ZNF408
ATXN7L1	Bos taurus (cattle)	4	AC_000161.1	KMT2E, SRPK2, PUS7, RINT1, *, CDHR3, SYPL1, NAMPT, PIK3CG
	Canis lupus familiaris (dog)	18	NC_006600.3	KMT2E, SRPK2, PUS7, RINT1, *, CDHR3, SYPL1, NAMPT, CCDC71L, PIK3CG
	Equus caballus (horse)	4	NC_009147.2	KMT2E, SRPK2, PUS7, RINT1, EFCAB10, *, CDHR3, SYPL1, NAMPT, PIK3CG
	Felis catus (domestic cat)	A2	NC_018724.2	KMT2E, SRPK2, PUS7, RINT1, *, CDHR3, SYPL1, NAMPT, PIK3CG
	Homo sapiens (human)	7	NC_000007.14	KMT2E, SRPK2, PUS7, RINT1, EFCAB10, *, CDHR3, SYPL1, NAMPT, CCDC71L, PIK3CG
BRAF	Bos taurus (cattle)	4	AC_000161.1	MKRN1, DENND2A, ADCK2, NDUFB2, *, MRPS33, TMEM178B, AGK, KIAA1147
	Canis lupus familiaris (dog)	16	NC_006598.3	MKRN1, DENND2A, ADCK2, NDUFB2, *, MRPS33, TMEM178B, AGK, KIAA1147
	Equus caballus (horse)	4	NC_009147.2	MKRN1, DENND2A, ADCK2, NDUFB2, *, MRPS33, TMEM178B, AGK, KIAA1147
	Felis catus (domestic cat)	A2	NC_018724.2	MKRN1, DENND2A, ADCK2, NDUFB2, *, MRPS33, TMEM178B, AGK, KIAA1147
	Homo sapiens (human)	7	NC_000007.14	MKRN1, DENND2A, ADCK2, NDUFB2, *, MRPS33, TMEM178B, AGK, KIAA1147
CACNA1D	Bos taurus (cattle)	22	AC_000179.1	SELK, ACTR8, IL17RB, CHDH, *, DCP1A, TKT, PRKCD, RFT1
	Canis lupus familiaris (dog)	20	NC_006602.3	SELK, ACTR8, IL17RB, CHDH, *, DCP1A, TKT, PRKCD, RFT1
	Equus caballus (horse)	16	NC_009159.2	SELK, ACTR8, IL17RB, CHDH, *, DCP1A, TKT, PRKCD, RFT1
	Felis catus (domestic cat)	A2	NC_018724.2	SELENOK[1], ACTR8, IL17RB, CHDH, *, DCP1A, TKT, PRKCD, RFT1
	Homo sapiens (human)	3	NC_000003.12	SELK, ACTR8, IL17RB, CHDH, *, DCP1A, TKT, PRKCD, RFT1
CLEC5A	Bos taurus (cattle)	4	AC_000161.1	SSBP1, TAS2R3, TAS2R4, TAS2R5, PRSS37, OR9A4, *, TAS2R38, MGAM, PRSS58, PRSS1
	Canis lupus familiaris (dog)	16	NC_006598.3	SSBP1, TAS2R3, TAS2R4, TAS2R5, PRSS37, COR9A7, *, TAS2R38, MGAM, MGAM2, PRSS58
	Equus caballus (horse)	4	NC_009147.2	SSBP1, TAS2R3, TAS2R4, PRSS37, *, TAS2R38, MGAM, MGAM2, PRSS58
	Felis catus (domestic cat)	A2	NC_018724.2	SSBP1, TAS2R3, TAS2R4, TAS2R5, PRSS37, *, TAS2R38, MGAM, MGAM2, PRSS2
	Homo sapiens (human)	7	NC_000007.14	SSBP1, TAS2R3, TAS2R4, TAS2R5, PRSS37, OR9A4, *, TAS2R38, MGAM, MGAM2, PRSS58
COA5	Bos taurus (cattle)	11	AC_000168.1	TMEM131, VWA3B, CNGA3, INPP4A, *, UNC50, MGAT4A, KIAA1211L, TSGA10
	Canis lupus familiaris (dog)	10	NC_006592.3	TMEM131, VWA3B, CNGA3, INPP4A, *, UNC50, MGAT4A, KIAA1211L, TSGA10
	Equus caballus (horse)	15	NC_009158.2	TSGA10, KIAA1211L, MGAT4A, UNC50, *, INPP4A, CNGA3, VWA3B, TMEM131
	Felis catus (domestic cat)	A3	NC_018725.2	TSGA10, KIAA1211L, MGAT4A, UNC50, [2], INPP4A, CNGA3, VWA3B, TMEM131
	Homo sapiens (human)	2	NC_000002.12	TMEM131, VWA3B, CNGA3, INPP4A, *, UNC50, MGAT4A, KIAA1211L, TSGA10
COL11A1	Bos taurus (cattle)	3	AC_000160.1	SLC30A7, DPH5, S1PR1, OLFM3, *, RNPC3, AMY2B, PRMT6, NTNG1, VAV3
	Canis lupus familiaris (dog)	6	NC_006588.3	SLC30A7, DPH5, S1PR1, OLFM3, *, RNPC3, PRMT6, NTNG1, VAV3
	Equus caballus (horse)	5	NC_009148.2	SLC30A7, DPH5, S1PR1, OLFM3, *, RNPC3, PRMT6, NTNG1, VAV3
	Felis catus (domestic cat)	C1	NC_018730.2	SLC30A7, DPH5, S1PR1, OLFM3, *, RNPC3, PRMT6, NTNG1, VAV3
	Homo sapiens (human)	1	NC_000001.11	SLC30A7, DPH5, S1PR1, OLFM3, *, RNPC3, AMY2B, AMY2A, AMY1A, AMY1B, AMY1C, PRMT6, NTNG1, VAV3
COQ10B	Bos taurus (cattle)	2	AC_000159.1	RFTN2, MOB4, HSPE1, HSPD1, *, SF3B1, ANKRD44, PGAP1, C2H2orf66
	Canis lupus familiaris (dog)	37	NC_006619.3	RFTN2, MOB4, HSPE1, HSPD1, *, SF3B1, ANKRD44, PGAP1, C37H2orf66
	Equus caballus (horse)	18	NC_009161.2	RFTN2, HSPE1-MOB4, HSPE1, HSPD1, *, SF3B1, ANKRD44, PGAP1, C18H2orf66
	Felis catus (domestic cat)	C1	NC_018730.2	MARS2, RFTN2, HSPE1, HSPD1, *, SF3B1, ANKRD44, PGAP1, GTF3C3
	Homo sapiens (human)	2	NC_000002.12	RFTN2, MOB4, HSPE1, HSPD1, *, SF3B1, ANKRD44, PGAP1, C2orf66
DCC	Bos taurus (cattle)	24	AC_000181.1	C24H18orf54, STARD6, POLI, MBD2, *, MEX3C, SMAD4, ELAC1, ME2
	Canis lupus familiaris (dog)	1	NC_006583.3	C1H18orf54, STARD6, POLI, MBD2, *, MEX3C, SMAD4, ELAC1, ME2
	Equus caballus (horse)	8	NC_009151.2	C8H18orf54, STARD6, POLI, MBD2, *, MEX3C, SMAD4, ELAC1, ME2
	Felis catus (domestic cat)	D3	NC_018734.2	CD3H18orf54, STARD6, POLI, MBD2, *, MEX3C, SMAD4, ELAC1, ME2
	Homo sapiens (human)	18	NC_000018.10	C18orf54, STARD6, POLI, MBD2, *, MEX3C, SMAD4, ELAC1, ME2
DLGAP1	Bos taurus (cattle)	24	AC_000181.1	MYOM1, MYL12A, MYL12B, TGIF1, *, C24H18orf42, ZBTB14, EPB41L3, TMEM200C
	Canis lupus familiaris (dog)	7	NC_006589.3	LPIN2, MYOM1, MYL12B, TGIF1, *, C7H18orf42, ZBTB14, EPB41L3, L3MBTL4
	Equus caballus (horse)	8	NC_009151.2	LPIN2, MYOM1, MYL12A, TGIF1, *, C8H18orf42, ZBTB14, LAMA3, TTC39C
	Felis catus (domestic cat)	D3	NC_018734.2	EMILIN2, LPIN2, MYOM1, TGIF1, *, AKAIN1[3], ZBTB14, EPB41L3, TMEM200C
	Homo sapiens (human)	18	NC_000018.10	MYOM1, MYL12A, MYL12B, TGIF1, *, AKAIN1[3], ZBTB14, EPB41L3, TMEM200C
ERBB4	Bos taurus (cattle)	2	AC_000159.1	ACADL, MYL1, LANCL1, CPS1, *, IKZF2, SPAG16, VWC2L, BARD1
	Canis lupus familiaris (dog)	37	NC_006619.3	ACADL, MYL1, LANCL1, CPS1, *, IKZF2, SPAG16, VWC2L, BARD1
	Equus caballus (horse)	6	NC_009149.2	ACADL, MYL1, LANCL1, CPS1, *, IKZF2, SPAG16, VWC2L, BARD1
	Felis catus (domestic cat)	C1	NC_018730.2	ACADL, MYL1, LANCL1, CPS1, *, IKZF2, SPAG16, VWC2L, BARD1
	Homo sapiens (human)	2	NC_000002.12	ACADL, MYL1, LANCL1, CPS1, *, IKZF2, SPAG16, VWC2L, BARD1
FAM172A	Bos taurus (cattle)	7	AC_000164.1	ADGRV1, ARRDC3, NR2F1, POU5F2, *, KIAA0825, SLF1, MCTP1, FAM81B
	Canis lupus familiaris (dog)	3	NC_006585.3	LYSMD3, ADGRV1, ARRDC3, NR2F1, *, KIAA0825, SLF1, MCTP1, FAM81B
	Equus caballus (horse)	14	NC_009157.2	ADGRV1, ARRDC3, NR2F1, POU5F2, *, KIAA0825, SLF1, MCTP1, FAM81B
	Felis catus (domestic cat)	A1	NC_018723.2	ADGRV1, ARRDC3, NR2F1, POU5F2, *, KIAA0825, SLF1, MCTP1, FAM81B
	Homo sapiens (human)	5	NC_000005.10	ADGRV1, ARRDC3, NR2F1, POU5F2, *, KIAA0825, SLF1, MCTP1, FAM81B
GGT7	Bos taurus (cattle)	13	AC_000170.1	MAP1LC3A, PIGU, TP53INP2, NCOA6, *, ACSS2, GSS, MYH7B, TRPC4AP
	Canis lupus familiaris (dog)	24	NC_006606.3	MAP1LC3A, PIGU, TP53INP2, NCOA6, *, ACSS2, GSS, MYH7B, TRPC4AP
	Equus caballus (horse)	22	NC_009165.2	MAP1LC3A, PIGU, TP53INP2, NCOA6, *, ACSS2, GSS, MYH7B, TRPC4AP
	Felis catus (domestic cat)	A3	NC_018725.2	MAP1LC3A, PIGU, TP53INP2, NCOA6, *, ACSS2, GSS, MYH7B, TRPC4AP
	Homo sapiens (human)	20	NC_000020.11	MAP1LC3A, PIGU, TP53INP2, NCOA6, *, ACSS2, GSS, MYH7B, TRPC4AP
GRIA1	Bos taurus (cattle)	7	AC_000164.1	SAP30L, HAND1, GALNT10, SMIM15, MFAP3, FAM114A2, *, NMUR2, GLRA1, G3BP1, ATOX1
	Canis lupus familiaris (dog)	4	NC_006586.3	SAP30L, GALNT10, MFAP3, FAM114A2, *, NMUR2, GLRA1, G3BP1, ATOX1
	Equus caballus (horse)	14	NC_009157.2	SAP30L, GALNT10, MFAP3, FAM114A2, *, NMUR2, GLRA1, G3BP1, ATOX1
	Felis catus (domestic cat)	A1	NC_018723.2	SAP30L, GALNT10, MFAP3, FAM114A2, *, NMUR2, GLRA1, G3BP1, ATOX1
	Homo sapiens (human)	5	NC_000005.10	SAP30L, GALNT10, MFAP3, FAM114A2, *, NMUR2, GLRA1, G3BP1, ATOX1
GRIK3	Bos taurus (cattle)	3	AC_000160.1	LSM10, OSCP1, MRPS15, CSF3R, *, ZC3H12A, MEAF6, SNIP1, DNALI1
	Canis lupus familiaris (dog)	15	NC_006597.3	LSM10, OSCP1, MRPS15, CSF3R, *, ZC3H12A, MEAF6, SNIP1, DNALI1
	Equus caballus (horse)	2	NC_009145.2	LSM10, OSCP1, MRPS15, CSF3R, *, ZC3H12A, MEAF6, SNIP1, DNALI1
	Felis catus (domestic cat)	C1	NC_018730.2	LSM10, OSCP1, MRPS15, CSF3R, *, ZC3H12A, MEAF6, SNIP1, DNALI1

Gene	Organism	Chromosome	NCBI Accession	Adjacent Protein-coding genes
	Homo sapiens (human)	1	NC_000001.11	LSM10, OSCP1, MRPS15, CSF3R, *, ZC3H12A, MEAF6, SNIP1, DNALI1
HSD3B7	Bos taurus (cattle)	25	AC_000182.1	ZNF646, ZNF668, STX4, STX1B, *, SETD1A, ORAI3, FBXL19, CTF1
	Canis lupus familiaris (dog)	6	NC_006588.3	ZNF646, ZNF668, STX4, STX1B, *, SETD1A, ORAI3, FBXL19, CTF1
	Equus caballus (horse)	13	NC_009156.2	ZNF646, ZNF668, STX4, STX1B, *, SETD1A, ORAI3, FBXL19, CTF1
	Felis catus (domestic cat)	E3	NC_018738.2	ZNF646, ZNF668, STX4, STX1B, *, SETD1A, ORAI3, FBXL19, BCL7C
	Homo sapiens (human)	16	NC_000016.10	ZNF646, ZNF668, STX4, STX1B, *, SETD1A, ORAI3, FBXL19, CTF1
HSPD1	Bos taurus (cattle)	2	AC_000159.1	PGAP1, ANKRD44, SF3B1, COQ10B, *, HSPE1, MOB4, RFTN2, MARS2
	Canis lupus familiaris (dog)	37	NC_006619.3	PGAP1, ANKRD44, SF3B1, COQ10B, *, HSPE1, MOB4, RFTN2, MARS2
	Equus caballus (horse)	18	NC_009161.2	PGAP1, ANKRD44, SF3B1, COQ10B, *, HSPE1, HSPE1-MOB4, RFTN2, MARS2
	Felis catus (domestic cat)	C1	NC_018730.2	PGAP1, ANKRD44, SF3B1, COQ10B, *, HSPE1, RFTN2, MARS2, BOLL
	Homo sapiens (human)	2	NC_000002.12	PGAP1, ANKRD44, SF3B1, COQ10B, *, HSPE1, MOB4, RFTN2, MARS2
HSPE1	Bos taurus (cattle)	2	AC_000159.1	BOLL, MARS2, RFTN2, MOB4, *, HSPD1, COQ10B, SF3B1, ANKRD44
	Canis lupus familiaris (dog)	37	NC_006619.3	BOLL, MARS2, RFTN2, MOB4, *, HSPD1, COQ10B, SF3B1, ANKRD44
	Equus caballus (horse)	18	NC_009161.2	BOLL, MARS2, RFTN2, HSPE1-MOB4, *, HSPD1, COQ10B, SF3B1, ANKRD44
	Felis catus (domestic cat)	C1	NC_018730.2	PLCL1, BOLL, MARS2, RFTN2, *, HSPD1, COQ10B, SF3B1, ANKRD44
	Homo sapiens (human)	2	NC_000002.12	BOLL, MARS2, RFTN2, MOB4, *, HSPD1, COQ10B, SF3B1, ANKRD44
ITGA9	Bos taurus (cattle)	22	AC_000179.1	DLEC1, PLCD1, VILL, CTDSPL, *, C22H3orf35, GOLGA4, DCLK3, TRANK1, EPM2AIP1, MLH1, LRRFIP2
	Canis lupus familiaris (dog)	23	NC_006605.3	DLEC1, PLCD1, VILL, CTDSPL, *, C23H3orf35, GOLGA4, LRRFIP2, MLH1, EPM2AIP1, TRANK1, DCLK3
	Equus caballus (horse)	16	NC_009159.2	DLEC1, PLCD1, VILL, CTDSPL, *, C16H3orf35, GOLGA4, LRRFIP2, MLH1, EPM2AIP1, TRANK1, DCLK3
	Felis catus (domestic cat)	C2	NC_018731.2	DLEC1, PLCD1, VILL, CTDSPL, *, CC2H3orf35, GOLGA4, LRRFIP2, MLH1, EPM2AIP1, TRANK1, DCLK3
	Homo sapiens (human)	3	NC_000003.12	DLEC1, PLCD1, VILL, CTDSPL, *, C3orf35, GOLGA4, LRRFIP2, MLH1, EPM2AIP1, TRANK1, DCLK3
LRP1B	Bos taurus (cattle)	2	AC_000159.1	THSD7B, HNMT, SPOPL, NXPH2, *, KYNU, ARHGAP15, GTDC1, ZEB2
	Canis lupus familiaris (dog)	19	NC_006601.3	THSD7B, HNMT, SPOPL, NXPH2, *, KYNU, ARHGAP15, GTDC1, ZEB2
	Equus caballus (horse)	18	NC_009161.2	THSD7B, HNMT, SPOPL, NXPH2, [4], KYNU, ARHGAP15, GTDC1, ZEB2
	Felis catus (domestic cat)	C1	NC_018730.2	THSD7B, HNMT, SPOPL, NXPH2, *, KYNU, ARHGAP15, GTDC1, ZEB2
	Homo sapiens (human)	2	NC_000002.12	THSD7B, HNMT, SPOPL, NXPH2, *, KYNU, ARHGAP15, GTDC1, ZEB2
LYST	Bos taurus (cattle)	28	AC_000185.1	GGPS1, TBCE, B3GALNT2, GNG4, *, NID1, GPR137B, ERO1B, EDARADD
	Canis lupus familiaris (dog)	4	NC_006586.3	GGPS1, TBCE, B3GALNT2, GNG4, *, NID1, GPR137B, ERO1B, EDARADD
	Equus caballus (horse)	1	NC_009144.2	GGPS1, TBCE, B3GALNT2, GNG4, *, NID1, GPR137B, ERO1B, EDARADD
	Felis catus (domestic cat)	D2	NC_018733.2	GGPS1, TBCE, B3GALNT2, GNG4, *, NID1, GPR137B, ERO1B, EDARADD
	Homo sapiens (human)	1	NC_000001.11	GGPS1, TBCE, B3GALNT2, GNG4, *, NID1, GPR137B, ERO1B, EDARADD
MOB4	Bos taurus (cattle)	2	AC_000159.1	PLCL1, BOLL, MARS2, RFTN2, *, HSPE1, HSPD1, COQ10B, SF3B1
	Canis lupus familiaris (dog)	37	NC_006619.3	PLCL1, BOLL, MARS2, RFTN2, *, HSPE1, HSPD1, COQ10B, SF3B1
	Equus caballus (horse)	18	NC_009161.2	PLCL1, BOLL, MARS2, RFTN2, [5], HSPE1-MOB4, HSPE1, HSPD1, COQ10B, SF3B1
	Felis catus (domestic cat)	C1	NT_289498.1	PLCL1, BOLL, MARS2, RFTN2, [6], HSPE1, HSPD1, COQ10B, SF3B1
	Homo sapiens (human)	2	NC_000002.12	PLCL1, BOLL, MARS2, RFTN2, *, HSPE1, HSPD1, COQ10B, SF3B1
MYLK3	Bos taurus (cattle)	18	AC_000175.1	GAS8, SHCBP1, VPS35, ORC6, *, C18H16orf87, GPT2, DNAJA2, NETO2
	Canis lupus familiaris (dog)	15	NC_006597.3	ZYG11B, SHCBP1, VPS35, ORC6, *, C22H16orf87, GPT2, CSMD2, HMGB4
	Equus caballus (horse)	3	NC_009146.2	SHCBP1, VPS35, ORC6, *, C16orf87, GPT2, DNAJA2, NETO2
	Felis catus (domestic cat)	E2	NC_018737.2	VSTM2B, SHCBP1, VPS35, ORC6, *, CE2H16orf87, GPT2, DNAJA2, NETO2
	Homo sapiens (human)	16	NC_000016.10	SHCBP1, VPS35, ORC6, *, C16orf87, GPT2, DNAJA2, NETO2
NCOA6	Bos taurus (cattle)	13	AC_000170.1	DYNLRB1, MAP1LC3A, PIGU, TP53INP2, *, GGT7, ACSS2, GSS, MYH7B
	Canis lupus familiaris (dog)	24	NC_006606.3	DYNLRB1, MAP1LC3A, PIGU, TP53INP2, *, GGT7, ACSS2, GSS, MYH7B
	Equus caballus (horse)	22	NC_009165.2	DYNLRB1, MAP1LC3A, PIGU, TP53INP2, *, GGT7, ACSS2, GSS, MYH7B
	Felis catus (domestic cat)	A3	NC_018725.2	DYNLRB1, MAP1LC3A, PIGU, TP53INP2, *, GGT7, ACSS2, GSS, MYH7B
	Homo sapiens (human)	20	NC_000020.11	DYNLRB1, MAP1LC3A, PIGU, TP53INP2, *, GGT7, ACSS2, GSS, MYH7B
NEK4	Bos taurus (cattle)	22	AC_000179.1	PBRM1, GNL3, GLT8D1, SPCS1, *, ITIH1, ITIH3, ITIH4, MUSTN1, TMEM110, SFMBT1
	Canis lupus familiaris (dog)	20	NC_006602.3	PBRM1, GNL3, GLT8D1, SPCS1, *, ITIH1, SFMBT1, RFT1, PRKCD
	Equus caballus (horse)	16	NC_009159.2	PBRM2, GNL3, GLT8D1, SPCS1, *, ITIH1, ITIH3, ITIH4, SFMBT1
	Felis catus (domestic cat)	A2	NC_018724.2	PBRM1, GNL3, GLT8D1, SPCS1, *, ITIH1, ITIH3, ITIH4, MUSTN1, TMEM110, SFMBT1
	Homo sapiens (human)	3	NC_000003.12	PBRM1, GNL3, GLT8D1, SPCS1, *, ITIH1, ITIH3, ITIH4, MUSTN1, TMEM110-MUSTN1, TMEM110, SFMBT1
NRG2	Bos taurus (cattle)	7	AC_000164.1	TMEM173, UBE2D2, CXXC5, PSD2, *, PURA, IGIP, CYSTM1, PFDN1
	Canis lupus familiaris (dog)	2	NC_006584.3	TMEM173, UBE2D2, CXXC5, PSD2, *, PURA, IGIP, CYSTM1, PFDN1
	Equus caballus (horse)	14	NC_009157.2	TMEM173, UBE2D2, CXXC5, PSD2, *, IGIP, CYSTM1, PFDN1, HBEGF
	Felis catus (domestic cat)	A1	NC_018723.2	TMEM173, UBE2D2, CXXC5, PSD2, *, PURA, IGIP, CYSTM1, PFDN1
	Homo sapiens (human)	5	NC_000005.10	TMEM173, UBE2D2, CXXC5, PSD2, *, PURA, IGIP, CYSTM1, PFDN1
NT5DC2	Bos taurus (cattle)	22	AC_000179.1	SEMA3G, TNNC1, NISCH, STAB1, *, SMIM4, PBRM1, GNL3, GLT8D1
	Canis lupus familiaris (dog)	20	NC_006602.3	SEMA3G, TNNC1, NISCH, STAB1, *, SMIM4, PBRM1, GNL3, GLT8D1
	Equus caballus (horse)	16	NC_009159.2	SEMA3G, TNNC1, NISCH, STAB1, *, SMIM4, PBRM1, GNL3, GLT8D1
	Felis catus (domestic cat)	A2	NC_018724.2	SEMA3G, TNNC1, NISCH, STAB1, *, SMIM4, PBRM1, GNL3, GLT8D1
	Homo sapiens (human)	3	NC_000003.12	SEMA3G, TNNC1, NISCH, STAB1, *, SMIM4, PBRM1, GNL3, GLT8D1
NTM	Bos taurus (cattle)	29	AC_000186.1	JAM3, IGSF9B, SPATA19, HIST1H4D, OPCML, *, TMEM45B, NFRKB, PRDM10, APLP2, ST14, ZBTB44, ADAMTS8, ADAMT[illegible]
	Canis lupus familiaris (dog)	5	NC_006587.3	JAM3, IGSF9B, SPATA19, OPCML, *, SNX19, ADAMTS15, ADAMTS8, ZBTB44, ST14, APLP2, PRDM10, NFRKB, TMEM45[illegible]
	Equus caballus (horse)	7	NC_009150.2	JAM3, IGSF9B, SPATA19, OPCML, *, SNX19, ADAMTS15, ADAMTS8, ZBTB44, ST14, APLP2, PRDM10, NFRKB, TMEM45[illegible]
	Felis catus (domestic cat)	D1	NC_018732.2	JAM3, IGSF9B, SPATA19, OPCML, *, SNX19, ADAMTS15, ADAMTS8, ZBTB44, ST14, APLP2, PRDM10, NFRKB, TMEM45[illegible]
	Homo sapiens (human)	11	NC_000011.10	JAM3, IGSF9B, SPATA19, OPCML, *, SNX19, C11orf44, ADAMTS15, ADAMTS8, ZBTB44, ST14, APLP2, PRDM10, NFRKB, [illegible]
PLAC8L1	Bos taurus (cattle)	7	AC_000164.1	KCTD16, PRELID2, GRXCR2, SH3RF2, *, LARS, RBM27, POU4F3, TCERG1
	Canis lupus familiaris (dog)	2	NC_006584.3	KCTD16, PRELID2, GRXCR2, SH3RF2, *, LARS, RBM27, POU4F3, TCERG1
	Equus caballus (horse)	14	NC_009157.2	KCTD16, PRELID2, GRXCR2, SH3RF2, *, LARS, RBM27, POU4F3, TCERG1
	Felis catus (domestic cat)	A1	NC_018723.2	KCTD16, PRELID2, GRXCR2, SH3RF2, *, LARS, RBM27, POU4F3, TCERG1
	Homo sapiens (human)	5	NC_000005.10	KCTD16, PRELID2, GRXCR2, SH3RF2, *, LARS, RBM27, POU4F3, TCERG1
PPAP2A	Bos taurus (cattle)	20	AC_000177.1	MCIDAS, CCNO, DHX29, SKIV2L2, *, SLC38A9, DDX4 IL31RA, IL6ST
(=PLPP1)	Canis lupus familiaris (dog)	2	NC_006584.3	MCIDAS, CCNO, DHX29, SKIV2L2, *, SLC38A9, DDX4 IL31RA, IL6ST
	Equus caballus (horse)	21	NC_009164.2	MCIDAS, CCNO, DHX29, SKIV2L2, *, SLC38A9, DDX4 IL31RA, IL6ST
	Felis catus (domestic cat)	A1	NC_018723.2	MCIDAS, CCNO, DHX29, SKIV2L2, *, SLC38A9, DDX4 IL31RA, IL6ST
	Homo sapiens (human)	5	NC_000005.10	MCIDAS, CCNO, DHX29, SKIV2L2, *, SLC38A9, DDX4 IL31RA, IL6ST
PPAPDC1B	Bos taurus (cattle)	27	AC_000184.1	EIF4EBP1, ASH2L, STAR, LSM1, *, BAG4, WHSC1L1, DDHD2, LETM2, FGFR1, TACC1
(=PLPP5)	Canis lupus familiaris (dog)	16	NC_006598.3	EIF4EBP1, ASH2L, STAR, LSM1, BAG4, DDHD2, *, WHSC1L1, LETM2, FGFR1, TACC1
	Equus caballus (horse)	16	NC_006598.3	EIF4EBP1, ASH2L, STAR, LSM1, BAG4, DDHD2, *, WHSC1L1, LETM2, FGFR1, C27H8orf86, TACC1
	Felis catus (domestic cat)	B1	NC_018726.2	EIF4EBP1, ASH2L, STAR, LSM1, BAG4, DDHD2, *, NSD3[7], LETM2, FGFR1, TACC1
	Homo sapiens (human)	8	NC_000008.11	EIF4EBP1, ASH2L, STAR, LSM1, BAG4, DDHD2, *, WHSC1L1, LETM2, FGFR1, C8orf86, TACC1

Gene	Organism	Chromosome	NCBI Accession	Adjacent Protein-coding genes
PRR11	Bos taurus (cattle)	19	AC_000176.1	DHX40, YPEL2, GDPD1, SMG8, *, SKA2, TRIM37, PPM1E, RAD51C
	Canis lupus familiaris (dog)	9	NC_006591.3	DHX40, YPEL2, GDPD1, SMG8, *, SKA2, TRIM37, PPM1E, RAD51C
	Equus caballus (horse)	11	NC_009154.2	DHX40, YPEL2, GDPD1, SMG8, *, SKA2, TRIM37, PPM1E, RAD51C
	Felis catus (domestic cat)	E1	NC_018736.2	DHX40, YPEL2, GDPD1, SMG8, *, SKA2, TRIM37, PPM1E, RAD51C
	Homo sapiens (human)	17	NC_000017.11	DHX40, YPEL2, GDPD1, SMG8, *, SKA2, TRIM37, PPM1E, RAD51C
PVRL3	Bos taurus (cattle)	1	AC_000158.1	PHLDB2, PLCXD2, ZBED2, CD96, *, DPPA4, DPPA2, MORC1, GUCA1C
(=NECTIN3)	Canis lupus familiaris (dog)	33	NC_006615.3	PHLDB2, PLCXD2, ZBED2, CD96, *, DPPA4, DPPA2, MORC1, GUCA1C
	Equus caballus (horse)	19	NC_009162.2	PHLDB2, EIF3F, PLCXD2, ZBED2, CD96, *, DPPA4, DPPA2, MORC1, GUCA1C
	Felis catus (domestic cat)	C2	NC_018731.2	PHLDB2, PLCXD2, ZBED2, CD96, *, DPPA4, DPPA2, MORC1, GUCA1C
	Homo sapiens (human)	3	NC_000003.12	PHLDB2, PLCXD2, ZBED2, CD96, *, DPPA4, DPPA2, FLJ22763, MORC1, GUCA1C
RFTN2	Bos taurus (cattle)	2	AC_000159.1	COQ10B, HSPD1, HSPE1, MOB4, *, MARS2, BOLL, PLCL1, SATB2
	Canis lupus familiaris (dog)	37	NC_006619.3	COQ10B, HSPD1, HSPE1, MOB4, *, MARS2, BOLL, PLCL1, SATB2
	Equus caballus (horse)	18	NC_009161.2	COQ10B, HSPD1, HSPE1, HSPE1-MOB4, *, MARS2, BOLL, PLCL1, SATB2
	Felis catus (domestic cat)	C1	NC_018730.2	SF3B1, COQ10B, HSPD1, HSPE1, *, MARS2, BOLL, PLCL1, SATB2
	Homo sapiens (human)	2	NC_000002.12	COQ10B, HSPD1, HSPE1, MOB4, *, MARS2, BOLL, PLCL1, SATB2
RNPC3	Bos taurus (cattle)	3	AC_000160.1	SLC25A24, VAV3, NTNG1, PRMT6, AMY2B, *, COL11A1, OLFM3, S1PR1, DPH5
	Canis lupus familiaris (dog)	6	NC_006588.3	SLC25A24, VAV3, NTNG1, PRMT6, *, COL11A1, OLFM3, S1PR1, DPH5
	Equus caballus (horse)	5	NC_009148.2	SLC25A24, VAV3, NTNG1, PRMT6, *, COL11A1, OLFM3, S1PR1, DPH5
	Felis catus (domestic cat)	C1	NC_018730.2	SLC25A24, VAV3, NTNG1, PRMT6, *, COL11A1, OLFM3, S1PR1, DPH5
	Homo sapiens (human)	1	NC_000001.11	SLC25A24, VAV3, NTNG1, PRMT6, AMY1C, AMY1B, AMY1A, AMY2A, AMY2B, *, COL11A1, OLFM3, S1PR1, DPH5
SEC24A	Bos taurus (cattle)	7	AC_000164.1	TXNDC15, C7H5orf24, DDX46, CAMLG, *, SAR1B, JADE2, CDKN2AIPNL, UBE2B
	Canis lupus familiaris (dog)	11	NC_006593.3	TXNDC15, C11H5orf24, DDX46, CAMLG, *, SAR1B, JADE2, CDKN2AIPNL, UBE2B
	Equus caballus (horse)	14	NC_009157.2	TXNDC15, C14H5orf24, DDX46, CAMLG, *, SAR1B, JADE2, CDKN2AIPNL, UBE2B
	Felis catus (domestic cat)	A1	NC_018723.2	TXNDC15, CA1H5orf24, DDX46, CAMLG, *, SAR1B, JADE2, CDKN2AIPNL, UBE2B
	Homo sapiens (human)	5	NC_000005.10	TXNDC15, C5orf24, DDX46, CAMLG, *, SAR1B, JADE2, CDKN2AIPNL, UBE2B
SF3B1	Bos taurus (cattle)	2	AC_000159.1	GTF3C3, C2H2orf66, PGAP1, ANKRD44, *, COQ10B, HSPD1, HSPE1, MOB4
	Canis lupus familiaris (dog)	37	NC_006619.3	GTF3C3, C37H2orf66, PGAP1, ANKRD44, *, COQ10B, HSPD1, HSPE1, MOB4
	Equus caballus (horse)	18	NC_009161.2	GTF3C3, C18H2orf66, PGAP1, ANKRD44, *, COQ10B, HSPD1, HSPE1, HSPE1-MOB4
	Felis catus (domestic cat)	C1	NC_018730.2	CCDC150, GTF3C3, PGAP1, ANKRD44, *, COQ10B, HSPD1, HSPE1, RFTN2
	Homo sapiens (human)	2	NC_000002.12	GTF3C3, C2orf66, PGAP1, ANKRD44, *, COQ10B, HSPD1, HSPE1, MOB4
SKA2	Bos taurus (cattle)	19	AC_000176.1	TEX14, RAD51C, PPM1E, TRIM37, *, PRR11, SMG8, GDPD1, YPEL2
	Canis lupus familiaris (dog)	9	NC_006591.3	TEX14, RAD51C, PPM1E, TRIM37, *, PRR11, SMG8, GDPD1, YPEL2
	Equus caballus (horse)	11	NC_009154.2	TEX14, RAD51C, PPM1E, TRIM37, *, PRR11, SMG8, GDPD1, YPEL2
	Felis catus (domestic cat)	E1	NC_018736.2	TEX14, RAD51C, PPM1E, TRIM37, *, PRR11, SMG8, GDPD1, YPEL2
	Homo sapiens (human)	17	NC_000017.11	TEX14, RAD51C, PPM1E, TRIM37, *, PRR11, SMG8, GDPD1, YPEL2
SMG6	Bos taurus (cattle)	19	AC_000176.1	RTN4RL1, DPH1, OVCA2, HIC1, *, SRR, TSR1, SGSM2, MNT
	Canis lupus familiaris (dog)	9	NC_006591.3	RTN4RL1, DPH1, OVCA2, HIC1, *, SRR, SGSM2, TSR1, MNT
	Equus caballus (horse)	11	NC_009154.2	RTN4RL1, DPH1, OVCA2, HIC1, *, SRR, TSR1, SGSM2, MNT
	Felis catus (domestic cat)	E1	NC_018736.2	RTN4RL1, DPH1, OVCA2, HIC1, *, SRR, TSR1, SGSM2, MNT
	Homo sapiens (human)	17	NC_000017.11	RTN3RL1, DPH1, OVCA2, HIC1, *, SRR, TSR1, SGSM2, MNT
SNRPD1	Bos taurus (cattle)	24	AC_000181.1	RBBP8, GATA6, MIB1, ABHD3, *, ESCO1, GREB1L, ROCK1, USP14
	Canis lupus familiaris (dog)	7	NC_006589.3	RBBP8, GATA6, MIB1, ABHD3, *, ESCO1, GREB1L, ROCK1, USP14
	Equus caballus (horse)	8	NC_009151.2	RBBP8, GATA6, MIB1, ABHD3, *, ESCO1, GREB1L, ROCK1, USP14
	Felis catus (domestic cat)	D3	NC_018734.2	RBBP8, GATA6, MIB1, ABHD3, *, ESCO1, GREB1L, ROCK1, USP14
	Homo sapiens (human)	18	NC_000018.10	RBBP8, CTAGE1, GATA6, MIB1, ABHD3, *, ESCO1, GREB1L, ROCK1
STAB1	Bos taurus (cattle)	22	AC_000179.1	GNL3, PBRM1, SMIM4, NT5DC2, *, NISCH, TNNC1, SEMA3G, PHF7
	Canis lupus familiaris (dog)	20	NC_006602.3	GNL3, PBRM1, SMIM4, NT5DC2, *, NISCH, TNNC1, SEMA3G, PHF7
	Equus caballus (horse)	16	NC_009159.2	GNL3, PBRM1, SMIM4, NT5DC2, *, NISCH, TNNC1, SEMA3G, PHF7
	Felis catus (domestic cat)	A2	NC_018724.2	GNL3, PBRM1, SMIM4, NT5DC2, *, NISCH, TNNC1, SEMA3G, PHF7
	Homo sapiens (human)	3	NC_000003.12	GNL3, PBRM1, SMIM4, NT5DC2, *, NISCH, TNNC1, SEMA3G, PHF7
STK10	Bos taurus (cattle)	20	AC_000177.1	NPM1, FGF18, SMIM23, FBXW11, *, EFCAB9, UBTD2, SH3PXD2B, NEURL1B
	Canis lupus familiaris (dog)	4	NC_006586.3	NPM1, FGF18, SMIM23, FBXW11, *, EFCAB9, UBTD2, SH3PXD2B, NEURL1B
	Equus caballus (horse)	14	NC_009157.2	NPM1, FGF18, SMIM23, FBXW11, *, EFCAB9, UBTD2, SH3PXD2B, NEURL1B
	Felis catus (domestic cat)	A1	NC_018723.2	NPM1, FGF18, SMIM23, FBXW11, *, EFCAB9, UBTD2, SH3PXD2B, NEURL1B
	Homo sapiens (human)	5	NC_018723.2	NPM1, FGF18, SMIM23, FBXW11, *, EFCAB9, UBTD2, SH3PXD2B, NEURL1B
SYTL1	Bos taurus (cattle)	2	AC_000159.1	GPR3, CD164L2, FCN3, MAP3K6, *, WDTC1, TMEM222, SLC9A1, FAM46B
	Canis lupus familiaris (dog)	2	NC_006584.3	GPR3, CD164L2, FCN3, MAP3K6, *, TMEM222, WDTC1, SLC9A1, FAM46B
	Equus caballus (horse)	2	NC_009145.2	GPR3, CD164L2, FCN3, MAP3K6, *, TMEM222, WDTC1, SLC9A1, FAM46B
	Felis catus (domestic cat)	C1	NC_018730.2	GPR3, CD164L2, FCN3, MAP3K6, *, TMEM222, WDTC1, SLC9A1, FAM46B
	Homo sapiens (human)	2	NC_009145.2	GPR3, CD164L2, FCN3, MAP3K6, *, TMEM222, WDTC1, SLC9A1, FAM46B
TAS2R16	Bos taurus (cattle)	4	AC_000161.1	FEZF1, RNF133, RNF148, CADPS2, *, SLC13A1, IQUB, NDUFA5, ABS15
	Canis lupus familiaris (dog)	14	NC_006596.3	FEZF1, RNF133, RNF148, CADPS2, [8], SLC13A1, IQUB
	Equus caballus (horse)	4	NC_009147.2	FEZF1, RNF133, RNF148, CADPS2, *, SLC13A1, IQUB, NDUFA5, ABS15
	Felis catus (domestic cat)	A2	NC_018724.2	FEZF1, RNF133, RNF148, CADPS2, *, SLC13A1, IQUB, NDUFA5, ASB15
	Homo sapiens (human)	7	NC_000007.14	FEZF1, RNF133, RNF148, CADPS2, *, SLC13A1, IQUB, NDUFA5, ASB15
TEX14	Bos taurus (cattle)	19	AC_000176.1	RNF43, HSF5, MTMR4, SEPT4, C19H17orf47, *, RAD51C PPM1E, TRIM37, SKA2
	Canis lupus familiaris (dog)	9	NC_006591.3	RNF43, HSF5, MTMR4, *, RAD51C, PPM1E, TRIM37, SKA2
	Equus caballus (horse)	11	NC_009154.2	RNF43, HSF5, MTMR4, SEPT4, C17orf47, *, RAD51C, PPM1E, TRIM37, SKA2
	Felis catus (domestic cat)	E1	NC_018736.2	RNF43, HSF5, MTMR4, *, RAD51C, PPM1E, TRIM37, SKA2
	Homo sapiens (human)	17	NC_000017.11	RNF43, HSF5, MTMR4, SEPT4, C17orf47, * RAD51C, PPM1E, TRIM37, SKA2
TMEM132D	Bos taurus (cattle)	17	AC_000174.1	PRAME, TMEM132C, SLC15A4, GLT1D1, *, FZD10, PIWIL1, RIMBP2, STX2
	Canis lupus familiaris (dog)	26	NC_006608.3	TMEM132B, TMEM132C, SLC15A4, GLT1D1, *, FZD10, PIWIL1, RIMBP2, STX2
	Equus caballus (horse)	8	NC_009151.2	TMEM132B, TMEM132C, SLC15A4, GLT1D1, *, FZD10, PIWIL1, RIMBP2, STX2
	Felis catus (domestic cat)	D3	NC_018734.2	AACS, TMEM132C, SLC15A4, GLT1D1, *, FZD10, PIWIL1, RIMBP2, STX2
	Homo sapiens (human)	12	NC_000012.12	TMEM132B, TMEM132C, SLC15A4, GLT1D1, *, FZD10, PIWIL1, RIMBP2, STX2
TP53BP1	Bos taurus (cattle)	21	AC_000178.1	LCMT2, ADAL, ZSCAN29, TUBGCP4, *, MAP1A, PPIP5K1, CKMT1B, STRC
	Canis lupus familiaris (dog)	30	NC_006612.3	LCMT2, ADAL, ZSCAN29, TUBGCP4, *, MAP1A, PPIP5K2, STRC
	Equus caballus (horse)	1	NC_009144.2	LCMT2, ADAL, ZSCAN29, TUBGCP4, *, MAP1A, PPIP5K1, CKMT1A, STRC
	Felis catus (domestic cat)	B3	NC_018728.2	LCMT2, ADAL, ZSCAN29, TUBGCP4, *, MAP1A, PPIP5K1, CKMT1A, STRC
	Homo sapiens (human)	15	NC_000015.10	LCMT2, ADAL, ZSCAN29, TUBGCP4, *, MAP1A, PPIP5K1, CKMT1B, STRC

Gene	Organism	Chromosome	NCBI Accession	Adjacent Protein-coding genes
VEZT	Bos taurus (cattle)	5	AC_000162.1	GLYCAM1, MUCL1, USP44, METAP2,*, FGD6, NR2C1, NDUFA12, TMCC3
	Canis lupus familiaris (dog)	15	NC_006597.3	SNRPF, NTN4, USP44, METAP2, *, FGD6, NR2C1, NDUFA12, TMCC3
	Equus caballus (horse)	28	NC_009171.2	SNRPF, NTN4, USP44, METAP2, *, FGD6, NR2C1, NDUFA12, TMCC3
	Felis catus (domestic cat)	B4	NC_018729.2	SNRPF, NTN4, USP44, METAP2, *, FGD6, NR2C1, NDUFA12, TMCC3
	Homo sapiens (human)	12	NC_000012.12	SNRPF, NTN4, USP44, METAP2, *, FGD6, NR2C1, NDUFA12, TMCC3
ZMYND10	Bos taurus (cattle)	22	AC_000179.1	HYAL3, HYAL1, HYAL2, TUSC2, RASSF1, *, NPRL2, CYB561D2, TMEM115, CACNA2D2
	Canis lupus familiaris (dog)	20	NC_006602.3	HYAL3, HYAL1, HYAL2, TUSC2, RASSF1, *, NPRL2, CYB561D2, TMEM115, CACNA2D2
	Equus caballus (horse)	16	NC_009159.2	HYAL3, HYAL1, HYAL2, TUSC2, RASSF1, *, NPRL2, TMEM115, CACNA2D2, C16H3orf18
	Felis catus (domestic cat)	A2	NC_018724.2	NAT6, HYAL3, TUSC2, RASSF1, *, NPRL2, TMEM115, CACNA2D2, CA2H3orf18
	Homo sapiens (human)	3	NC_000003.12	HYAL3, HYAL1, HYAL2, TUSC2, RASSF1, *, NPRL2, CYB561D2, TMEM115, CACNA2D2
ZNF521	Bos taurus (cattle)	26	NC_007325.6	CABYR, OSBPL1A, IMPACT, HRH4, *, SS18, PSMA8, TAF4B, KCTD1
	Canis lupus familiaris (dog)	7	NC_006589.3	CABYR, OSBPL1A, IMPACT, HRH4, *, SS18, PSMA8, TAF4B, KCTD1
	Equus caballus (horse)	8	NC_009151.2	CABYR, OSBPL1A, IMPACT, HRH4, *, SS18, PSMA8, TAF4B, KCTD1
	Felis catus (domestic cat)	D3	NC_018734.2	CABYR, OSBPL1A, IMPACT, HRH4, *, SS18, PSMA8, TAF4B, KCTD1
	Homo sapiens (human)	18	NC_000018.10	CABYR, OSBPL1A, IMPACT, HRH4, *, SS18, PSMA8, TAF4B, KCTD1

Notes

[1] SELENOK = SELK

[2] COA5 deleted or absent from the assembly

[3] AKAIN1 = C18orf42

[4] LRP1B deleted or absent from the assembly

[5] MOB4 present as HSPE1-MOB4

[6] MOB4 deleted or absent from the assembly

[7] NSD3 = WHSC1L1

[8] TAS2R16 deleted or absent from the assembly
